# A Bottom-Up Synthetic Approach to Polyethylene Furanoate Model Compounds

**DOI:** 10.1021/acs.joc.5c02656

**Published:** 2026-01-06

**Authors:** Koushik Mondal, Jyothis Dharaniyedath, Bernd H. Müller, Anke Spannenberg, Marcus Klahn, Robert Francke

**Affiliations:** 28392 Leibniz Institute for Catalysis, Albert-Einstein-Strasse 29a, 18059 Rostock, Germany

## Abstract

The growing importance of polyethylene furanoate (PEF) as a potential biobased substitute for PET is sparking interest in developing strategies for the chemical recycling of PEF. Access to appropriate model compounds is desirable for the development of depolymerization methods as well as for studying molecular properties and reactivity of PEF models. Herein, we describe a bottom-up strategy for the efficient and selective preparation of specific chain fragments from 2,5-furandicarboxylic acid (FDCA) and monoethylene glycol (MEG). Since conventional esterification methods according to Fischer and Steglich proved to be ineffective for certain target compounds, a sequence that consists of one-sided FDCA protection, Mukaiyama esterification, and subsequent deprotection was developed. The strategy was successfully applied to the preparation of various model compounds, including examples that have not yet been described in the literature.

## Graphical abstract

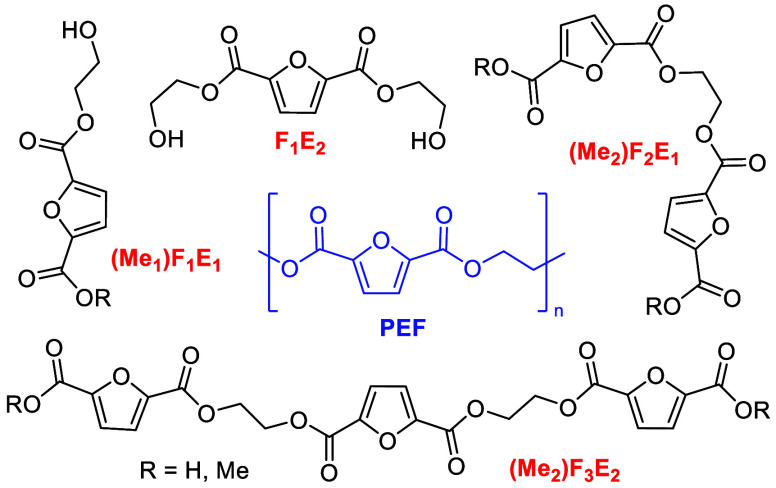

## Introduction

Major challenges are associated with the widespread use of plastics, i.e., environmental contamination through nonbiodegradable particles, immense greenhouse gas emissions associated with the life cycle of the materials, and consumption of finite fossil feedstocks for production.^1,2^ Consequently, intensive research is being conducted into new re- and upcycling strategies, as well as the development of alternative polymers that can be synthesized from renewable feedstocks.^3–6^ A prominent example is polyethylene terephthalate (PET), which is widely used in textile fibers, bottles, and thin films.^7,8^ While a large number of studies are dealing with chemical recycling processes,^9–11^ further efforts are being made to synthesize the required building blocks from renewable raw materials^12^ or to replace PET through materials with a better ecological footprint.

A candidate for replacing PET that is currently attracting a significant amount of attention is polyethylene furanoate (PEF (Figure 1)), a material with comparable mechanical, surface, and barrier properties.^13,14^ A key advantage is that both required building blocks, monoethylene glycol (MEG) and 2,5-furandicarboxylic acid (FDCA), can be produced from plant-based sugars using established processes.^15^ In parallel to scaling up PEF production and establishing it on the market, developing efficient end-of-life strategies in line with the principle of the circular economy has become a priority.^16^ Chemical conversion of PEF waste to recover monomers or extract valuable raw material waste has recently been investigated using base- and heat-induced,^17–20^ mechanochemical,^21^ and (bio)­catalytic approaches.^22–26^ Further studies addressed the effects of deep eutectic solvents and ionic liquids on depolymerization.^27,28^ The resulting product mixtures usually contain not only MEG and FDCA but also larger chain fragments that can be characterized by mass spectrometric methods. Bottom-up synthetic protocols to synthesize PEF fragments such as **F**_**1**_ **E**_**1**_, **F**_**2**_ **E**_**1**_, and **F**_**3**_ **E**_**2**_, as well as compounds **Me**_**2**_ **F**_**2**_ **E**_**1**_ and **Me**_**2**_ **F**_**3**_ **E**_**2**_ that can in principle be obtained from methanolysis, are desirable regarding their use as a standard for quantification and as model compounds for studies of chemical properties. Interestingly, the synthesis of such model compounds has not yet been described in the literature. Herein, we present the first synthetic access to the above-mentioned PEF-derived compounds (Figure 1). The scope of the protocol was also tested with respect to poly­(butylene furanoate) (PBF) fragment **F_1_B_1_**.

**Figure 1:**
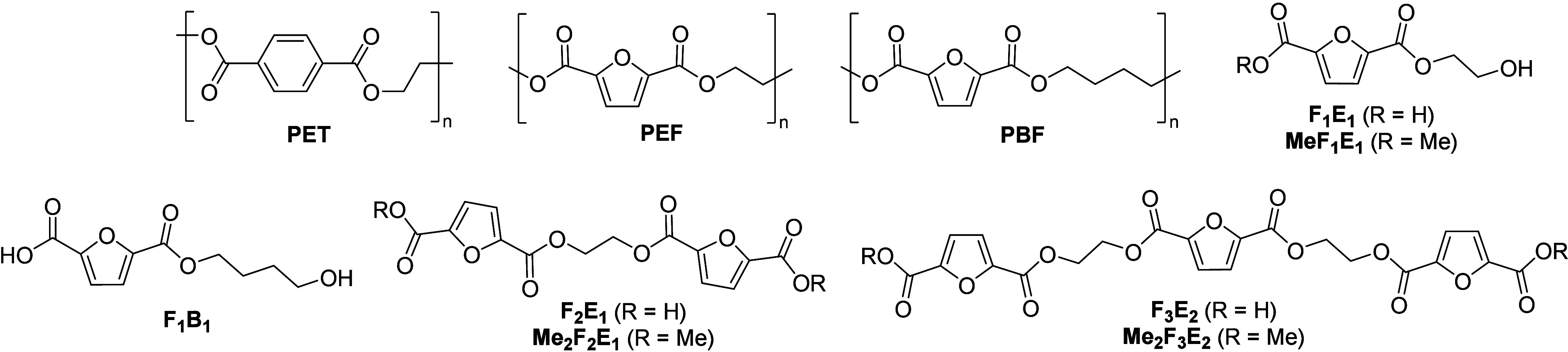
Polyethylene terephthalate (PET), its potential biobased substitutes polyethylene furanoate (PEF) and polybutylene furanoate (PBF), and model compounds developed in this work.

## Results and Discussion

The synthesis of PEF fragment **F**_**1**_ **E**_**2**_ via Fischer esterification (Scheme 1), already known in the literature,^29^ was achieved by us via a slightly modified procedure with a 83% yield. In this case, MEG is used in large excess, acting both as a reactant and as a solvent. However, the production of **F**_**2**_ **E**_**1**_, which, in principle, requires an excess of FDCA, does not work in an analogous fashion. Thus, the reaction of MEG with 2 equiv of FDCA in toluene under reflux in the presence of catalytic amounts of H_2_SO_4_ did not lead to the formation of the desired product. Subsequent efforts were focused on Steglich esterification using DMAP as a catalyst in combination with carbodiimide-based coupling reagents. To our surprise, this approach remained unsuccessful as well due to incomplete conversion and the coprecipitation of **F**_**2**_ **E**_**1**_ with **F**_**1**_ **E**_**1**_ and reagent waste, resulting in difficult separation of the product mixture. Consequently, traditional Steglich and Fischer esterification methods do not appear to be suitable for synthesizing FDCA-capped PEF model compounds. A detailed overview of the preliminary tests can be found in Supporting Information (Tables S1 and S2).

**Scheme 1:**
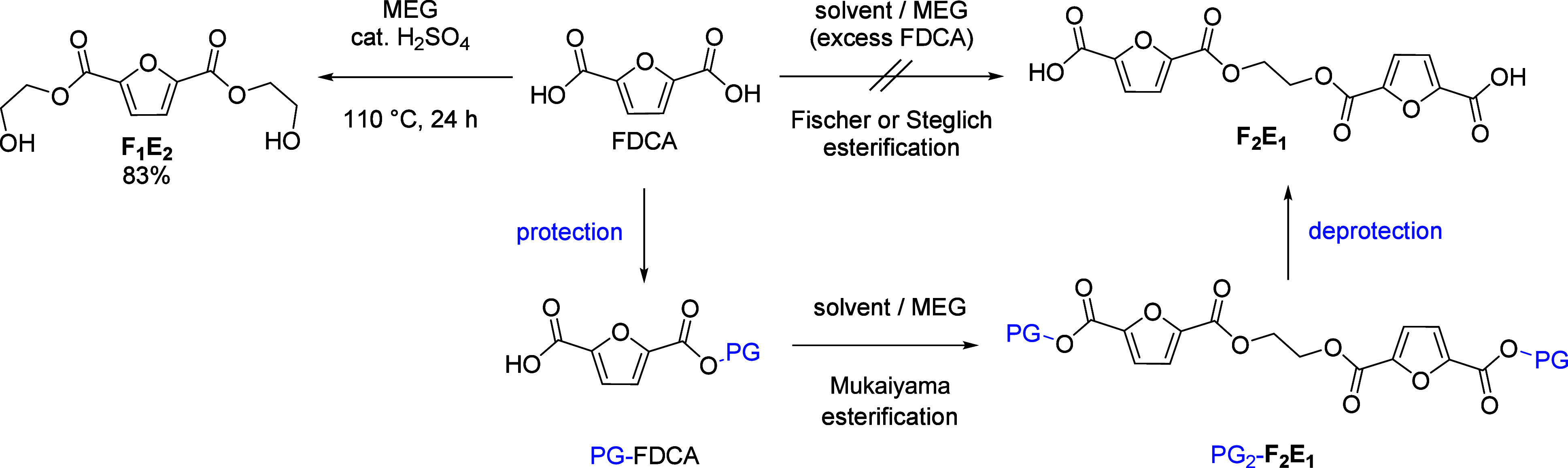
General Strategy for the Synthesis of PEF Model Compounds Using the Example of **F**_**2**_ **E**_**1**_ ^a^

a Attempts to synthesize **F**_**2**_ **E**_**1**_ directly from FDCA under Fischer (catalyst H_2_SO_4_, reflux, Dean-Stark trap) or Steglich conditions (DCC, DMAP) are summarized in the Supporting Information.

In view of the negative results, we turned to a new strategy in which one carboxyl group of FDCA is protected, followed by Mukaiyama esterification^30^ with MEG, and deprotection in the final step (Scheme 1). When the protecting group is selected, care must be taken to ensure that the MEG diester motif remains intact during deprotection. In view of the promising atom economy, convenient removal, and orthogonality to other ester groups,^31^ we first tested the *tert*-butyl group and found that it fulfills all requirements (*vide infra*).

Monoprotected FDCA derivative **1** was synthesized in three steps by adapting a protocol for the preparation of terephthalic acid mono-*tert*-butyl ester (Scheme 2).^32^ The sequence started with the synthesis of the acid chloride (FDCA-Cl_2_) using SOCl_2_, followed by double esterification with *t*-BuOH and partial deprotection under alkaline conditions. Compound **1** was obtained in a 43% yield over three steps, along with significant amounts of unconverted di-*tert*-butyl ester and FDCA. In a separate attempt, compound **1** was synthesized by monoesterification with 1 equiv of *t*-BuOH from FDCA-Cl_2_, whereby a merely 35% overall yield was achieved (for details, see the Supporting Information).

**Scheme 2:**
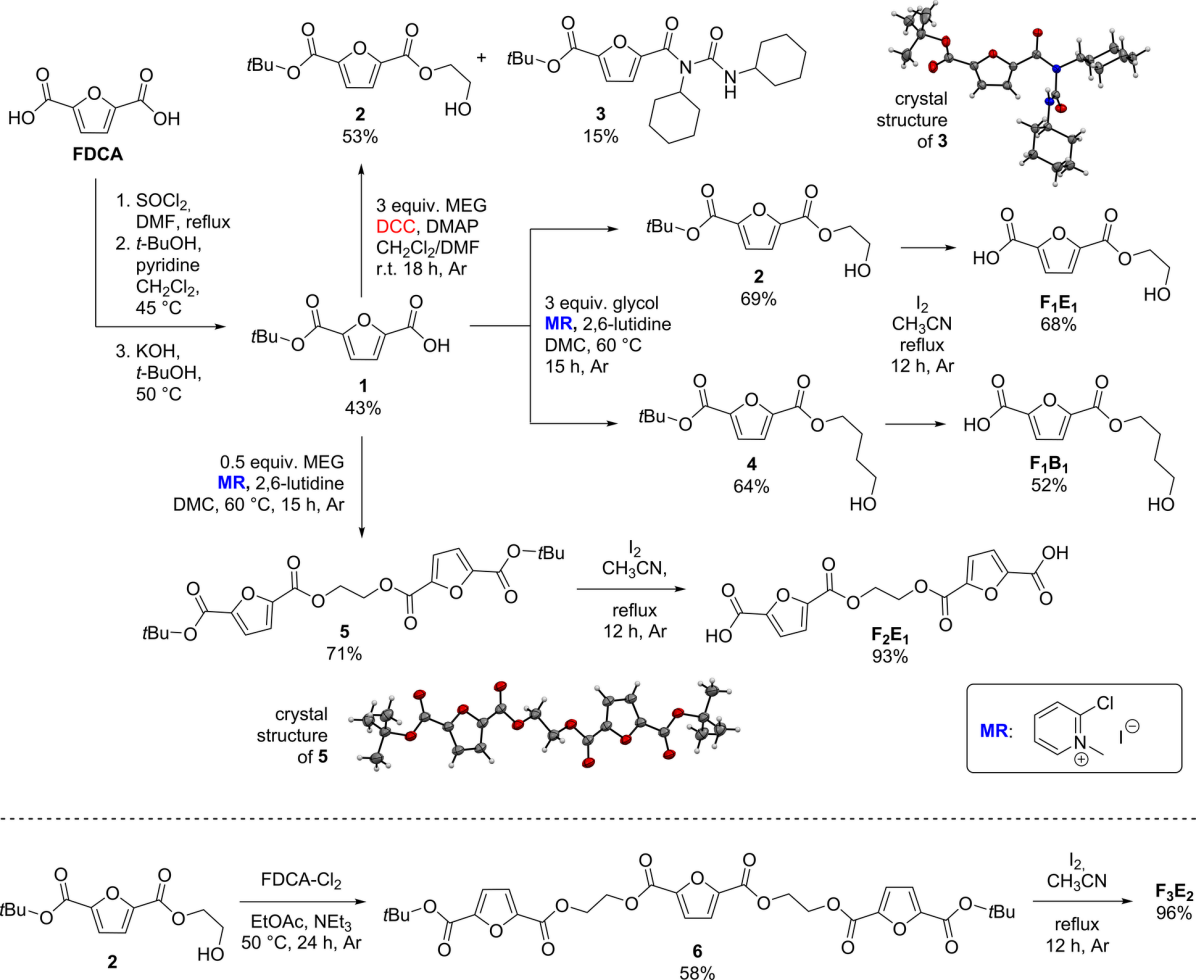
Synthesis of Model Compounds **F**_**1**_ **E**_**1**_, **F**_**1**_ **B**_**1**_, **F**_**2**_ **E**_**1**_, and **F**_**3**_ **E**_**2**_ from FDCA^a^

a Results of single-crystal X-ray analysis are inserted as displacement ellipsoid plots (50% probability level) of **5** and of one molecule of the asymmetric unit of **3** (for crystallographic details, see the Supporting Information).

Next, esterification of **1** with MEG (3 equiv) was attempted using DCC, 10 mol % DMAP, and a 1:1 (v/v) CH_2_Cl_2_/DMF mixture as the reaction medium (Scheme 2).^33^ However, after 18 h, the desired product **2** could be isolated in an only 53% yield, while *N*-acylurea derivative **3** was formed as a side product (15% yield). The molecular structure of **3** was confirmed by single-crystal X-ray analysis (for details, see the Supporting Information). In Steglich esterifications, such *N*-acylurea compounds are sometimes generated via slow rearrangement of the more reactive *O*-acylisourea intermediates, particularly upon conversion of aromatic carboxylic acids and less nucleophilic alcohols.^34^ In our case, the formation of **3** complicated the separation of the product mixture significantly. Since using the related EDC reagent or other solvents did not lead to any improvements, Mukaiyama’s reagent (MR) was tested in combination with 2,6-lutidine and dimethyl carbonate (DMC) as the solvent,^35,36^ providing MEG ester **3** in 69% yield (Scheme 2), along with MEG diester **5** as an easily separable side product (17% isolated yield). Subsequent deprotection of the *tert*-butylester group with 30 mol % I_2_ ^31^ provided **F**_**1**_ **E**_**1**_ in 68% yield. The same approach proved to be successful for esterification of **1** with butane-1,4-diol, yielding **4** (64%) and ultimately polybutylene furanoate fragment **F**_**1**_ **B**_**1**_ (52%) after deprotection.

The strategy was also applied to the synthesis of the **F**_**2**_ **E**_**1**_ model compound starting from **1**, whereby the number of MEG equivalents was reduced from 3 to 0.5 to promote selectivity for diesterification (Scheme 2). As a result, compound **5** was obtained in a 71% yield, whereby compound **2** was formed as a side product (21% yield). The molecular structure of **5** was corroborated by single-crystal X-ray analysis (for details, see the Supporting Information). After the deprotection of **5**, **F**_**2**_ **E**_**1**_ precipitated from the reaction mixture and was collected by filtration (93% yield).

With model compound **F**_**3**_ **E**_**2**_ as the target, compound **2** was reacted with **FDCA-Cl**_**2**_. EtOAc as the solvent, NEt_3_ as the base, and a slightly increased temperature (50 °C) proved to be suitable conditions under which **6** could be obtained in a 58% yield. Subsequent deprotection with I_2_ in CH_3_CN yielded **F**_**3**_ **E**_**2**_, which precipitated from the reaction mixture and could be isolated in a 96% yield.

It should be noted that all reactions starting from FDCA and leading to the model compounds were carried out under an Ar atmosphere, since partial oxidation of FDCA derivatives under aerobic conditions has been described in the literature.^29^ In our case, slow brownish coloration of the reaction mixture was observed upon exposure to air, resulting in a slight coloring of the products (even though no impurities were detected in the ^1^H NMR spectra).

Synthesis of PEF model methanolysates was carried out in a similar manner using commercially available FDCA dimethyl ester (**7**) as the starting material (Figure 2). Partial ester cleavage under alkaline conditions rendered **8**, which was converted with 0.5 equiv of MEG into **Me**_**2**_ **F**_**2**_ **E**_**1**_. The desired product was obtained in a 69% yield along with **Me_1_F**_**1**_ **E**_**1**_ as a side product (22% yield). On the other hand, conversion of **8** with a 3-fold excess of MEG rendered **Me**_**1**_ **F**_**1**_ **E**_**1**_ in a 66% yield. Finally, the reaction with FDCA-Cl_2_ in the presence of NEt_3_ gave **Me**_**2**_ **F**_**3**_ **E**_**2**_ (90% yield).

**Figure 2:**
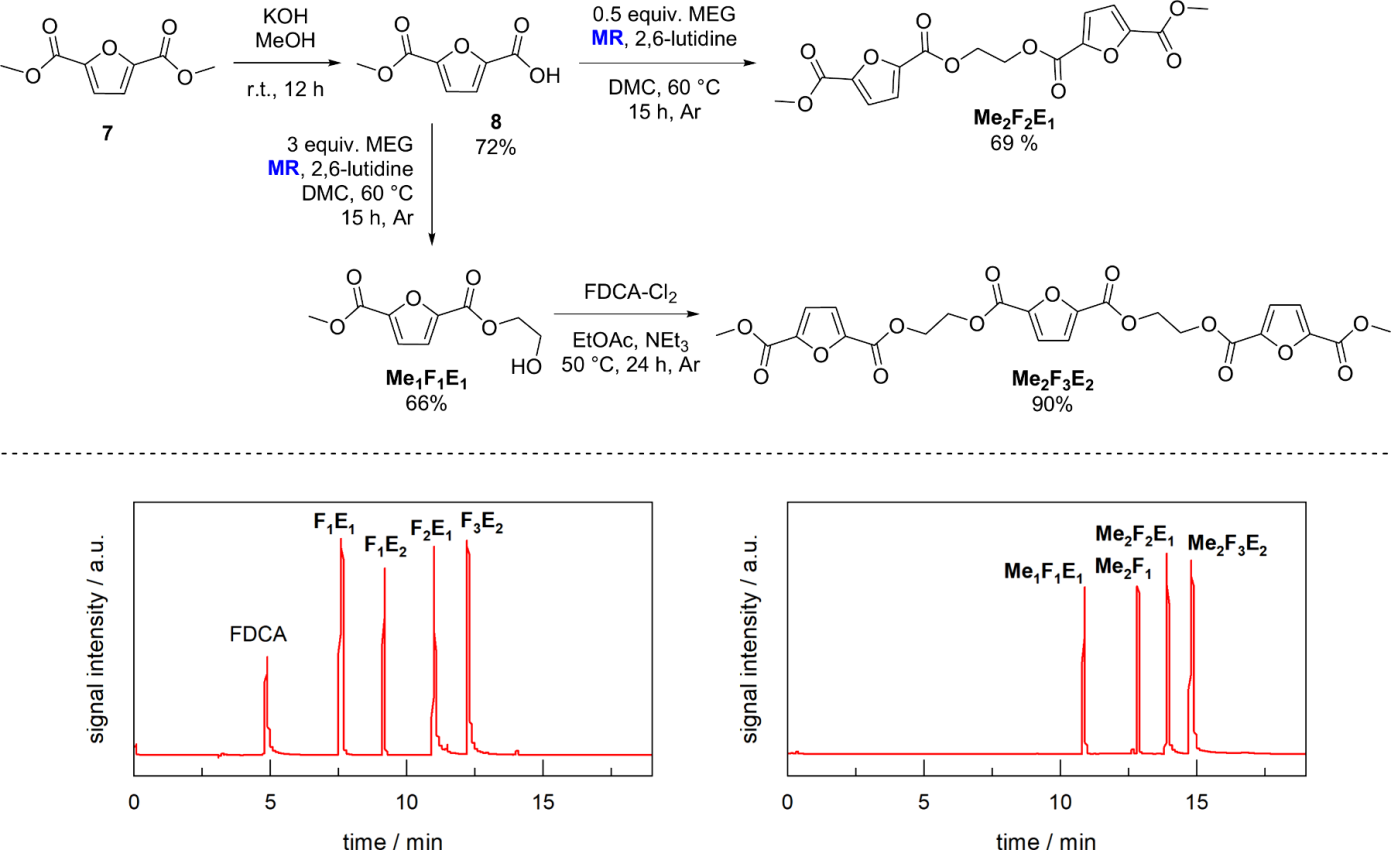
Synthesis of the methyl-terminated PEF model fragments **Me**_**1**_ **F**_**1**_ **E**_**1**_, **Me**_**2**_ **F**_**2**_ **E**_**1**_, and **Me**_**2**_ **F**_**3**_ **E**_**2**_ (top). Chromatograms resulting from HPLC analysis of mixtures of model hydrolysates (left) and methanolysates (right) (bottom).

Based on a previous report,^23^ a protocol was developed for HPLC analysis of product mixtures that are expected to result from incomplete depolymerization of PEF. To achieve good chromatographic separation of the compounds, an eluent consisting of formic acid (0.1% (v/v)) in a water/acetonitrile mixture (gradient elution) was used in combination with a nonpolar C18 HPLC column. A detailed description of the method can be found in the Supporting Information. The results are shown in Figure 2 (bottom) for the model hydrolysates (left) and methanolysates (right).

Finally, the solubility of the synthesized hydrolysate and methanolysate model fragments in different solvents was analyzed. **F**_**1**_ **E**_**1**_ and **F**_**1**_ **E**_**2**_ were found to be very soluble in common organic solvents and water. All hydrolysate models are quite soluble in DMSO and to a certain extend also in an aqueous phosphate buffer solution (pH 7.5) and in 1,1,1,3,3,3-hexafluoroisopropanol (HFIP). In other solvents, the solubilities of **F**_**2**_ **E**_**1**_ and **F**_**3**_ **E**_**2**_ are significantly decreased. Contrary behavior is observed for methyl-capped models **Me**_**2**_ **F**_**2**_ **E**_**1**_, and **Me**_**2**_ **F**_**3**_ **E**_**2**_, which show poor solubility in DMSO but good solubility in less polar solvents such as CH_2_Cl_2_ and CHCl_3_. A detailed overview of the results of the solubility study can be found in Table S5.

## Conclusion

In summary, an efficient and reliable synthetic bottom-up approach to PEF model compounds was developed that enables selective preparation of MEG- and FDCA-terminated chain fragments that can be expected from hydrolytic and methanolytic depolymerization. Our studies have shown that classic Steglich and Fischer esterifications are ineffective for this purpose, calling for single-sided protection of FDCA and esterification of the remaining carboxyl group with MEG using the Mukaiyama reagent. This strategy was successfully applied to the synthesis of various model compounds, including four examples that are not yet described in the literature (**F**_**2**_ **E**_**1**_, **F**_**3**_ **E**_**2**_, **Me**_**2**_ **F**_**2**_ **E**_**1**_, and **Me**_**2**_ **F**_**3**_ **E**_**2**_). Furthermore, an HPLC method that allows for the separation of mixtures of chain fragments that are expected to result from incomplete PEF depolymerization is presented. Our work expands the tool box for PEF depolymerization studies and paves the way to kinetic analysis of bond cleavage, preparation of calibration standards for liquid chromatographic quantification, and synthesis of new polymers from depolymerization fragments as a chemical upcycling strategy.^20^ Related work is currently being carried out in our laboratory and will be reported soon.

## Supplementary Material





## Data Availability

The data underlying this study are available in the published article and its .

## Notes

FAIR data, including the primary NMR FID files, for compounds **1**–**8**, **FDCA-Cl**_**2**_, **F**_**1**_ **B**_**1**_, **F**_**1**_ **E**_**1**_, **F**_**1**_ **E**_**2**_, **F**_**2**_ **E**_**1**_, **F**_**3**_ **E**_**2**_, **Me**_**1**_ **F**_**1**_ **E**_**1**_, **Me**_**2**_ **F**_**2**_ **E**_**1**_, and **Me**_**2**_ **F**_**3**_ **E**_**2**_ (ZIP)
Experimental descriptions and synthetic protocols, compound characterization data, NMR spectra, crystallographic information, and details of the HPLC analysis of the PEF model compounds (PDF)

## References

[1] Chia R. W., Lee J.-Y., Kim H., Jang J. (2021). Microplastic pollution
in soil and groundwater: a review. Environ.
Chem. Lett..

[2] Nicholson S. R., Rorrer N. A., Carpenter A. C., Beckham G. T. (2021). Manufacturing energy
and greenhouse gas emissions associated with plastics consumption. Joule.

[3] Sousa A. F., Silvestre A. J. D. (2022). Plastics from renewable sources as
green and sustainable
alternatives. Curr. Opin. Green Sust..

[4] Grignard B., Gennen S., Jérôme C., Kleij A. W., Detrembleur C. (2019). Advances in the use of CO_2_ as a renewable
feedstock for the synthesis of polymers. Chem.
Soc. Rev..

[5] Coates G. W., Getzler Y. D. Y. L. (2020). Chemical recycling to monomer for an ideal, circular
polymer economy. Nat. Rev. Mater..

[6] Thiounn T., Smith R. C. (2020). Advances and approaches
for chemical recycling of plastic
waste. J. Polym. Sci..

[7] Dhaka V., Singh S., Anil A. G., Sunil Kumar Naik T. S., Garg S., Samuel J., Kumar M., Ramamurthy P. C., Singh J. (2022). Occurrence, toxicity and remediation
of polyethylene terephthalate
plastics. A review. Environ. Chem. Lett..

[8] Barnard E., Rubio Arias J. J., Thielemans W. (2021). Chemolytic depolymerisation of PET:
a review. Green Chem..

[9] Bohre A., Jadhao P. R., Tripathi K., Pant K. K., Likozar B., Saha B. (2023). Chemical Recycling
Processes of Waste Polyethylene Terephthalate
Using Solid Catalysts. ChemSusChem.

[10] Ghosal K., Nayak C. (2022). Recent advances in
chemical recycling of polyethylene terephthalate
waste into value added products for sustainable coating solutions
– hope vs. hype. Mater. Adv..

[11] Xin J., Zhang Q., Huang J., Huang R., Jaffery Q. Z., Yan D., Zhou Q., Xu J., Lu X. (2021). Progress in the catalytic
glycolysis of polyethylene terephthalate. J.
Environ. Manag..

[12] Kwon E. E., Lee J. (2024). Polyethylene terephthalate production
from a carbon neutral resource. J. Clean. Prod..

[13] Rosenboom J.-G., Hohl D. K., Fleckenstein P., Storti G., Morbidelli M. (2018). Bottle-grade
polyethylene furanoate from ring-opening polymerisation of cyclic
oligomers. Nat. Commun..

[14] Sanders J. H., Cunniffe J., Carrejo E., Burke C., Reynolds A. M., Dey S. C., Islam M. N., Wagner O., Argyropoulos D. (2024). Biobased Polyethylene
Furanoate: Production Processes, Sustainability, and Techno-Economics. Adv. Sustainable Syst..

[15] de
Jong E., Visser H. A., Dias A. S., Harvey C., Gruter G.-J. M. (2022). The
Road to Bring FDCA and PEF to the Market. Polymers.

[16] Silverwood L., Mottoul M., Dumont M.-J. (2024). A Review
of End-of-Life Pathways
for Poly­(Ethylene Furanoate) and its Derivatives. J. Polym. Environ..

[17] Dargó G., Kis D., Ráduly A., Farkas V., Kupai J. (2025). Furandicarboxylic
Acid (FDCA): Electrosynthesis and Its Facile Recovery From Polyethylene
Furanoate (PEF) via Depolymerization. ChemSusChem.

[18] Wang Y.-H., Lee H. L., Lee H.-M., Pratama D. E., Lee T. (2025). Process Development
and Optimization of Alkaline Hydrolysis for Depolymerization in Chemical
Recycling of Poly­(ethylene 2,5-furandicarboxylate) (PEF) Using Full
Factorial Design. Ind. Eng. Chem. Res..

[19] Ren L., Yang S., Wang J., Zhang T., Li X., Wang T., Zhao Y. (2023). Electrocatalytic valorization of
waste polyethylene furanoate (PEF) bioplastics for the production
of formic acid and hydrogen energy. React. Chem.
Eng..

[20] Foltýn T., Všetečka J., Svoboda R., Podzimek Š., Vinklárek J., Honzíček J. (2025). Depolymerized
Poly­(ethylene-2,5-furanoate) as a Sustainable Feedstock for Biobased
Unsaturated Polyester Resins. Macromolecules.

[21] Jain D., Cramer F., Shamraienko P., Drexler H.-J., Voit B., Beweries T. (2025). Highly efficient mechanochemical depolymerisation of
bio-based polyethylene furanoate and polybutylene furanoate. RSC Sustainability.

[22] Kumar V., Pellis A., Wimmer R., Popok V., Christiansen J. d. C., Varrone C. (2024). Efficient Depolymerization
of Poly­(ethylene 2,5-furanoate)
Using Polyester Hydrolases. ACS Sustainable
Chem. Eng..

[23] Heinks T., Hofmann K., Zimmermann L., Gamm I., Lieb A., Blach L., Wei R., Bornscheuer U. T., Thiele J., Hamel C. (2025). Analysis
of the product-spectrum
during the biocatalytic hydrolysis of PEF (poly­(ethylene furanoate))
with various esterases. RSC Sustainability.

[24] Gabirondo E., Melendez-Rodriguez B., Arnal C., Lagaron J. M., Martínez
de Ilarduya A., Sardon H., Torres-Giner S. (2021). Organocatalyzed
closed-loop chemical recycling of thermo-compressed films of poly­(ethylene
furanoate). Polym. Chem..

[25] Pellis A., Haernvall K., Pichler C. M., Ghazaryan G., Breinbauer R., Guebitz G. M. (2016). Enzymatic hydrolysis of poly­(ethylene
furanoate). J. Biotechnol..

[26] Weinberger S., Canadell J., Quartinello F., Yeniad B., Arias A., Pellis A., Guebitz G. M. (2017). Enzymatic
Degradation of Poly­(ethylene
2,5-furanoate) Powders and Amorphous Films. Catalysts.

[27] Agostinho B., Silvestre A. J. D., Sousa A. F. (2022). From PEF to rPEF: disclosing the
potential of deep eutectic solvents in continuous de-/re-polymerization
recycling of biobased polyesters. Green Chem..

[28] Qu X., Zhou G., Wang R., Yuan B., Jiang M., Tang J. (2021). Synergistic catalysis
of imidazole acetate ionic liquids for the
methanolysis of spiral poly­(ethylene 2,5-furandicarboxylate) under
a mild condition. Green Chem..

[29] Raboni F., Oliveri A., Rocca V. M., Moni L., Kumar V., Varrone C., Pellis A. (2025). On the Environmentally
Friendly Synthesis
of 2-Hydroxyethyl Furan-5-Carboxylic Acid (MHEF) and bis­(2-Hydroxyethyl)
Furan-2,5-Dicarboxylate (BHEF). Chem. Open.

[30] Li, J. J. Mukaiyama esterification. In Name Reactions: A Collection of Detailed Reaction Mechanisms; Li, J. J. , Ed.; Springer, 2003; pp 275–276.

[31] Yadav J. S., Balanarsaiah E., Raghavendra S., Satyanarayana M. (2006). Chemoselective
hydrolysis of *tert*-butyl esters in acetonitrile using
molecular iodine as a mild and efficient catalyst. Tetrahedron Lett..

[32] Graffner-Nordberg M., Marelius J., Ohlsson S., Persson Å., Swedberg G., Andersson P., Andersson S. E., Åqvist J., Hallberg A. (2000). Computational Predictions
of Binding Affinities to
Dihydrofolate Reductase: Synthesis and Biological Evaluation of Methotrexate
Analogues. J. Med. Chem..

[33] Pham P. H., Barlow S., Marder S. R., Luca O. R. (2023). Electricity-driven
recycling of ester plastics using one-electron electro-organocatalysis. Chem. Catal..

[34] Steglich B. N. W. (1985). Esterification
of Carboxylic Acids with Dicyclohexalcarbodiimide/4-Dimethylaminopyridine:
tert-Butyl Ethyl Fumarate. Org. Synth..

[35] Mukaiyama T., Usui M., Shimada E., Saigo K. (1975). A Convenient Method
For The Synthesis of Carboxylic Esters. Chem.
Lett..

[36] Jordan A., Whymark K. D., Sydenham J., Sneddon H. F. (2021). A solvent-reagent
selection guide for Steglich-type esterification of carboxylic acids. Green Chem..

