# Supplementary material for: A Bottom-Up Synthetic Approach to Polyethylene Furanoate Model Compounds
Source: J Org Chem. 2026 Jan 6;91(2):1130–5. doi: 10.1021/acs.joc.5c02656 (PMC12814528; doi:10.1021/acs.joc.5c02656)

Supporting Information

A Bottom-up Synthetic Approach to Polyethylene Furanoate Model Compounds

Koushik Mondal, Jyothis Dharaniyedath, Bernd H. Müller, Anke Spannenberg, Marcus Klahn, Robert Francke*

Leibniz Institute for Catalysis, Albert-Einstein-Str. 29a, 18059 Rostock, Germany.
* Corresponding author. E-mail: robert.francke@catalysis.de.

Content

1. Experimental section ... S2
- 1.1 General remarks ... S2
- 1.2 Summary of the attempts for Fischer and Steglich esterification of FDCA ... S3
- 1.3 Synthesis of starting materials ... S5
- 1.4 Optimization of the esterification of **8** ... S9
- 1.5 Synthesis of hydrolysate model fragments ... S10
- 1.6 Preparation of methanolysate model fragments ... S15
- 1.7 Isolation and characterization of *N*-Acylurea compound **3** ... S17
- 1.8 Solubility studies ... S18

2. Supplementary data ... S19
- 2.1 X-ray crystal structure analysis of compounds **3** and **5**. ... S19
- 2.2 Comparison between NMR spectra of PEF model hydrolysates ... S21
- 2.3 NMR spectra of synthesized compounds ... S23
- 2.4 HPLC Analysis ... S40

1. Experimental section

1.1 General remarks

^{1}H and ^{13}C NMR spectra were recorded using an AVANCE 300 or 400 spectrometer (Bruker). Chemical shifts (δ) are reported in parts per million (ppm) with the residual solvent peak as an internal reference. Flash chromatography was performed in Büchi Pure C-810 Flash system using FlashPure EcoFlex silica gel (40-63 µm) as support. High resolution mass spectrometry (HRMS) was carried out with time-of-flight electrospray ionization (ESI-TOF) using a UPLC H-Class/XEVO G2-XS (Waters Acquity). All starting materials were purchased from BLDpharm, Sigma-Aldrich, TCI, VWR, Thermo-Fisher or Alfa Aesar and used without further purification.

1.2 Summary of the attempts for Fischer and Steglich esterification of FDCA

Fischer esterification of FDCA and MEG was attempted by stirring a mixture of FDCA, MEG, and H_2SO_4 in a solvent under reflux conditions (for specifications, see Table S1). After the reaction time indicated, the solvent was removed under reduced pressure. The residue was dissolved in DMSO and 1,3,5-trimethoxybenzene added as internal standard. An aliquot was taken from the solution, mixed with a small amount of DMSO-d_6, and subjected to ^{1}H NMR spectroscopic analysis. In none of the cases, formation of $\mathbf{F_2E_1}$ was observed.

Table S1. Attempted synthesis of $\mathbf{F_2E_1}$ *via* Fischer esterification of FDCA with MEG.

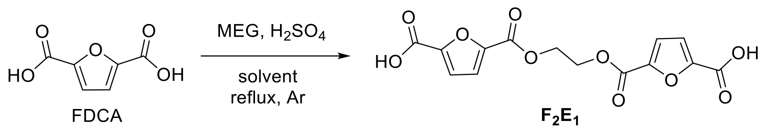

Entry	Solvent	FDCA	MEG	H_2SO_4	Time (h)	Yield of F_2E_1 [c]
1	benzene (6 mL)	156.1 mg (1.0 mmol)	14.00 µL (0.25 mmol)	1 drop	18	n.d.
2	toluene (6 mL)	156.1 mg (1.0 mmol)	14.00 µL (0.25 mmol)	1 drop	18	n.d.
3[a]	toluene (150 mL)	9.37 g (60.0 mmol)	1.68 mL (30.0 mmol)	0.9 mL	48	n.d.
4[b]	toluene (20 mL)	624.4 mg (4.0 mmol)	112.00 µL (2 mmol)	4 drops	48	n.d.

[a] A Dean-Stark apparatus was used. [b] Modified Dean-Stark apparatus suitable for small scale was used.[1] [c] Conversion was monitored by ^{1}H-NMR spectroscopic analysis of the crude reaction mixture using 1,3,5-trimethoxybenzene as internal standard.

[1] Wright, A. C.; Du, Y. E.; Stoltz, B. M. Small-Scale Procedure for Acid-Catalyzed Ketal Formation. *J. Org. Chem.* **2019**, *84*, 11258–11260. DOI: 10.1021/acs.joc.9b01541.

Steglich and Mukaiyama esterifications of FDCA with MEG were attempted by stirring a mixture of FDCA, MEG, coupling reagent and additive in a solvent at room temperature (for specifications, see Table S2). All the employed coupling reagents are known to form insoluble by-products. To analyze the liquid phase, an aliquot of the supernatant solution was taken, diluted with DMSO-d_6, and mixed with 1,3,5-trimethoxybenzene as internal standard. The solution was analyzed by ^{1}H NMR spectroscopy and in none of the cases, $\mathbf{F_2E_1}$ could be detected (footnote d in Table S2).

To achieve product analysis of the entire reaction mixture, the solvent was first removed under reduced pressure, followed by dissolution of the residue in a defined amount of DMSO and addition of 1,3,5-trimethoxybenzene as an internal standard (footnote e in Table S2). An aliquot was taken from the solution, mixed with a small amount of DMSO-d_6, and subjected to ^{1}H NMR spectroscopic analysis.

An attempt for Mukaiyama esterification of MEG and FDCA in DMSO shows that the reaction mixture remains homogeneous (no precipitation), whereby only slow conversion to $\mathbf{F_1E_1}$ was observed and $\mathbf{F_2E_1}$ could not be detected (Table S2, entry 4).

Taken together, these results suggest that conversion of FDCA with MEG under regular Steglich and Mukaiyama conditions is unsuitable for synthesis of $\mathbf{F_2E_1}$, as the desired product precipitates along with $\mathbf{F_1E_1}$ and the reagent waste, resulting in difficulties with separation.

Table S2. Attempted synthesis of $\mathbf{F_2E_1}$ *via* Steglich and Mukaiyama esterification of FDCA with MEG.

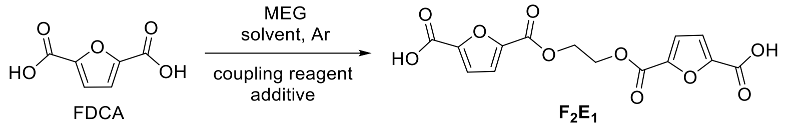

Entry	Solvent	FDCA	MEG	Coupling reagent	Additive	*T* / *t*	Yield $\mathbf{F_2E_1}$	Yield $\mathbf{F_1E_1}$
1	DCM : DMF 1:1, vol/vol (4 mL)	156.1 mg (1.0 mmol)	14.0 µL (0.25 mmol)	DCC[a] (113.5 mg, 0.55 mmol)	DMAP (6.1 mg, 0.05 mmol)	r.t. 18 h	n.d. [d] 34 [e]	n.d. [d] 21 [e]
2	DCM : DMF 1:1, vol/vol (4 mL)	156.1 mg (1.0 mmol)	14.0 µL (0.25 mmol)	EDC[b] (125.5 mg, 0.55 mmol)	DMAP (6.1 mg, 0.05 mmol)	r.t. 18 h	n.d. [d] 42 [e]	n.d. [d] 46 [e]
3	DMC (3 mL)	312.2 mg (2.0 mmol)	28.0 µL (0.5 mmol)	MR[c] (268.3 mg, 1.0 mmol)	2,6-lutidine (300.0 µL, 2.05 mmol)	60 °C 24 h	n.d. [d] 56 [e]	n.d. [d] 23 [e]
4	DMSO (3 mL)	312.2 mg (2.0 mmol)	28.0 µL (0.5 mmol)	MR[c] (268.3 mg, 1.0 mmol)	2,6-lutidine (300.0 µL, 2.05 mmol)	60 °C 24 h	n.d. [d,f]	18 [d,f]

[a] DCC = *N*, *N′*-dicyclohexylcarbodiimide. [b] EDC = *N*-ethyl-*N′*-(3-dimethylaminopropyl)carbodiimide hydrochloride. [c] MR = Mukaiyama's reagent. [d] Analysis of the liquid phase by ^{1}H-NMR spectroscopy using 1,3,5-trimethoxybenzene as internal standard. [e] Product analysis by ^{1}H NMR spectroscopy after removal of the solvent and dissolution of the residue in DMSO. [f] Reaction mixture remained homogeneous (no precipitates formed). Product analysis was performed by ^{1}H NMR spectroscopy directly from the reaction mixture.

1.3 Synthesis of starting materials

Furan-2,5-dicarbonyl dichloride

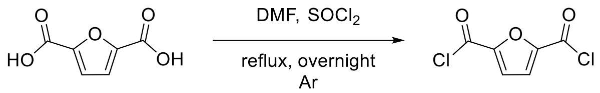

Furan-2,5-dicarbonyl dichloride was prepared according to a procedure reported in the literature.[2] To mixture of furan-2,5-dicarboxylic acid (FDCA, 10.0 g, 64.06 mmol) and dimethylformamide (DMF) (200 µL, 2.58 mmol), 20 mL of $SOCl_2$ was added slowly and the resulting mixture was stirred overnight under an Ar atmosphere under reflux conditions (oil bath as heat source). Excess $SOCl_2$ and DMF were removed under vacuum at room temperature and collected in a condensation trap cooled with liquid nitrogen. The resulting colorless crystalline solid was used directly for the next reaction without further purification.

^{1}H NMR (400 MHz, $CDCl_3$) δ 7.54 (s, 2H). ^{13}C{^{1}H} NMR (101 MHz, $CDCl_3$) δ 156.0, 149.5, 123.4. Spectral data are in agreement with the literature.[1]

Di-*tert*-butyl furan-2,5-dicarboxylate

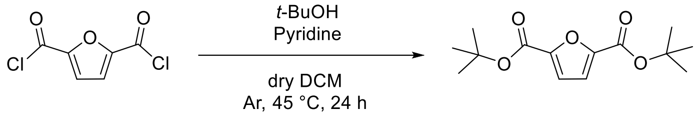

Di-*tert*-butyl furan-2,5-dicarboxylate was synthesized using a procedure reported in the literature.[3] Furan-2,5-dicarbonyl dichloride (12.36 g, 64.06 mmol) was dissolved in 35 mL of dry CH_2Cl_2 and added slowly to a solution of *tert*-butanol (18.5 mL, 194.93 mmol) and pyridine (11.35 mL, 140.93 mmol) in 35 mL of dry CH_2Cl_2.[3] The resulting mixture was heated under reflux conditions for 24 hours with continuous stirring under an Ar atmosphere (oil bath as heat source). Then, 20 mL of aq. 1M HCl were added and the layers separated. The aqueous layer was extracted with CH_2Cl_2 (3 x 60 mL) and the combined organic extracts treated with sat. sodium bicarbonate solution (2 x 40 mL), followed by drying over anhydrous Na_2SO_4 and evaporation of the solvent under reduced pressure. The product was obtained as a colorless solid (12.33 g, 45.95 mmol, 72% over two steps, starting from FDCA).[4]

^{1}H NMR (300 MHz, $CDCl_3$) δ 7.07 (s, 2H), 1.57 (s, 18H). ^{13}C{^{1}H} NMR (75 MHz, $CDCl_3$) δ 157.6, 147.8, 117.7, 82.9, 28.3. HRMS (ESI-TOF) m/z: calcd for $[C_{14}H_{20}O_5Na]^+$ 291.1203; found 291.1210. M.p. 117.0 – 117.9 °C.

---

[2] Gomes, M.; Gandini, A.; Silvestre, A. J. D.; Reis, B. Synthesis and characterization of poly(2,5-furan dicarboxylate)s based on a variety of diols. *J. Polym. Sci. A Polym. Chem.* **2011**, *49*, 3759-3768. DOI: 10.1002/pola.24812.

[3] Dry CH_2Cl_2 was purchased and used as received (AcroSeal™, 99.8 %, *extra dry*, over molecular sieve, stabilized).

[4] Graffner-Nordberg, M.; Marelius, J.; Ohlsson, S.; Persson, A.; Swedberg, G.; Andersson, P.; Andersson, S. E.; Aqvist, J.; Hallberg, A. Computational predictions of binding affinities to dihydrofolate reductase: synthesis and biological evaluation of methotrexate analogues. *J. Med. Chem.* **2000**, *43*, 3852-3861. DOI: 10.1021/jm0009639.

5-(*tert*-Butoxycarbonyl)furan-2-carboxylic acid (1)

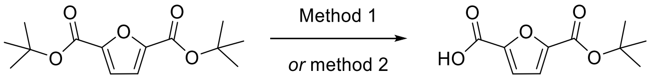

Method 1: Compound **1** was synthesized using a modified version of a procedure reported in the literature.[5] In a two-neck round-bottom flask, di-*tert*-butyl furan-2,5-dicarboxylate was dissolved in of *tert*-butanol at 30 °C and kept under an argon atmosphere. A solution of KOH in *tert*-butanol was prepared separately at 50 °C and then added dropwise. The resulting mixture was stirred at 50 °C for 12 h under Ar (oil bath as heat source). Excess *tert*-butanol was then removed under vacuum at room temperature and collected in a condensation trap cooled with liquid nitrogen. The crude product was purified either by column chromatography (batch 1) or by precipitation (batch 2, see Table S3).

Batch 1: The remaining solid was dissolved in CH_2Cl_2 and washed with aq. 1 M HCl (1 x 10 mL), dried over anhydrous Na_2SO_4 and concentrated under reduced pressure. The crude product was subjected to column chromatography over deactivated silica layer (eluent: CH_2Cl_2/MeOH 9:1, vol/vol).[6] The product was obtained as an oily substance, which was dissolved in 15 mL CH_2Cl_2, washed with aq. 1 M HCl (1 x 10 mL), dried over Na_2SO_4 and concentrated under reduced pressure. The additional step resulted in a colorless amorphous solid (61% yield). The diester starting material was isolated after column chromatography in a 29% yield.

Batch 2: The remaining solid was dissolved in CH_2Cl_2 and treated with sat. sodium bicarbonate solution (2 x 40 mL). The aqueous layer was brought to pH 4 using conc. aq. HCl and cooled in an ice bath for precipitation of the product. The precipitate was filtered off, washed repeatedly with cold distilled water for removal of FDCA which was formed as by-product. After drying in vacuum, the product was obtained as colorless amorphous solid in a 40% yield. Furthermore, the diester starting material was recovered by concentration of the combined organic layers under reduced pressure (23% yield).

Table S3. Two approaches to purification of compound **1**.

Batch	Starting material	KOH	Purification	Yield (product)	Yield (diester)
1	2.68 g (10.0 mmol) in 20 mL *t*-BuOH	0.56 g (10.0 mmol) in 20 mL *t*-BuOH	column	1.30 g<br>6.11 mmol<br>61%	0.77 g<br>2.88 mmol<br>29%
2	11.90 g (44.3 mmol) in 60 mL *t*-BuOH	2.49 g (44.3 mmol) in 60 mL *t*-BuOH	precipitation	3.78 g<br>17.8 mmol<br>40%	2.79 g<br>10.4 mmol<br>23%

[5] Graffner-Nordberg, M.; Marelius, J.; Ohlsson, S.; Persson, A.; Swedberg, G.; Andersson, P.; Andersson, S. E.; Aqvist, J.; Hallberg, A. Computational predictions of binding affinities to dihydrofolate reductase: synthesis and biological evaluation of methotrexate analogues. *J. Med. Chem.* **2000**, *43*, 3852-3861. DOI: 10.1021/jm0009639.
[6] The column was pre-treated (“deactivated”) by applying 2 mL NEt_3 to the column (approx. 50 g silica gel), followed by passing a small amount of eluent mixture.

Method 2: Furan-2,5-dicarbonyl dichloride (1.93 g, 10.0 mmol) was dissolved in 10 mL of dry CH_2Cl_2 and added slowly to a solution of *tert*-butanol (0.95 mL, 10.0 mmol) and pyridine (0.89 mL, 11.0 mmol) in 10 mL of dry CH_2Cl_2.[7] The resulting mixture was stirred under reflux conditions for 24 h under an Ar atmosphere (oil bath as heat source). After cooling to room temperature, 5 mL of aq. 1 M HCl were added and the layers separated. The aqueous layer was extracted with CH_2Cl_2 (3 x 15 mL), dried over anhydrous Na_2SO_4, and concentrated under reduced pressure. The crude product was subjected to flash column chromatography over deactivated silica (eluent: CH_2Cl_2/MeOH 9:1, vol/vol).[8] The product was obtained as an oily substance, which was dissolved in 10 mL CH_2Cl_2, washed with aq. 1 M HCl (1 x 5 mL), dried over Na_2SO_4 and concentrated under reduced pressure. This additional step rendered compound **1** as a colorless solid (746 mg, 3.52 mmol, 35%). The diester starting material was isolated after column chromatography (195 mg, 0.73 mmol, 15%). Furthermore, a significant amount of FDCA was also recovered, according to ^{1}H NMR spectroscopy as triethylammonium salt, whereby removal of NEt_3 and for isolation of FDCA was not attempted.

^{1}H NMR (400 MHz, DMSO-d_6) δ 7.16 (d, J = 3.5 Hz, 1H), 7.00 (d, J = 3.5 Hz, 1H), 1.51 (s, 9H). ^{13}C{^{1}H} NMR (101 MHz, DMSO-d_6) δ 160.7, 157.3, 152.3, 145.1, 118.5, 115.3, 81.8, 27.9. HRMS (ESI-TOF) m/z: calcd for $[C_{10}H_{11}O_5]^-$ 211.0612; found 211.0611. M.p. was not determined (sublimation prior to melting observed).

5-(Methoxycarbonyl)furan-2-carboxylic acid (8)

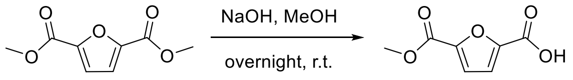

7 8

Compound **8** was synthesized according to a procedure reported in the literature.[9] Dimethyl furan-2,5-dicarboxylate **7** (2.76 g, 15.0 mmol) was dissolved in MeOH (80 mL). A solution of NaOH (0.60 mg, 15.0 mmol) in MeOH (20 mL) was added dropwise to the solution over 10 min and the resulting mixture was stirred overnight under an Ar atmosphere. The solvent was removed under reduced pressure and the residue dissolved in ethyl acetate (50 mL). The resulting solution was transferred to a separation funnel, followed by washing with aq. 1 M HCl (2 x 10 mL). The combined aqueous layers were extracted with ethyl acetate (1 x 20 mL). Then, the combined organic layers were washed with brine (1 x 20 mL), dried over Na_2SO_4 and concentrated under reduced pressure. The crude product was subjected to column chromatography on deactivated silica (eluent: CH_2Cl_2 /MeOH 23:2, vol/vol). The product was obtained as oily substance, which was dissolved in 15 mL ethyl acetate, washed with aq. 1 M HCl (1 x 5 mL), dried over Na_2SO_4, and concentrated under reduced pressure. The desired product was obtained as a colorless solid (1.83 g, 10.8 mmol, 72%). Unreacted dimethyl ester starting material was recovered after column chromatography (303 mg, 1.94 mmol, 13%).

---

[7] Dry CH_2Cl_2 was purchased and used as received (AcroSeal™, 99.8 %, *extra dry*, over molecular sieve, stabilized).

[8] The column was pre-treated ("deactivated") by applying 2 mL NEt_3 to the column (approx. 50 g silica gel), followed by passing a small amount of eluent mixture.

[9] Ward, L. C.; McCue, H. V.; Rigden, D. J.; Kershaw, N. M.; Ashbrook, C.; Hatton, H.; Goulding, E.; Johnson, J. R.; Carnell, A. J. Carboxyl Methyltransferase Catalysed Formation of Mono- and Dimethyl Esters under Aqueous Conditions: Application in Cascade Biocatalysis. *Angew. Chem. Int. Ed.* **2022**, *61*, e202117324. DOI: 10.1002/anie.202117324.

^{1}H NMR (400 MHz, DMSO-d_6) δ 7.38 (d, *J* = 3.6 Hz, 1H), 7.31 (d, *J* = 3.7 Hz, 1H), 3.85 (s, 3H). $^{13}C\{^{1}H\}$ NMR (101 MHz, DMSO-d_6) δ 158.8, 158.0, 147.5, 145.7, 119.0, 118.4, 52.3. Spectral data are in agreement with the literature. [10]

[10] Ward, L. C.; McCue, H. V.; Rigden, D. J.; Kershaw, N. M.; Ashbrook, C.; Hatton, H.; Goulding, E.; Johnson, J. R.; Carnell, A. J. Carboxyl Methyltransferase Catalysed Formation of Mono- and Dimethyl Esters under Aqueous Conditions: Application in Cascade Biocatalysis. *Angew. Chem. Int. Ed.* **2022**, *61*, e202117324. DOI: 10.1002/anie.202117324.

1.4 Optimization of the esterification of 8

Table S4. Optimization of solvent and coupling reagent for esterification of compound **8**.

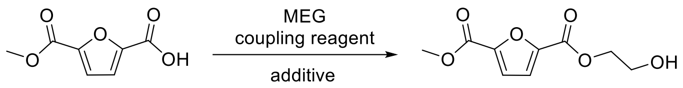

8 **MeF_1E_1**

Entry	Solvent	Reagent	Additive	Yield of MeF_1E_1[e]
1[a]	CH_2Cl_2/DMF 1:1 (v/v)	DCC	DMAP	26 (18[f])
2[a]	CH_2Cl_2	DCC	DMAP	19
3[a]	DCE[c]	DCC	DMAP	24
4[a]	CH_3CN	DCC	DMAP	26
5[a]	DMF	DCC	DMAP	n.d.
6[a]	EtOAc	DCC	DMAP	20
7[a]	DMC	DCC	DMAP	20
8[a]	CH_2Cl_2/DMF 1:1 (v/v)	EDC[d]	DMAP	18[f]
9[b]	DMC	MR	2,6-lutidine	66[f]

[a] 1.0 mmol **8**, 3.0 mmol MEG, 1.1 mmol coupling reagent, 0.1 mmol DMAP, 2 mL solvent, stirring at r.t. for 18 h. [b] 0.5 mmol **8**, 1.5 mmol MEG, 0.55 mmol Mukaiyama's reagent (MR), 1.05 mmol 2,6-lutidine, 1 mL solvent, stirring at 60 °C for 15 h. [c] DCE = 1,2-dichloroethane. [d] EDC = *N*-ethyl-*N′*-(3-dimethylaminopropyl)carbodiimide hydrochloride. [e] Yields were determined by ^{1}H-NMR analysis of the crude reaction mixture using 1,3,5-trimethoxybenzene as internal standard. [f] Isolated yield.

1.5 Synthesis of hydrolysate model fragments

2-(*tert*-Butyl) 5-(2-hydroxyethyl) furan-2,5-dicarboxylate (2)

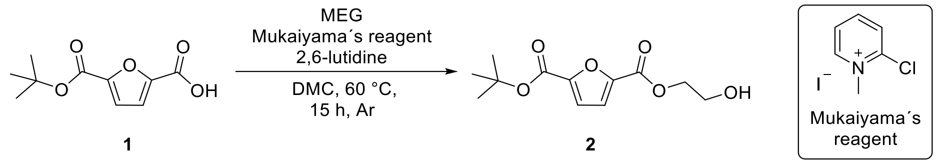

1 **2**

Compound **2** was synthesized using a modified version of a procedure reported in the literature.[11] A mixture of **1** (3.61 g, 17.0 mmol), monoethylene glycol (MEG, 2.85 mL, 51.0 mmol), Mukaiyama's reagent (4.83 g, 17.85 mmol), and 40 mL of dimethylcarbonate (DMC) was prepared. Then, 2,6-lutidine (4.05 mL, 34.85 mmol) was added and the resulting mixture was stirred under an Ar atmosphere for 15 h at 60 °C. After completed reaction, the mixture was diluted with ethyl acetate (50 mL) and filtered to remove the precipitated 1-methylpyridin-2(1*H*)-one. The filtrate was then washed with aq. 1 M HCl (1 x 15 mL) to remove 2,6-lutidine, dried over anhydrous Na_2SO_4, and concentrated under reduced pressure. The crude product was subjected to flash column chromatography for purification (eluent: *n*-heptane/ethyl acetate 13:7, vol/vol). The desired product was obtained as a pale-yellow solid (3.02 g, 11.77 mmol, 69%). Diester **5** was reisolated in a 17% yield (657 mg, 2.92 mmol).

^{1}H NMR (400 MHz, $CDCl_3$) δ 7.21 (d, *J* = 3.6 Hz, 1H), 7.08 (d, *J* = 3.6 Hz, 1H), 4.46 – 4.43 (m, 2H), 3.95 – 3.93 (m, 2H), 2.39 (s, 1H), 1.57 (s, 9H). ^{13}C{^{1}H} NMR (101 MHz, $CDCl_3$) δ 158.4, 157.4, 148.3, 146.2, 119.0, 117.6, 83.3, 67.1, 61.0, 28.2. HRMS (ESI-TOF) m/z: calcd. for $[C_{12}H_{16}O_6Na]^+$ 279.0839; found 279.0847. M.p. 72.2 – 74.8 °C.

5,5'-Di-*tert*-butyl $O^{\prime 2}$,O^{2}-(ethane-1,2-diyl) bis(furan-2,5-dicarboxylate) (5)

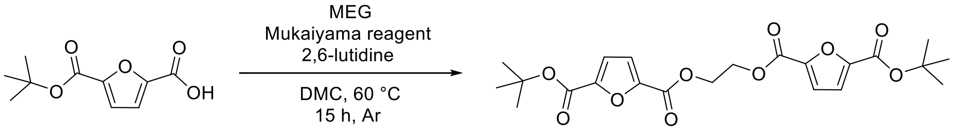

1 **5**

Compound **5** was synthesized using a modified version of a procedure reported in the literature.[11] A mixture of compound **1** (106 mg, 0.5 mmol), monoethylene glycol (MEG, 14.0 µL, 0.25 mmol), and Mukaiyama's reagent (149 mg, 0.55 mmol) in 1 mL of dimethylcarbonate (DMC) was prepared, to which 2,6-lutidine (0.12 µL, 1.05 mmol) was added. The resulting mixture was stirred under an Ar atmosphere for 15 h at 60 °C. After completed reaction, the mixture was diluted with 4 mL ethyl acetate and filtered to remove the precipitated 1-methylpyridin-2(1H)-one. The filtrate was washed with aq. 1 M HCl (1 x 2 mL), dried over anhydrous Na_2SO_4, and concentrated under reduced pressure. The crude product was subjected to flash column chromatography for further purification (eluent: *n*-heptane/ethyl acetate 5:1, vol/vol). The product was obtained as a pale-yellow solid (80 mg, 0.18 mmol, 71%). Compound **2** was isolated as a side product (27 mg, 0.10 mmol, 21%).

^{1}H NMR (400 MHz, $CDCl_3$) δ 7.21 (d, *J* = 3.6 Hz, 2H), 7.09 (d, *J* = 3.6 Hz, 2H), 4.63 (s, 4H), 1.58 (s, 18H). ^{13}C NMR (101 MHz, $CDCl_3$) δ 157.9, 157.4, 148.6, 145.8, 119.2, 117.7, 83.2,

[11] Jordan, A.; Whymark, K. D.; Sydenham, J.; Sneddon, H. F. A solvent-reagent selection guide for Steglich-type esterification of carboxylic acids. *Green Chem.* **2021**, *23*, 6405-6413. DOI: 10.1039/d1gc02251b.

62.9, 28.3. HRMS (ESI-TOF) m/z: calcd for $[C_{22}H_{26}O_{10}Na]^+$ 473.1418; found 473.1421. M.p. 158.5 – 160.1 °C.

The molecular structure was also confirmed using X-ray single crystal analysis (see section 2).

5,5'-Di-*tert*-butyl *O*'2,*O*2-(((furan-2,5-dicarbonyl)bis(oxy))bis(ethane-2,1-diyl)) bis(furan-2,5-dicarboxylate) (6)

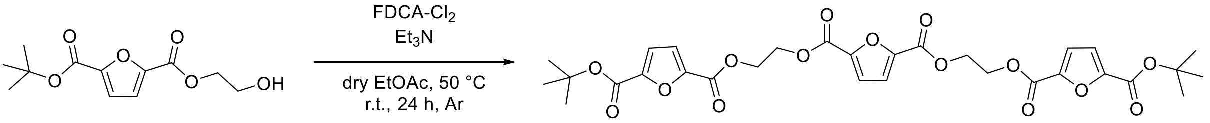

Compound **6** was synthesized using a modified version of a procedure reported in the literature.[12] In an oven-dried 100 mL Schlenk tube kept under an Ar atmosphere, compound **2** (1.54 g, 6.0 mmol) and furan-2,5-dicarbonyl dichloride (**FDCA-Cl₂**, 579.0 mg, 3 mmol) were dissolved in 50 mL of dry ethyl acetate.[13] Triethylamine (2.1 mL, 5 mmol) was added dropwise to the mixture, followed by stirring under an Ar atmosphere for 24 h at 50 °C. After completed reaction, the mixture was diluted with 10 mL ethyl acetate and washed with aq. 1 M HCl (1 x 10 mL), brine (1 x 10 mL), and dried over anhydrous Na_2SO_4. After removal of the solvent under reduced pressure, the crude product was subjected to flash column chromatography (eluent: CH_2Cl_2/MeOH 19:1, vol/vol). The desired product was obtained as colorless amorphous solid (1.10 g, 1.74 mmol, 58%).

^{1}H NMR (300 MHz, $CDCl_3$) δ 7.23 (s, 2H), 7.20 (d, *J* = 3.6 Hz, 2H), 7.09 (d, *J* = 3.6 Hz, 2H), 4.64 (s, 8H), 1.57 (s, 18H). $^{13}C\{^1H\}$ NMR (75 MHz, $CDCl_3$) δ 157.9, 157.7, 157.3, 148.6, 146.6, 145.8, 119.2, 119.1, 117.6, 83.2, 63.0, 62.8, 28.2. HRMS (ESI-TOF) m/z: calcd for $[C_{30}H_{32}O_{15}Na]^+$ 655.1633; found 655.1650. M.p. 182.3 – 183.5 °C.

2-(*tert*-Butyl) 5-(2-hydroxyethyl) furan-2,5-dicarboxylate (4)

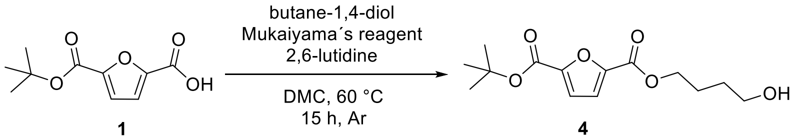

Compound **4** was synthesized using a modified version of a procedure reported in the literature.[14] To a mixture of **1** (424.40 mg, 2.0 mmol), butane-1,4-diol (532.0 µL, 6 mmol), and Mukaiyama's reagent (568.1 mg, 2.1 mmol) in 15 mL of dimethylcarbonate (DMC), 2,6-lutidine (476.0 µL, 4.1 mmol) was added and the resulting mixture was stirred under Ar atmosphere for 15 h at 60 °C. After completed reaction, the mixture was diluted with 20 mL ethyl acetate and filtered to remove the precipitated 1-methylpyridin-2(1*H*)-one. The filtrate was washed with

[12] Pham, P. H.; Barlow, S.; Marder, S. R.; Luca, O. R. Electricity-driven recycling of ester plastics using one-electron electro-organocatalysis. *Chem. Catal.* **2023**, *3*, 100675. DOI: 10.1016/j.checat.2023.100675.

[13] Dry ethyl acetate was purchased and used as received (AcroSeal, 99.9%, *extra dry*, over molecular sieve).

[14] Jordan, A.; Whymark, K. D.; Sydenham, J.; Sneddon, H. F. A solvent-reagent selection guide for Steglich-type esterification of carboxylic acids. *Green Chem.* **2021**, *23*, 6405-6413. DOI: 10.1039/d1gc02251b.

aq. 1 M HCl (1 x 5 mL) to remove 2,6-lutidine, dried over anhydrous Na_2SO_4, and concentrated under reduced pressure. The crude product was purified using flash column chromatography (eluent: *n*-heptane/ethyl acetate 13:7, vol/vol). The desired product was obtained as a pale-yellow solid (365.0 mg, 1.28 mmol, 64%).

^{1}H NMR (300 MHz, $CDCl_3$) δ 7.17 (d, *J* = 3.6 Hz, 1H), 7.08 (d, *J* = 3.6 Hz, 1H), 4.35 (t, *J* = 6.5 Hz, 2H), 3.70 (t, *J* = 6.3 Hz, 2H), 1.90 – 1.78 (m, 2H), 1.74 (s, 1H), 1.72 – 1.64 (m, 2H), 1.57 (s, 9H). ^{13}C{^{1}H} NMR (75 MHz, $CDCl_3$) δ 158.4, 157.4, 148.2, 146.5, 118.5, 117.6, 83.1, 65.4, 62.4, 29.2, 28.3, 25.2. HRMS (ESI-TOF) m/z: calcd. for $[C_{14}H_{20}O_6Na]^+$ 307.1152; found 307.1161. M.p. 44 – 47 °C.

General procedure for the deprotection of *tert*-butyl esters

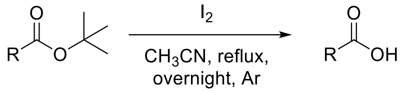

Deprotections of *tert*-butyl esters **2** and **4** – **6** were carried out following a procedure reported in the literature.[15] A mixture of *tert*-butyl ester (1.0 mmol, *c* = 0.1 M) and iodine (30 mol%) in 10 mL acetonitrile was stirred under reflux conditions overnight under an Ar atmosphere (oil bath as heat source). After completed reaction, the mixture was cooled to room temperature, followed by isolation and purification of the product.

5-((2-Hydroxyethoxy)carbonyl)furan-2-carboxylic acid (F_1E_1)

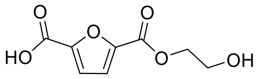

$\mathbf{F_1E_1}$

$\mathbf{F_1E_1}$ was synthesized using the general procedure (0.9 mmol scale). After completed reaction, the solvent was removed under reduced pressure, followed by trituration of the remaining solid with diethyl ether and drying *in vacuo*. The product was obtained as a colorless amorphous solid (121.0 mg, 0.6 mmol, 68%).

^{1}H NMR (400 MHz, DMSO-d_6) δ 7.40 (d, *J* = 3.6 Hz, 1H), 7.33 (d, *J* = 3.7 Hz, 1H), 4.31 – 4.28 (m, 2H), 3.69 – 3.66 (m, 2H). ^{13}C{^{1}H} NMR (101 MHz, DMSO-d_6) δ 158.8, 157.7, 147.4, 145.9, 119.1, 118.5, 66.9, 58.9. HRMS (ESI-TOF) m/z: calcd for $[C_8H_7O_6]^-$ 199.0248; found: 199.0242. M.p. 165.4 – 166.6 °C. The analytical data are in agreement with the previous report, whereby the literature spectra indicate a significant portion of residual MEG.[16]

---

[15] Yadav, J. S.; Balanarsaiah, E.; Raghavendra, S.; Satyanarayana, M. Chemoselective hydrolysis of *tert*-butyl esters in acetonitrile using molecular iodine as a mild and efficient catalyst. *Tetrahedron Lett.* **2006**, *47*, 4921-4924. DOI: 10.1016/j.tetlet.2006.05.011.

[16] Raboni, F.; Oliveri, A.; Rocca, V. M.; Moni, L.; Kumar, V.; Varrone, C.; Pellis, A. On the Environmentally Friendly Synthesis of 2-Hydroxyethyl Furan-5-Carboxylic Acid (MHEF) and Bis(2-Hydroxyethyl) Furan-2,5-Dicarboxylate (BHEF). *Chem. Open* **2025**, e202400507. DOI: 10.1002/open.202400507.

5,5'-((Ethane-1,2-diylbis(oxy))bis(carbonyl))bis(furan-2-carboxylic acid) (F_2E_1)

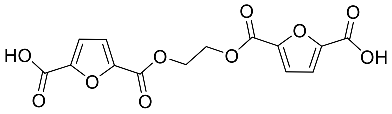

F_2E_1

F_2E_1 was synthesized using the general procedure (1.0 mmol scale). After completed reaction, the product precipitated from the solution. The solid was filtered off, washed with ethyl acetate, and dried *in vacuo*. The product was obtained as a colorless powder (315.0 mg, 0.93 mmol, 93%).

^1H NMR (300 MHz, DMSO-d_6) δ 7.39 (d, J = 3.7 Hz, 2H), 7.31 (d, J = 3.7 Hz, 2H), 4.61 (s, 4H). ^{13}C NMR (75 MHz, DMSO-d_6) δ 158.8, 157.4, 147.6, 145.4, 119.5, 118.5, 63.1. HRMS (ESI-TOF) m/z: calcd for [$C_{14}H_{10}O_{10}Na$] 361.0172; found 361.0178. M.p. 289.3 – 296.1 °C (dec.).

5,5'-(((((Furan-2,5-dicarbonyl)bis(oxy))bis(ethane-2,1-diyl))bis(oxy))bis(carbonyl)) bis(furan-2-carboxylic acid) (F_3E_2)

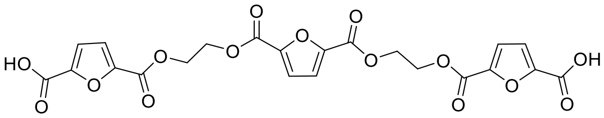

F_3E_2

F_3E_2 was synthesized using the general procedure (2.5 mmol scale). After completed reaction, the product precipitated from the solution. The solid was filtered off, washed with ethyl acetate, and dried *in vacuo*. The product was obtained as a colorless powder (1.25 g, 2.4 mmol, 96%).

^1H NMR (300 MHz, DMSO-d_6) δ 7.42 (s, 2H), 7.38 (d, J = 3.7 Hz, 2H), 7.31 (d, J = 3.7 Hz, 2H), 4.61 (s, 8H). $^{13}C\{^1H\}$ NMR (75 MHz, DMSO-d_6) δ 158.8, 157.3, 157.2, 147.6, 146.0, 145.4, 119.5, 119.5, 118.5, 63.2, 63.0. HRMS (ESI-TOF) m/z: calcd for [$C_{22}H_{16}O_{15}Na$]$^+$ 543.0381; found 543.0388. M.p. 269.5 – 271.1 °C (dec.).

5-((4-Hydroxybutoxy)carbonyl)furan-2-carboxylic acid (F_1B_1)

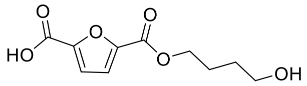

F_1B_1

F_1B_1 was synthesized using the general procedure (1.0 mmol scale). After completed reaction, the solvent was removed under reduced pressure. The remaining solid was dissolved in ethyl acetate, washed with sat. aq. NaS_2O_3 to remove residual iodine, and concentrated under reduced pressure. The crude product was subjected to flash column chromatography on deactivated silica gel for purification (eluent: CH_2Cl_2/MeOH 19:1, vol/vol).[17] The product was obtained as a colorless powder (118.0 mg, 0.52 mmol, 52%).

^1H NMR (300 MHz, DMSO-d_6) δ 7.37 (d, J = 3.6 Hz, 1H), 7.31 (d, J = 3.7 Hz, 1H), 4.29 (t, J = 6.6 Hz, 2H), 3.43 (t, J = 6.3 Hz, 2H), 1.77 – 1.67 (m, 2H), 1.55 – 1.46 (m, 2H). $^{13}C\{^1H\}$ NMR (75 MHz, DMSO- d_6) δ 158.8, 157.6, 147.4, 145.9, 119.0, 118.4, 65.2, 60.2, 28.8, 25.0. HRMS

[17] The column was pre-treated ("deactivated") by applying 2 mL NEt_3 to the column (approx. 50 g silica gel), followed by passing a small amount of eluent mixture.

(ESI-TOF) m/z: calcd for. $[C_{10}H_{11}O_6]^-$ 227.0561; found: 227.0554. M.p. 115.5 – 118.5 °C. Spectral data are in agreement with the literature.[18]

Bis(2-hydroxyethyl) furan-2,5-dicarboxylate (F_1E_2)

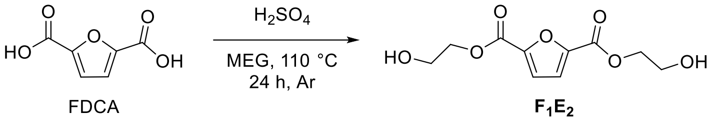

Seven drops of conc. H_2SO_4 was added to a solution of FDCA (1.56.0 g, 10.0 mmol) in monoethylene glycol (MEG, 20 mL). The mixture was then stirred at 110 °C for 24 h under an Ar atmosphere. After completion of the reaction, water (2 x 40 mL) was added, followed by extraction with ethyl acetate (6 x 40 mL). The organic layer was treated with brine, dried over anhydrous Na_2SO_4 and then concentrated under reduced pressure. The crude product was purified using flash column chromatography (eluent: CH_2Cl_2/MeOH 19:1, vol/vol). The desired product was obtained as a colorless powder (2.03 g, 8.31 mmol, 83%).

^{1}H NMR (300 MHz, DMSO-d_6) δ 7.44 (s, 2H), 4.95 (t, J = 5.6 Hz, 2H), 4.32 – 4.29 (m, 4H), 3.71 – 3.65 (m, 4H). ^{13}C{^{1}H} NMR (75 MHz, DMSO-d_6) δ 157.6, 146.2, 119.2, 67.0, 58.9. HRMS (ESI-TOF) m/z: calcd for $[C_{10}H_{12}O_7Na]^+$ 267.0475; found 267.0479. M.p. 80.7 – 83.4 °C. Spectral data are in agreement with the literature.[19]

[18] Parisi, D.; Riley, C.; Srivastava, A. S.; McCue, H. V.; Johnson, J. R.; Carnell, A. J. PET hydrolysing enzymes catalyse bioplastics precursor synthesis under aqueous conditions. *Green Chem.* **2019**, *21*, 3827-3833. DOI: 10.1039/c9gc01284b.

[19] Kim, M.; Su, Y.; Aoshima, T.; Fukuoka, A.; Hensen, E. J. M.; Nakajima, K. Effective Strategy for High-Yield Furan Dicarboxylate Production for Biobased Polyester Applications. *ACS Catal.* **2019**, *9*, 4277–4285. DOI: 10.1021/acscatal.9b00450.

1.6 Preparation of methanolysate model fragments

2-(2-Hydroxyethyl) 5-methyl furan-2,5-dicarboxylate (MeF_1E_1)

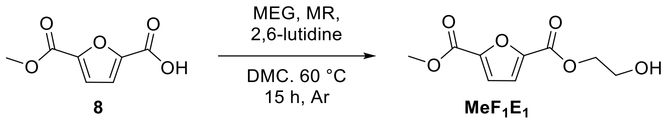

Compound **MeF_1E_1** was synthesized using a modified version of a procedure reported in the literature.[20] A mixture of compound **8** (85.1 mg, 0.5 mmol), MEG (84 µL, 1.5 mmol), and Mukaiyama's reagent (140.5 mg, 0.55 mmol) in 1 mL of dimethylcarbonate (DMC) was prepared, to which 2,6-lutidine (122.0 µL, 1.05 mmol) was added. The resulting mixture was stirred under an Ar atmosphere for 15 h at 60 °C. After completed reaction, the reaction mixture was diluted with 15 mL ethyl acetate and filtered to remove the precipitated 1-methylpyridin-2(1*H*)-one. The filtrate was washed with aq. 1 M HCl (1 x 5 mL) to remove the 2,6-lutidine, dried over anhydrous Na_2SO_4 and concentrated under reduced pressure. The crude product was subjected to flash column chromatography for purification (eluent: CH_2Cl_2/MeOH 97:3, vol/vol). The desired product was obtained as colorless amorphous solid (71 mg, 0.33 mmol, 66%). Diester **$Me_2F_2E_1$** was isolated as a side product in a 16% yield (15 mg, 0.04 mmol).

^{1}H NMR (400 MHz, $CDCl_3$) δ 7.24 (d, J = 3.6 Hz, 1H), 7.19 (d, J = 3.6 Hz, 1H), 4.47 – 4.44 (m, 2H), 3.96 – 3.93 (m, 2H), 3.91 (s, 3H), 2.51 (s, 1H). $^{13}C\{^1H\}$ NMR (101 MHz, $CDCl_3$) δ 158.6, 158.3, 146.8, 146.6, 119.0, 118.6, 67.2, 61.0, 52.6. HRMS (ESI-TOF) m/z: calcd. for $[C_9H_{10}NaO_6]^+$ 237.0370; found 237.0373. M.p. 64.9 – 66.7 °C.

O'^{2},*O*^{2}-(Ethane-1,2-diyl) 5,5'-dimethyl bis(furan-2,5-dicarboxylate) ($Me_2F_2E_1$)

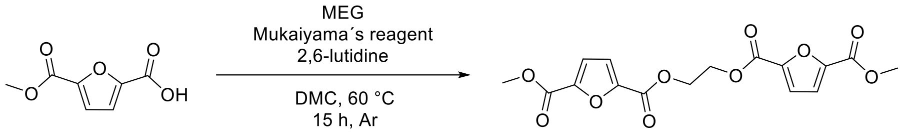

Compound **$Me_2F_2E_1$** was synthesized using a modified version of a procedure reported in the literature.[20] A mixture of compound **8** (106 mg, 0.5 mmol), monoethylene glycol (MEG, 14.0 µL, 0.25 mmol), Mukaiyama's reagent (149 mg, 0.55 mmol), and 1 mL of dimethylcarbonate (DMC) was prepared. Then, 2,6-lutidine (0.12 µL, 1.05 mmol) was added and the resulting mixture was stirred under an Ar atmosphere for 15 h at 60 °C. After completed reaction, the mixture was diluted with CH_2Cl_2 (5 mL) and filtered to remove the precipitated 1-methylpyridin-2(1H)-one. The filtrate was then washed with aq. 1 M HCl (1 x 2 mL), dried over anhydrous Na_2SO_4 and concentrated under reduced pressure. The crude product was subjected to flash column chromatography on deactivated silica (eluent: CH_2Cl_2).[21] The desired product was obtained as colorless solid (63 mg, 0.17 mmol, 69%). Monoesterification product **MeF_1E_1** was isolated as a side product in a 22% yield (12 mg, 0.06 mmol).

---

[20] Jordan, A.; Whymark, K. D.; Sydenham, J.; Sneddon, H. F. A solvent-reagent selection guide for Steglich-type esterification of carboxylic acids. *Green Chem.* **2021**, *23*, 6405-6413. DOI: 10.1039/d1gc02251b.

[21] The column was pre-treated ("deactivated") by applying 2 mL NEt_3 to the column (approx. 50 g silica gel), followed by passing a small amount of eluent mixture.

^{1}H NMR (300 MHz, CD_2Cl_2) δ 7.24 (d, J = 3.7 Hz, 2H), 7.20 (d, J = 3.6 Hz, 2H), 4.65 (s, 4H), 3.91 (s, 6H). $^{13}C\{^{1}H\}$ NMR (75 MHz, CD_2Cl_2) δ 158.7, 158.0, 147.4, 146.6, 119.3, 118.7, 63.7, 52.7. HRMS (ESI-TOF) m/z: calcd. for $[C_{16}H_{14}NaO_{10}]^+$ 389.0479; found 389.0483. M.p. 194.6-196.0 °C.

***O*'²,*O*²-(((Furan-2,5-dicarbonyl)bis(oxy))bis(ethane-2,1-diyl)) 5,5'-dimethyl bis(furan-2,5-dicarboxylate) ($Me_2F_3E_2$)**

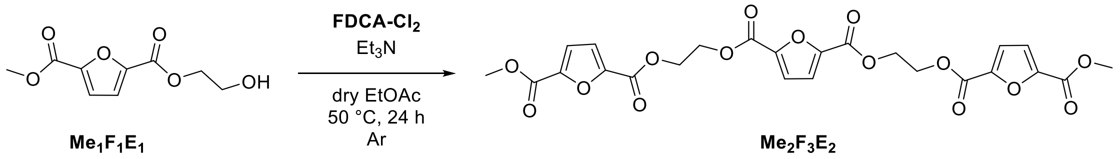

Compound **$Me_2F_3E_2$** was synthesized using a modified version of a procedure reported in the literature. [22] A mixture of compound **$Me_1F_1E_1$** (321.3 mg, 1.5 mmol) and furan-2,5-dicarbonyl dichloride (144.7 mg, 0.75 mmol) in 8 mL of dry ethyl acetate[23] was prepared. Then, triethylamine (523 µL, 5.0 mmol) was added dropwise to the reaction mixture, followed by stirring under an Ar atmosphere for 24 h at 50 °C. The mixture was diluted with 5 mL ethyl acetate and washed with aq. 1 M HCl (1 x 3 mL), brine (1 x 3 mL), and dried over anhydrous Na_2SO_4. After removal of the solvent under reduced pressure, the crude product was subjected to flash column chromatography for purification (eluent: CH_2Cl_2/MeOH 19:1, vol/vol). The desired product was obtained as colorless amorphous solid (367 mg, 0.67 mmol, 90%).

^{1}H NMR (400 MHz, CD_2Cl_2) δ 7.24 (s, 2H), 7.23 (d, J = 3.6 Hz, 2H), 7.20 (d, J = 3.6 Hz, 2H), 4.65 (s, 8H), 3.91 (s, 6H). $^{13}C\{^{1}H\}$ NMR (101 MHz, CD_2Cl_2) δ 158.7, 158.0, 158.0, 147.4, 147.0, 146.6, 119.3, 118.7, 63.4, 63.3, 52.7. HRMS (ESI-TOF) m/z: calcd. for $[C_{24}H_{20}NaO_{15}]^+$ 571.0694; found 571.0704. M.p. 181.6-183.4 °C.

[22] Pham, P. H.; Barlow, S.; Marder, S. R.; Luca, O. R. Electricity-driven recycling of ester plastics using one-electron electro-organocatalysis. *Chem. Catal.* **2023**, *3*, 100675. DOI: 10.1016/j.checat.2023.100675.

[23] Dry ethyl acetate was purchased and used as received (AcroSeal™, 99.9%, *extra dry*, over molecular sieve).

1.7 Isolation and characterization of *N*-Acylurea compound 3

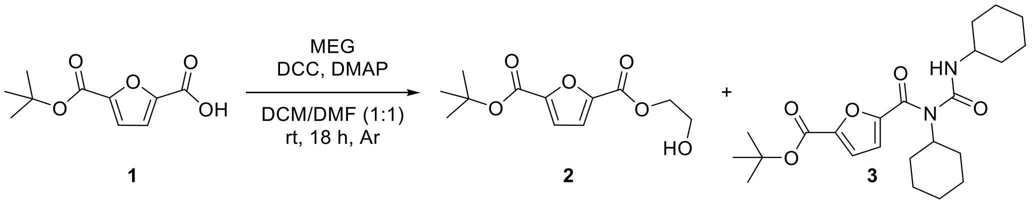

A mixture of **1** (212 mg, 1.0 mmol), monoethylene glycol (MEG, 167 µL, 3.0 mmol), *N,N'*-dicyclohexylcarbodiimide (DCC, 227.0 mg, 1.1 mmol), 4-dimethylaminopyridine (DMAP, 12.2 mg, 0.1 mmol) and 6 mL of DCM/DMF (1:1, vol/vol) was stirred under an Ar atmosphere for 18 h at room temperature. After completed reaction, the mixture was diluted with ethyl acetate (15 mL) and filtered to remove the precipitated 1,3-dicyclohexylurea. The filtrate was then concentrated under reduced pressure. The crude product was subjected to flash column chromatography for purification (eluent: *n*-heptane/ethyl acetate 4:1, vol/vol). Product **2** was obtained as a colorless amorphous solid in 53% yield (136 mg, 0.53 mmol). *N*-acylurea compound **3** was isolated as a side product in 15% yield (63 mg, 0.15 mmol).

^{1}H NMR (300 MHz, $CDCl_3$) δ 7.07 (d, *J* = 3.6 Hz, 1H), 7.04 (d, *J* = 3.6 Hz, 1H), 6.66 (d, *J* = 7.7 Hz, 1H), 4.02 – 4.13 (m, 1H), 3.73 – 3.63 (m, 1H), 2.02 – 1.77 (m, 8H), 1.72 – 1.59 (m, 3H), 1.56 (s, 9H), 1.40 – 1.08 (m, 9H). ^{13}C{^{1}H} NMR (75 MHz, $CDCl_3$) δ 159.6, 157.3, 153.3, 149.5, 147.0, 117.7, 117.6, 82.9, 57.5, 50.1, 32.7, 31.1, 28.3, 26.4, 25.6, 25.4, 24.8. HRMS (ESI-TOF) m/z: calcd. for $[C_{23}H_{34}N_2NaO_5]^+$ 441.2360; found 441.2364. M.p. 142.7 – 144.4 °C.

The molecular structure was also confirmed using X-ray single crystal analysis (see section 2).

1.8 Solubility studies

The analysis of the solubility of model hydrolysates and methanolysates was carried out as follows: A defined amount of the respective compound (0.025 mmol) was placed in a glass vial, followed by dropwise addition of the solvent using a micropipette or a syringe under stirring until the solid was dissolved completely or until the added total volume of solvent reached 20 mL.

Table S5. Summary of the concentrations of the model compounds achieved in the respective solvents.

Species	Concentration achieved (mM) [a]								
	CH_2Cl_2	$CHCl_3$	EtOAc	DMSO	MeOH	CH_3CN	HFIP [b]	H_2O	aq. buffer [c]
**F_1E_2**	20	-	50	1000	333	250	50	27	*d*
**F_1E_1**	*d*	-	28	1250	450	70	500	70	500
**F_2E_1**	-	-	-	125	*d*	*d*	1.4	*d*	114
**F_3E_2**	-	-	-	100	*d*	*d*	5.9	*d*	2.4
**$Me_2F_2E_1$**	18	9.6	-	*d*	*d*	2.3	*d*	-	-
**$Me_2F_3E_2$**	20.8	9.6	-	2.1	*d*	2.3	625	-	-

In case of blank entries, solubilities were not tested. [a] Apparent concentrations calculated from the amount of substance and the volume of added solvent. [b] HFIP = 1,1,1,3,3,3-hexafluoro-2-propanol. [c] Aqueous 1.0 M K_2HPO_4/KH_2PO_4 buffer (pH 7.5). [d] Incomplete dissolution in 20 mL solvent (corresponds to a solubility <1.25 mM).

2. Supplementary data

2.1 X-ray crystal structure analysis of compounds 3 and 5

Diffraction data were collected on a STOE IPDS II diffractometer. The structures were solved by intrinsic phasing (SHELXT: Sheldrick, G. M. *Acta Cryst.* **2015**, *A71*, 3.) and refined by full-matrix least-squares procedures on F^2 (SHELXL: Sheldrick, G. M. *Acta Cryst.* **2015**, *C71*, 3.). XP (Bruker AXS) was used for graphical representations. CCDC 2486708 and 2486709 contain the supplementary crystallographic data for this paper. These data are provided free of charge by the joint Cambridge Crystallographic Data Centre and Fachinformationszentrum Karlsruhe Access Structures service www.ccdc.cam.ac.uk/structures.

Preparation of single crystals of compound **3**: A saturated solution of 20 mg of compound **3** was prepared using a 4:1 (vol/vol) mixture of *n*-hexane and ethyl acetate along with three to four drops of CH_2Cl_2. The mixture was kept in the fume hood for more than a week for slow evaporation of the solvents under atmospheric conditions.

Crystal data of compound **3**: $C_{23}H_{34}N_2O_5$, M = 418.52, triclinic, space group $P\bar{1}$, a = 10.0655(3), b = 13.6827(4), c = 18.4520(5) Å, α = 96.097(2), β = 105.433(2), γ = 108.788(2)°, V = 2267.25(12) Å^3, T = 150(2) K, Z = 4, 37883 reflections measured, 11491 independent reflections (R_{int} = 0.0284), final R values ($I > 2\sigma(I)$): R_1 = 0.0555, wR_2 = 0.1652, final R values (all data): R_1 = 0.0658, wR_2 = 0.1739, 193 parameters, CCDC 2486709.

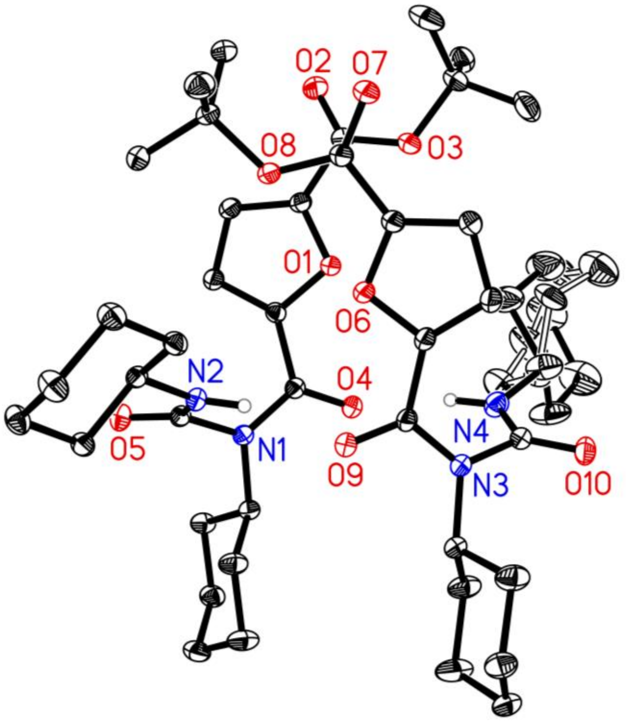

Figure S1. Molecular structure of compound **3**. Displacement ellipsoids correspond to 30% probability. One cyclohexyl ring of one of the two molecules of the asymmetric unit is disordered over two sites with occupancies of 0.573(4): 0.427(4). The lower occupied unit is shown with unfilled lines. C-bound hydrogen atoms are omitted for clarity.

Preparation of single crystals of compound **5**: A saturated solution of 20 mg of pure **5** in CH_2Cl_2 was left for slow evaporation of the solvent under atmospheric conditions in the fume hood for 48 hours.

Crystal data of compound **5**: $C_{22}H_{26}O_{10}$, M = 450.43, triclinic, space group $P\bar{1}$, a = 5.9185(5), b = 9.0017(7), c = 11.7529(9) Å, α = 104.850(6), β = 94.742(6), γ = 108.155(6)°, V = 566.03(8) Å^3, T = 150(2) K, Z = 1, 8929 reflections measured, 2865 independent reflections (R_{int} = 0.0485), final R values ($I > 2\sigma(I)$): R_1 = 0.0522, wR_2 = 0.1421, final R values (all data): R_1 = 0.0635, wR_2 = 0.1536, 148 parameters, CCDC 2486708.

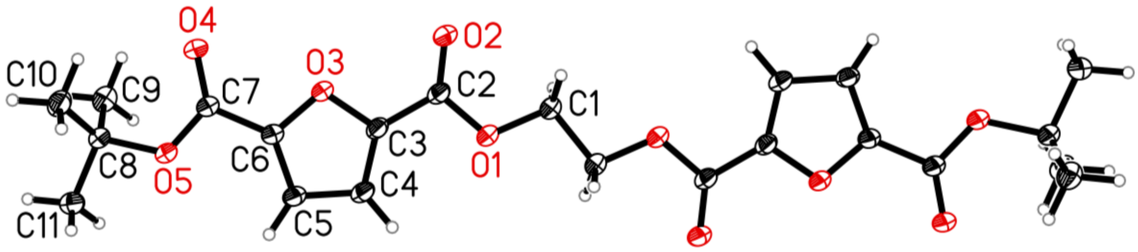

Figure 2. Molecular structure of compound **5**. Displacement ellipsoids correspond to 30% probability. Symmetry-equivalent atoms (unlabeled atoms) are generated by operator -x+2,y+2, -z+2.

2.2 Comparison between NMR spectra of PEF model hydrolysates

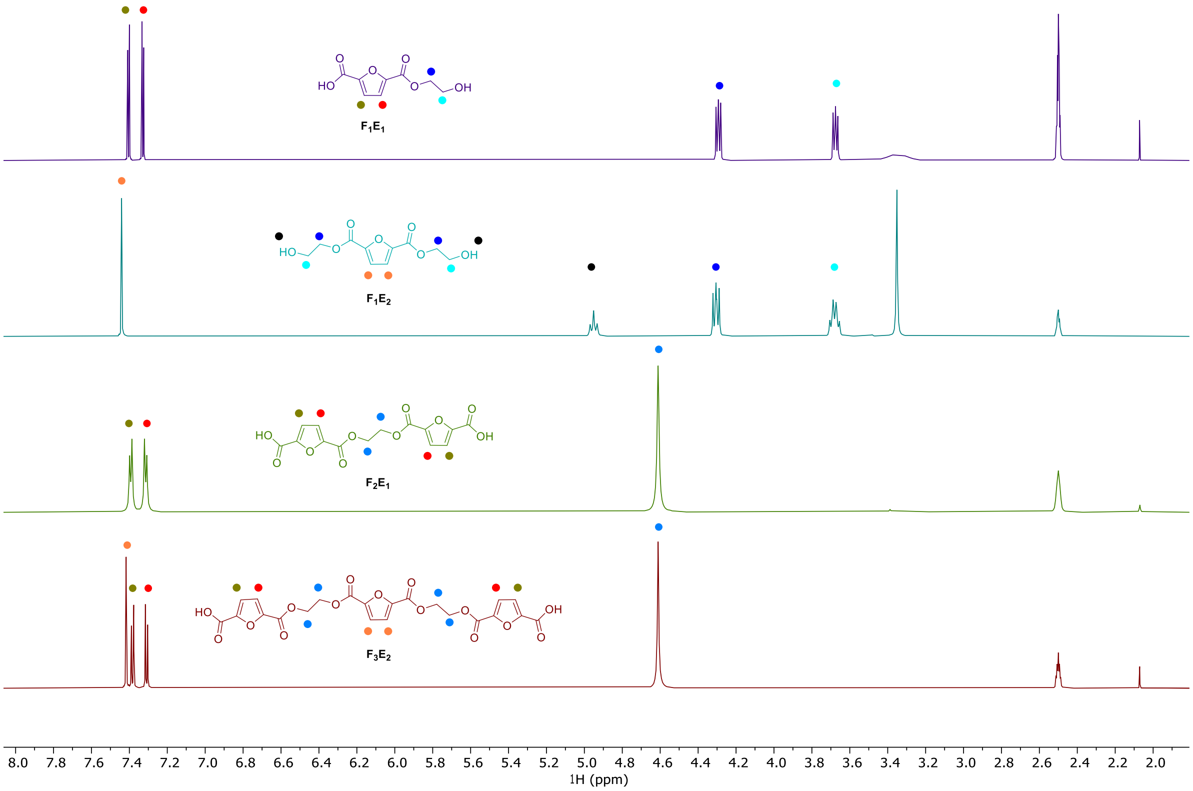

Figure S3. Comparison between the ^{1}H NMR spectra of model hydrolysates **F_1E_1**, **F_1E_2**, **F_2E_1**, and **F_3E_2** (all spectra recorded in DMSO-d_6).

Table S6. Summary of ^{1}H NMR chemical shifts (ppm) of model hydrolysates **F_1E_1**, **F_1E_2**, **F_2E_1**, and **F_3E_2** (all spectra recorded in DMSO-d_6).

Compound	F_1E_1	F_1E_2	F_2E_1	F_3E_2
δ (ppm) aromatic regime	7.40 (d, J = 3.6 Hz, 1H)<br>7.33 (d, J = 3.7 Hz, 1H)	7.44 (s, 2H)	7.39 (d, J = 3.7 Hz, 2H)<br>7.31(d, J = 3.7 Hz, 2H)	7.42 (s, 2H)<br>7.38 (d, J = 3.7 Hz, 2H)<br>7.31 (d, J = 3.7 Hz, 2)
δ (ppm) aliphatic OH		4.95 (t, J = 5.6 Hz, 2H)		
δ (ppm) CH_2 units	4.31 – 4.28 (m, 2H)<br>3.69 – 3.66 (m, 2H)	4.32 – 4.29 (m, 4H)<br>3.71 – 3.65 (m, 4H)	4.61(s, 4H)	4.61 (s, 8H)

Table S7. Summary of ^{13}C NMR chemical shifts (ppm) of model hydrolysates **F_1E_1**, **F_1E_2**, **F_2E_1**, and **F_3E_2** (all spectra recorded in DMSO-d_6).

**Compound**	**F_1E_1**	**F_1E_2**	**F_2E_1**	**F_3E_2**
δ (ppm) $C_{carbonyl}$	158.8, 157.7	157.6	158.8, 157.4	158.8, 157.3, 157.2
δ (ppm) $C_{aromatic}$	147.4 (C2), 145.9 (C5)	146.2 (C2, C5)	147.6 (C2), 145.4 (C5)	147.6 (C2, C2''), 146.0 (C5, C5''), 145.4 (C2', C5')
δ (ppm) $C_{aromatic}$	119.1 (C3), 118.5 (C4)	119.2 (C3, C4)	119.5 (C3), 118.5 (C4)	119.5 (C3, C3''), 119.5 (C4, C4''), 118.5 (C3', C4')
δ (ppm) $C_{methylene}$	66.9, 58.9	67.0, 58.9	63.1	63.2, 63.0

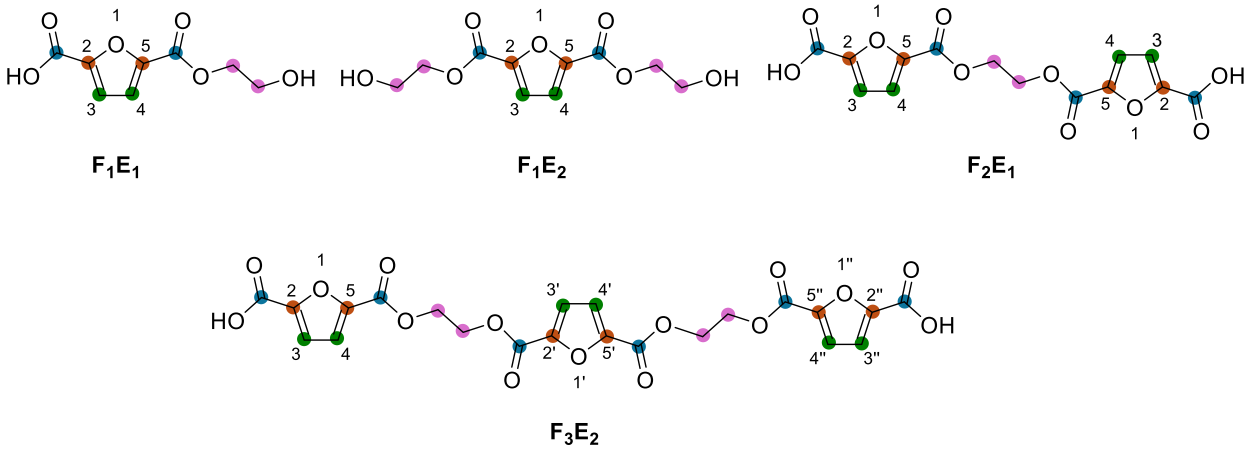

2.3 NMR spectra of synthesized compounds

Furan-2,5-dicarbonyl dichloride. ^{1}H NMR spectrum (400 MHz, $CDCl_3$).

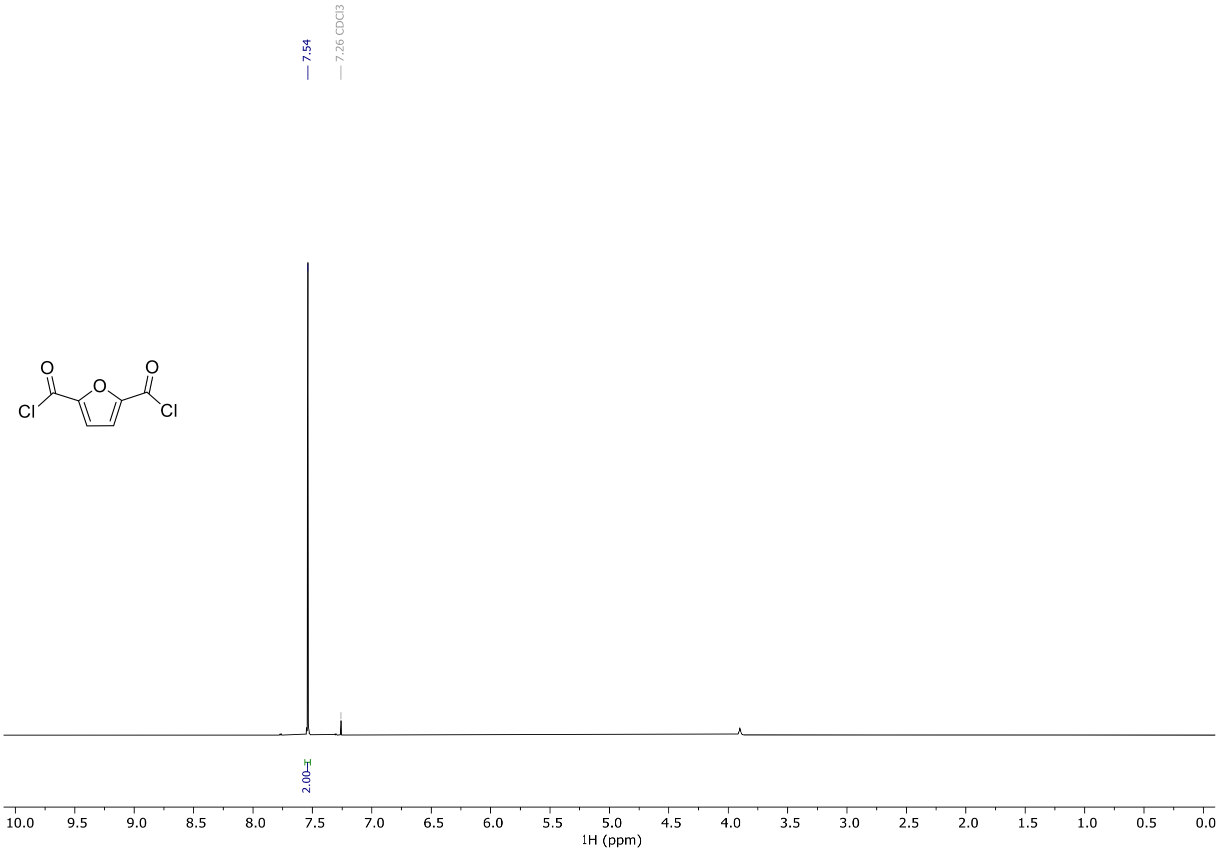

Furan-2,5-dicarbonyl dichloride, $^{13}C\{^{1}H\}$ NMR spectrum (101 MHz, $CDCl_3$).

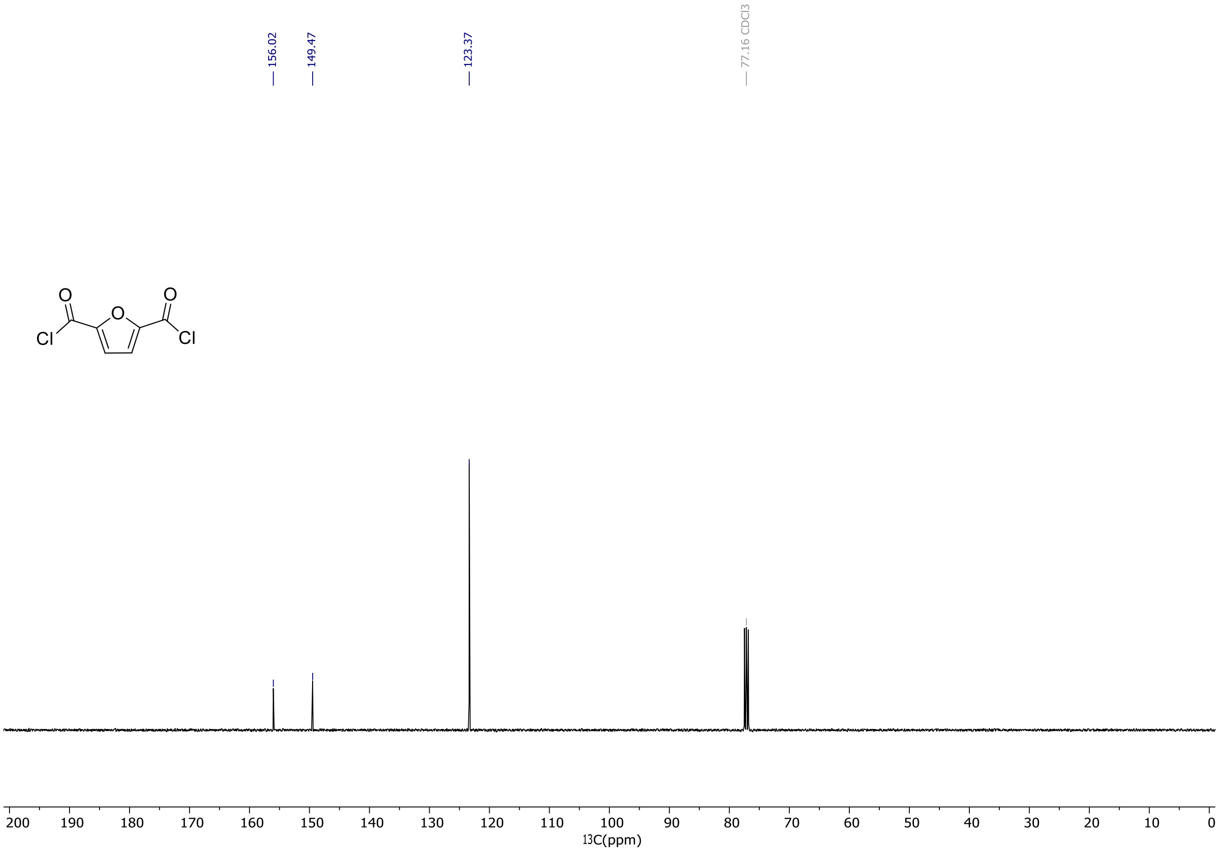

Di-*tert*-butyl 2,5-furandicarboxylate, ^{1}H NMR spectrum (300 MHz, $CDCl_3$).

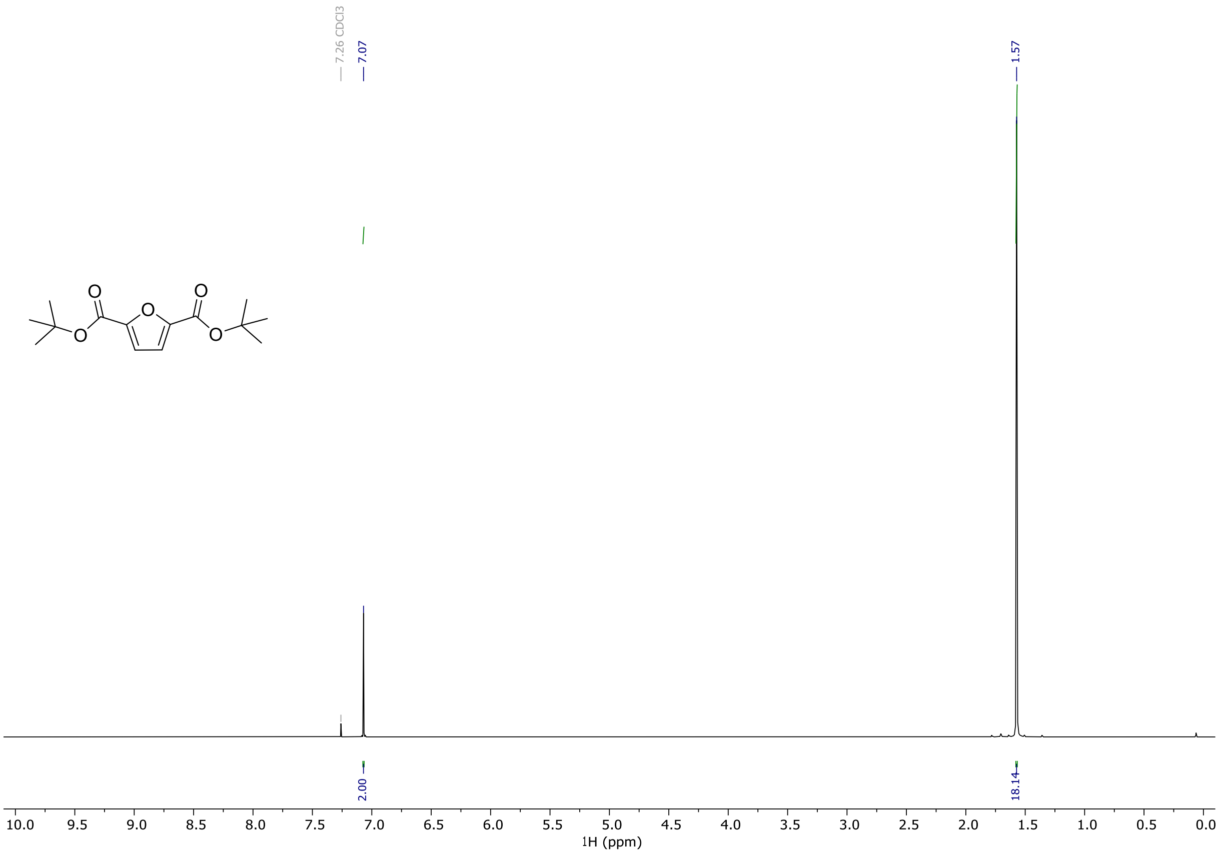

Di-*tert*-butyl 2,5-furandicarboxylate, $^{13}C\{^{1}H\}$ NMR spectrum (75 MHz, $CDCl_3$).

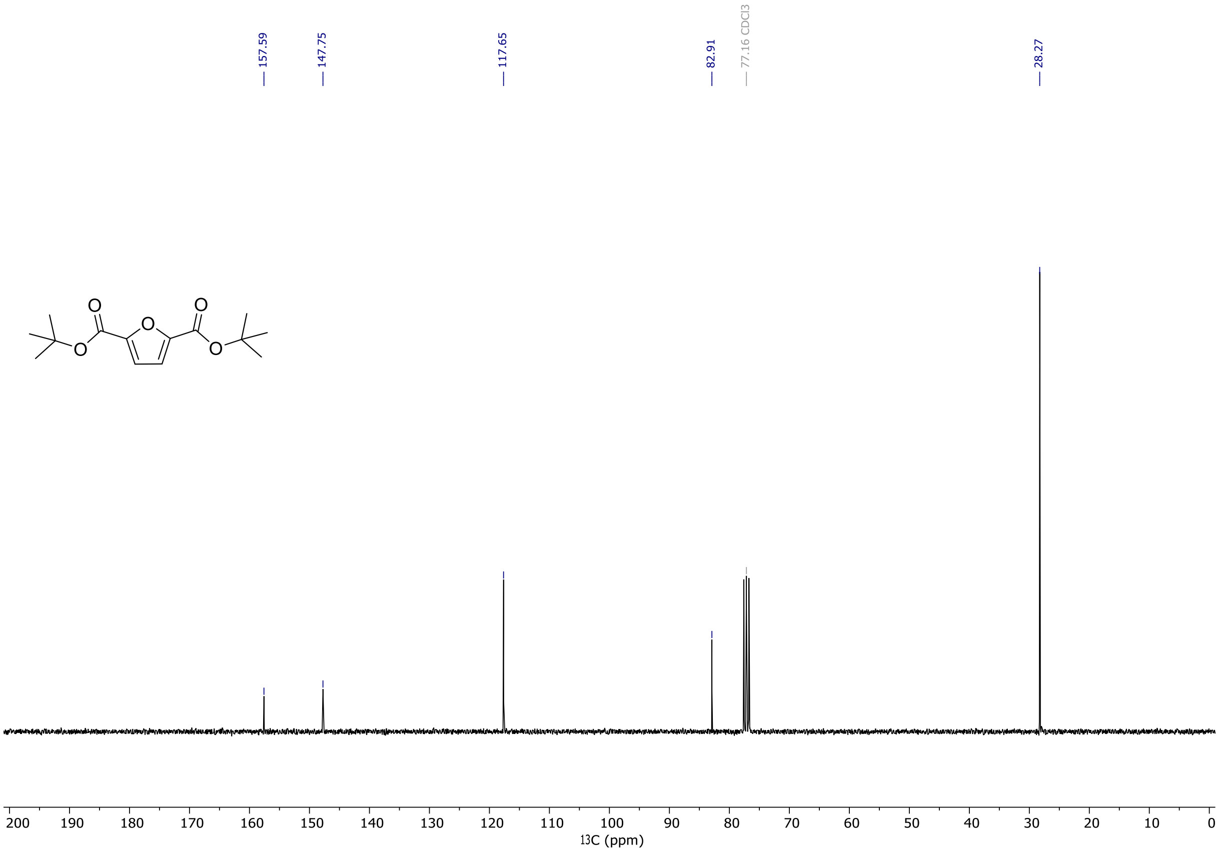

Compound **1**, ^{1}H NMR spectrum (400 MHz, DMSO-d_6).

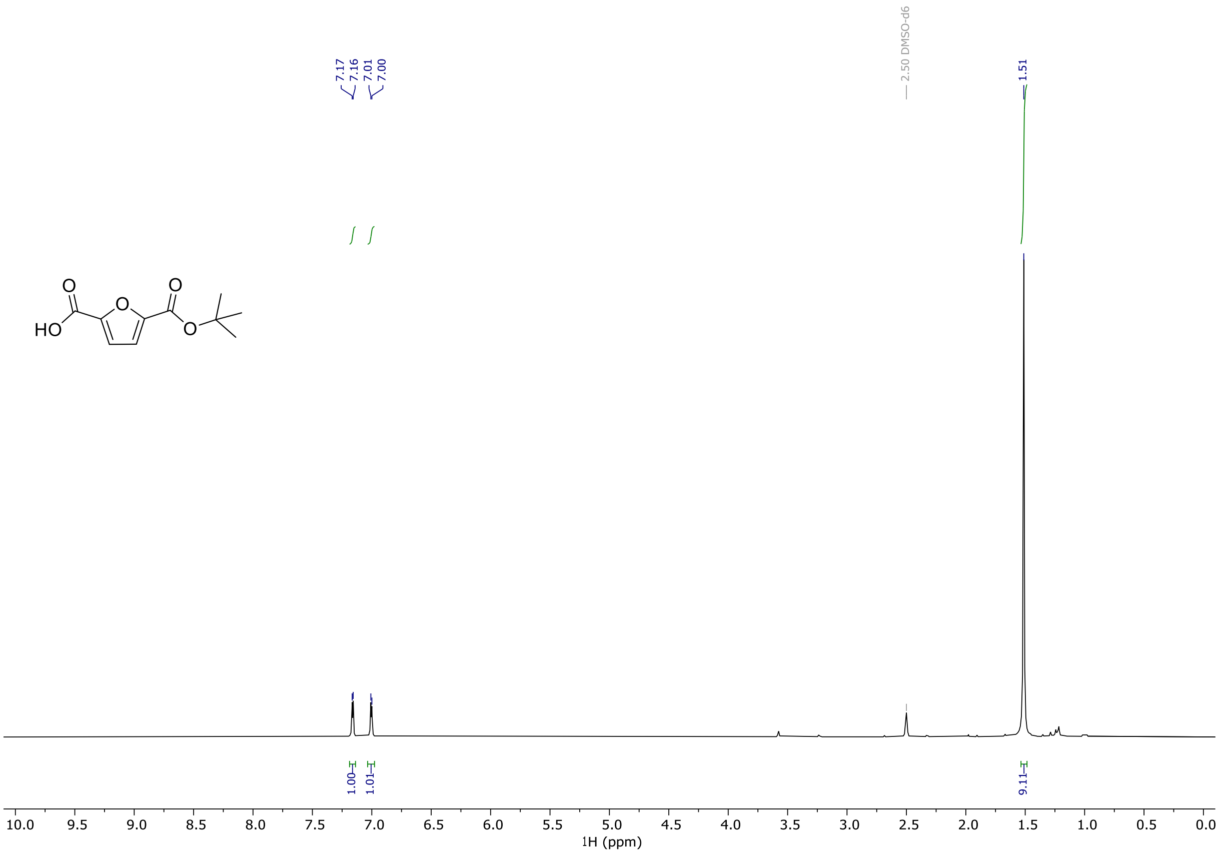

Compound **1**, $^{13}C\{^{1}H\}$ NMR spectrum (101 MHz, DMSO-d_6).

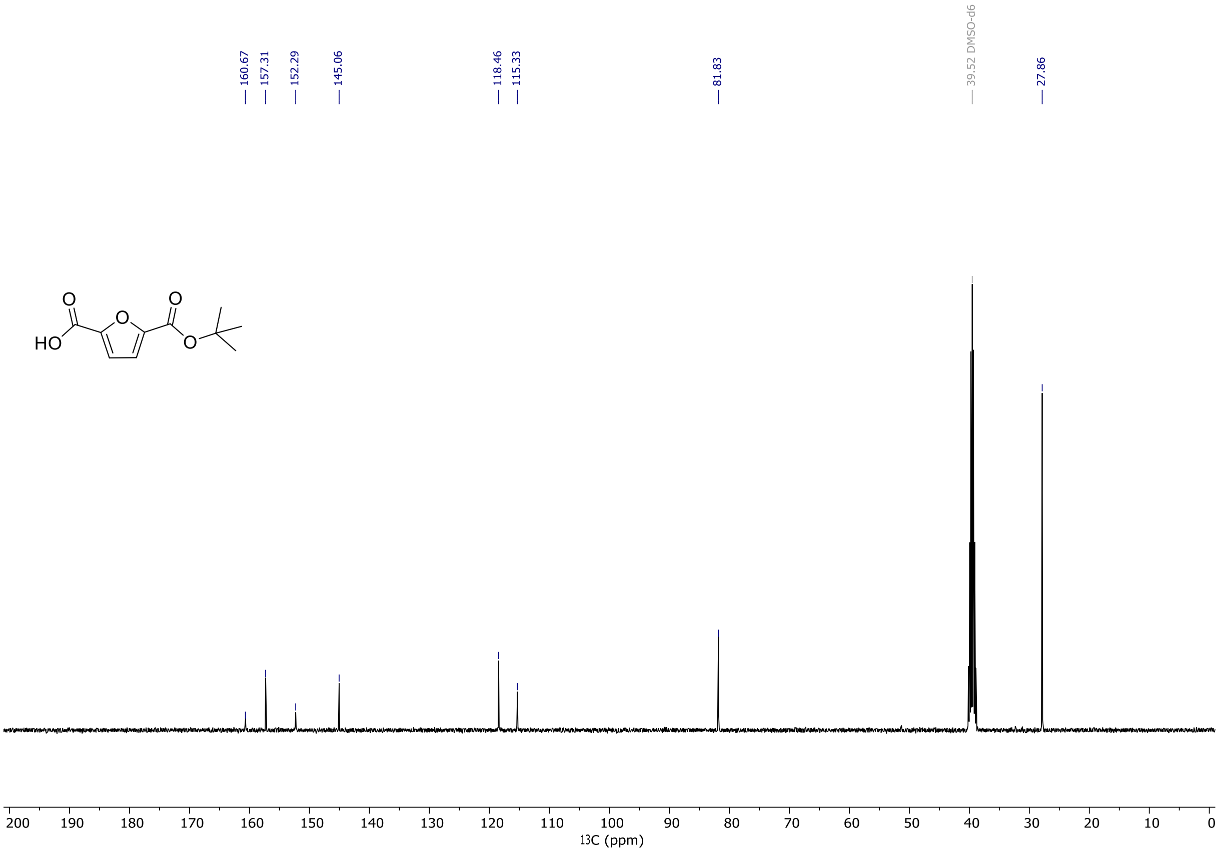

Compound **8**, ^{1}H NMR spectrum (300 MHz, DMSO-d_6).

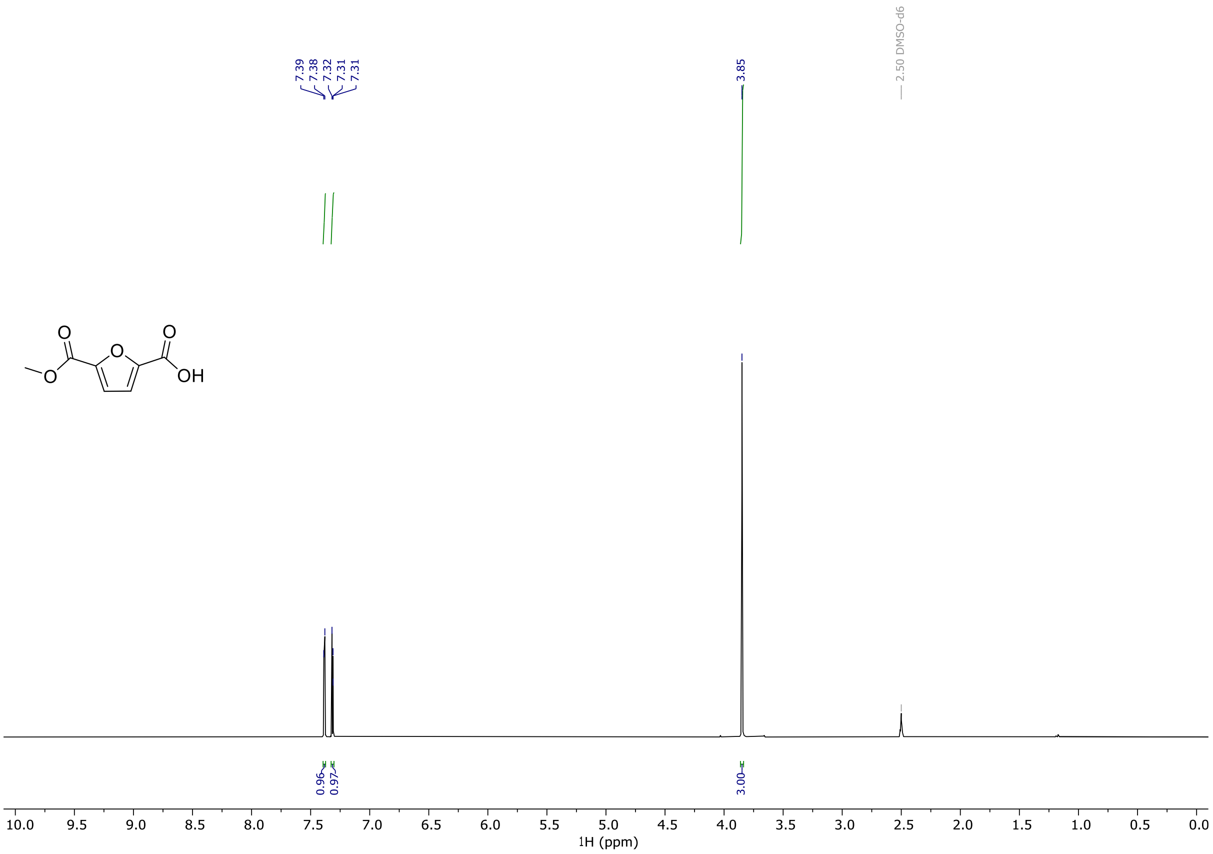

Compound **8**, $^{13}C\{^{1}H\}$ NMR spectrum (75 MHz, DMSO-d_6).

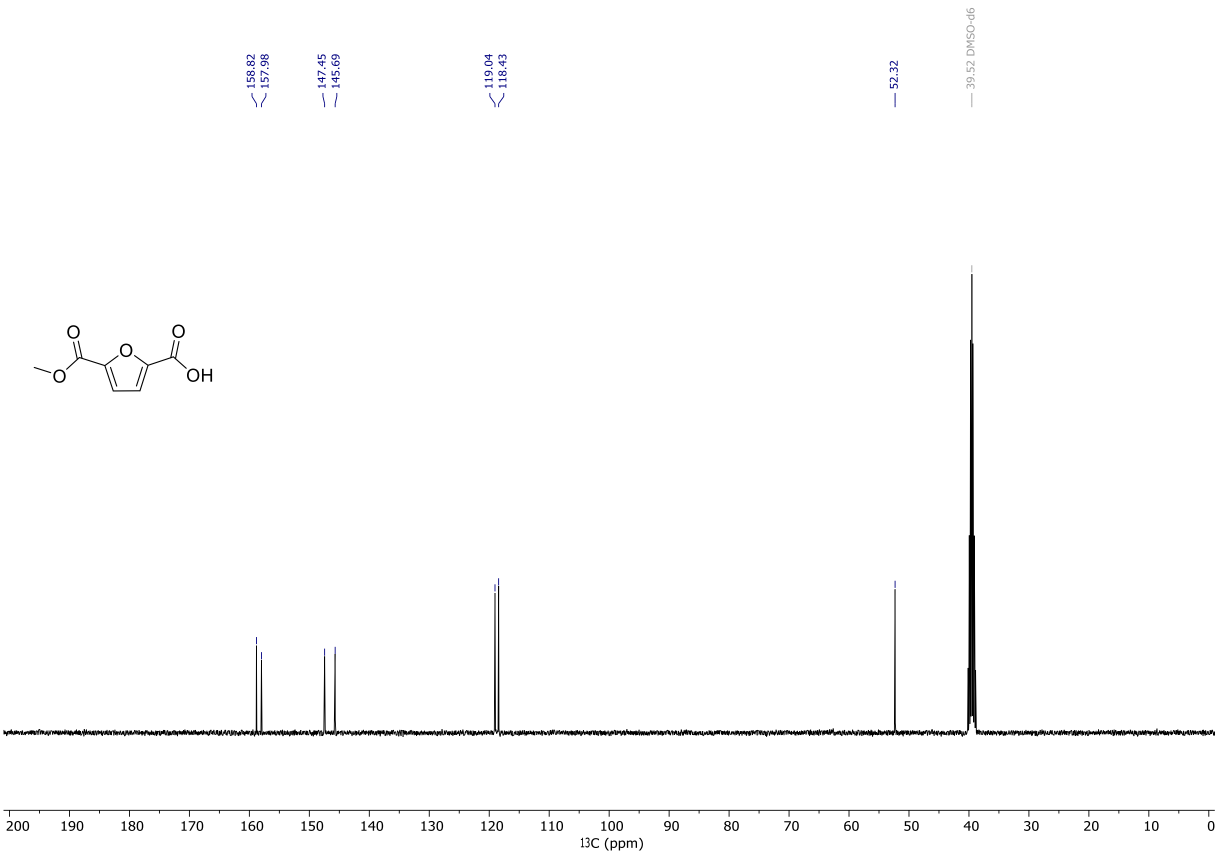

Compound **2**, ^{1}H NMR spectrum (400 MHz,$CDCl_3$).

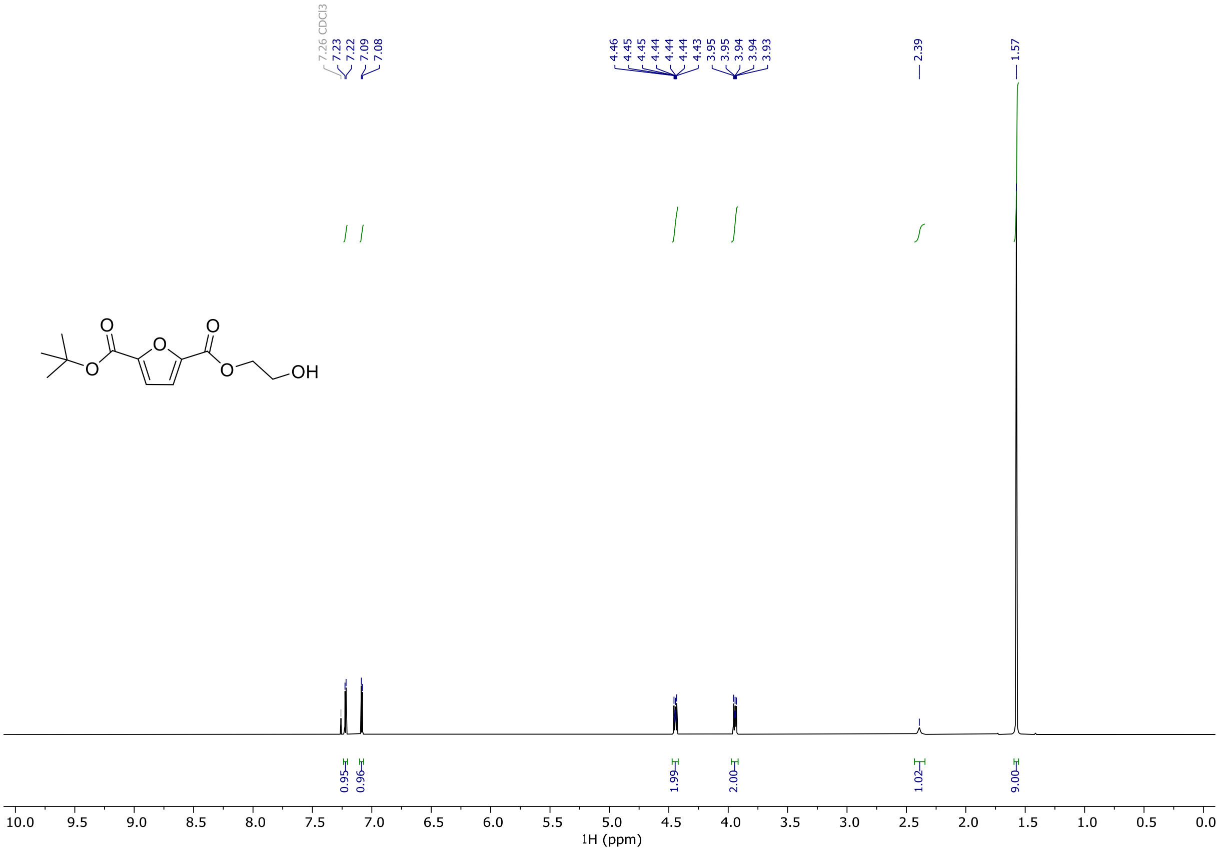

Compound **2**, ^{13}C{^{1}H} NMR spectrum (101 MHz, $CDCl_3$).

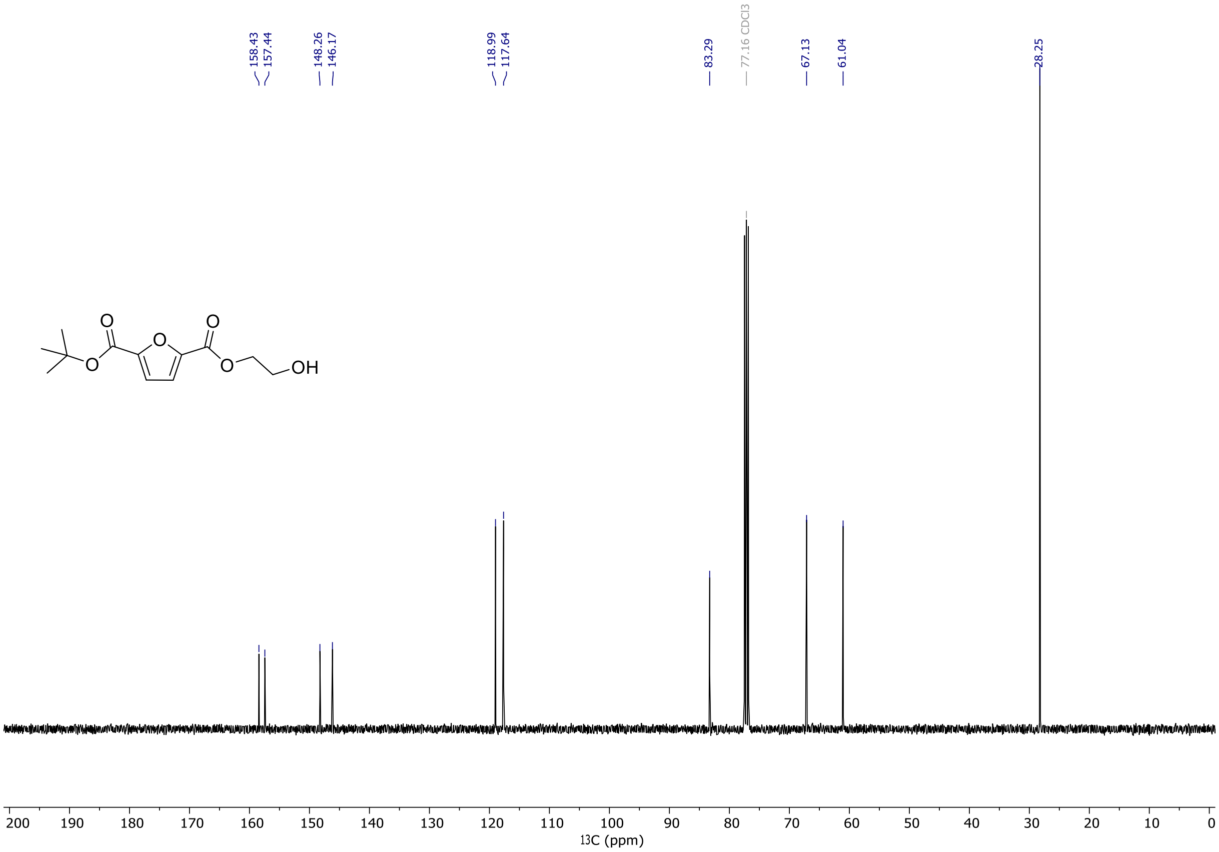

Compound **5**, ^{1}H NMR spectrum (400 MHz, $CDCl_3$).

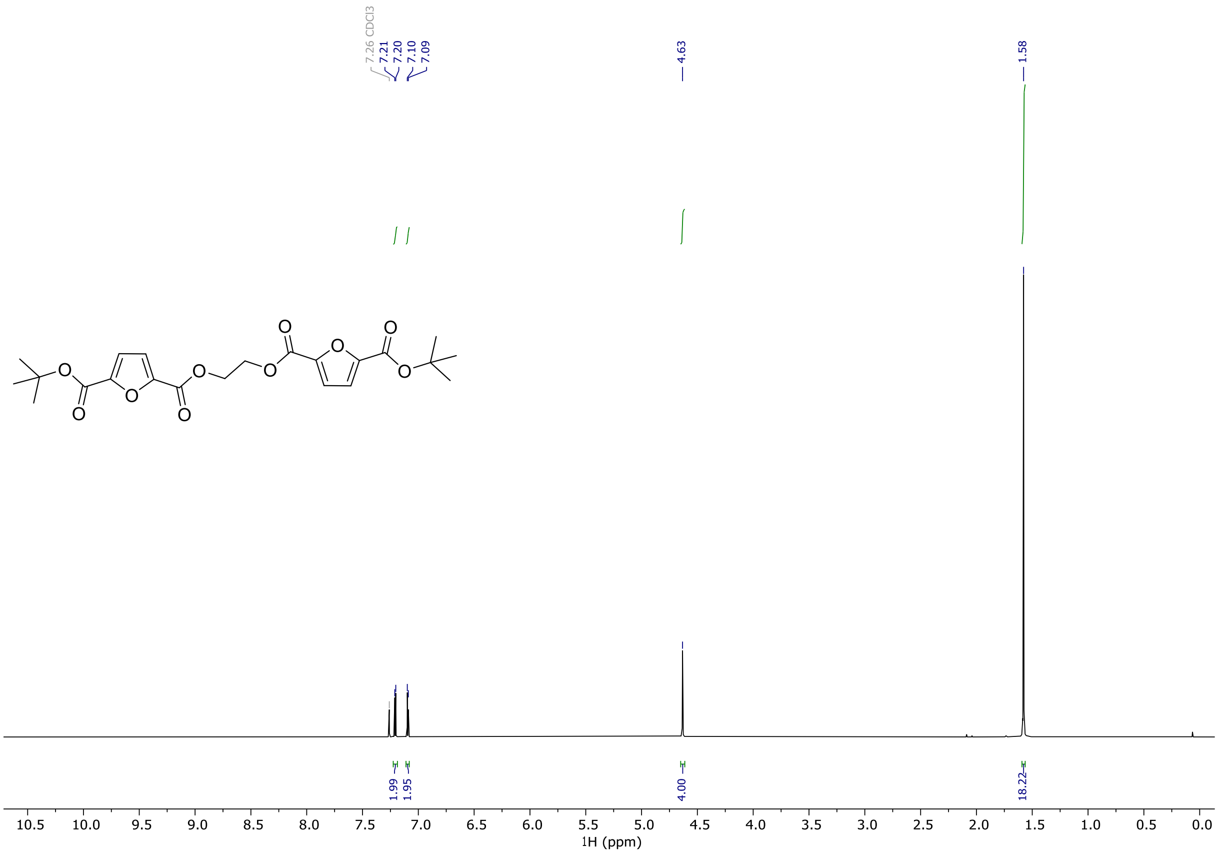

Compound **5**, $^{13}C\{^{1}H\}$ NMR spectrum (101 MHz, $CDCl_3$).

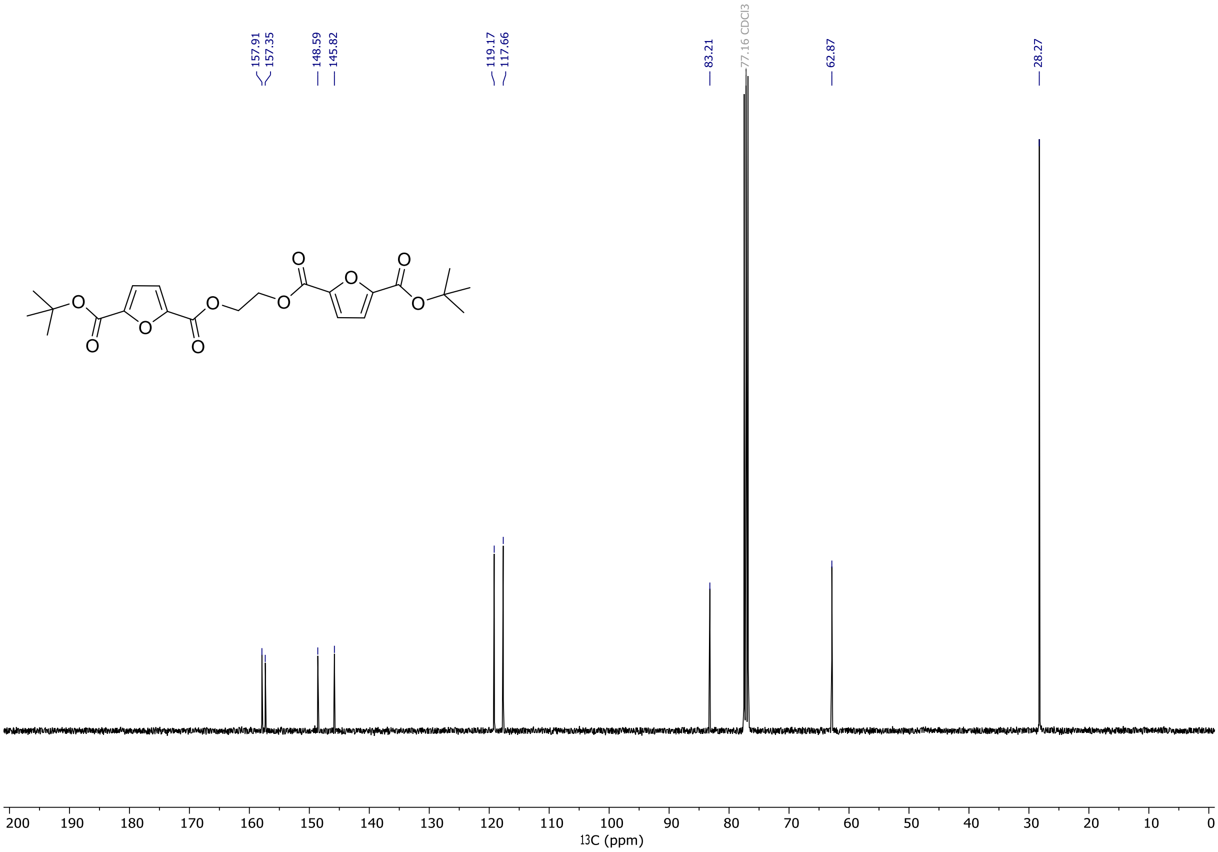

Compound **6**, ^{1}H NMR spectrum (300 MHz, $CDCl_3$).

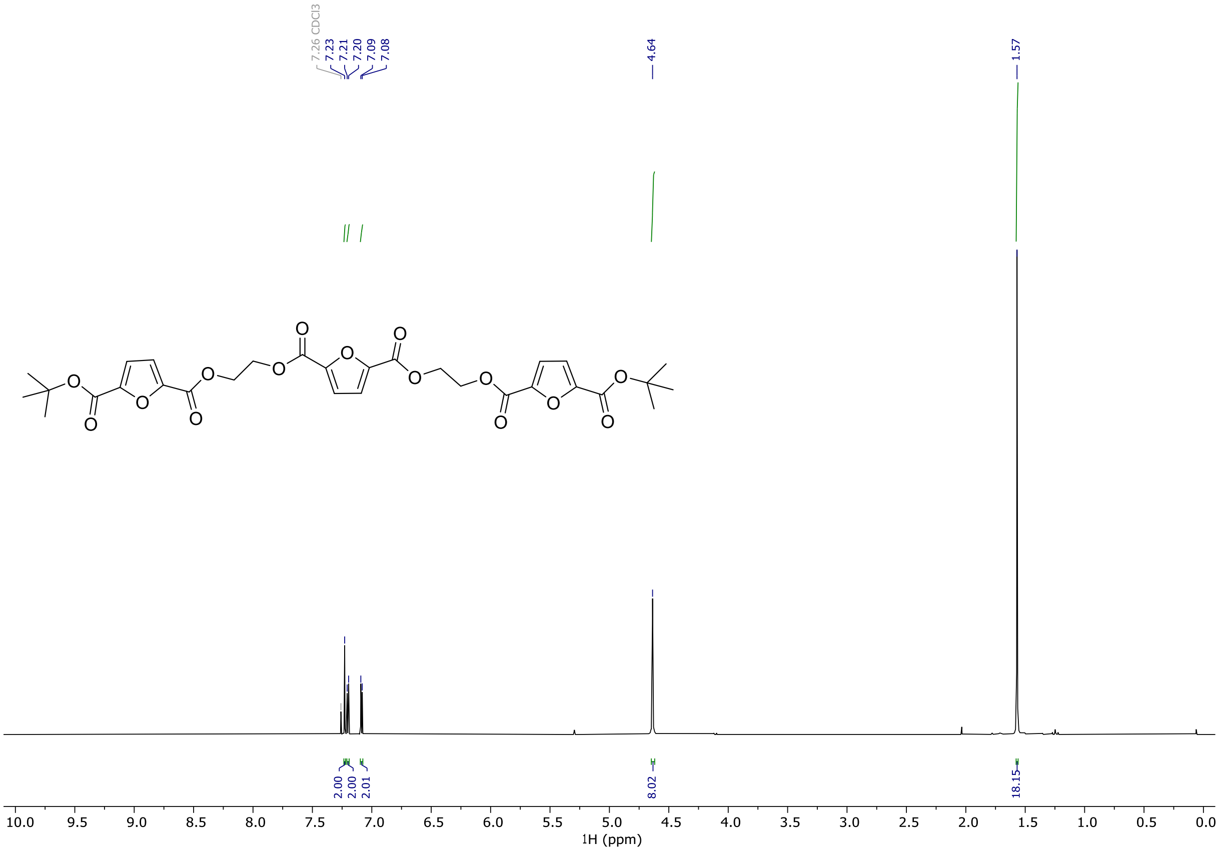

Compound **6**, $^{13}C\{^{1}H\}$ NMR spectrum (75 MHz, $CDCl_3$).

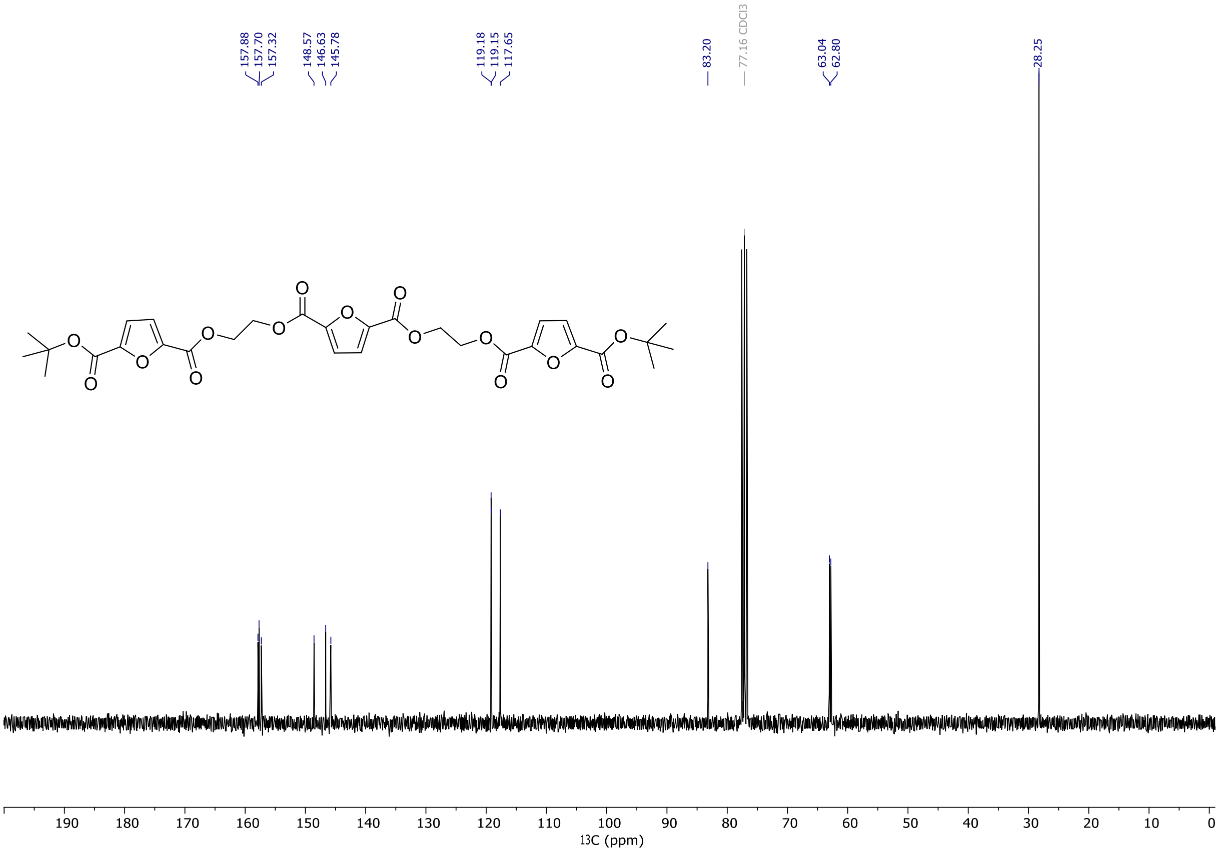

Compound **4**, ^{1}H NMR spectrum (400 MHz, $CDCl_3$).

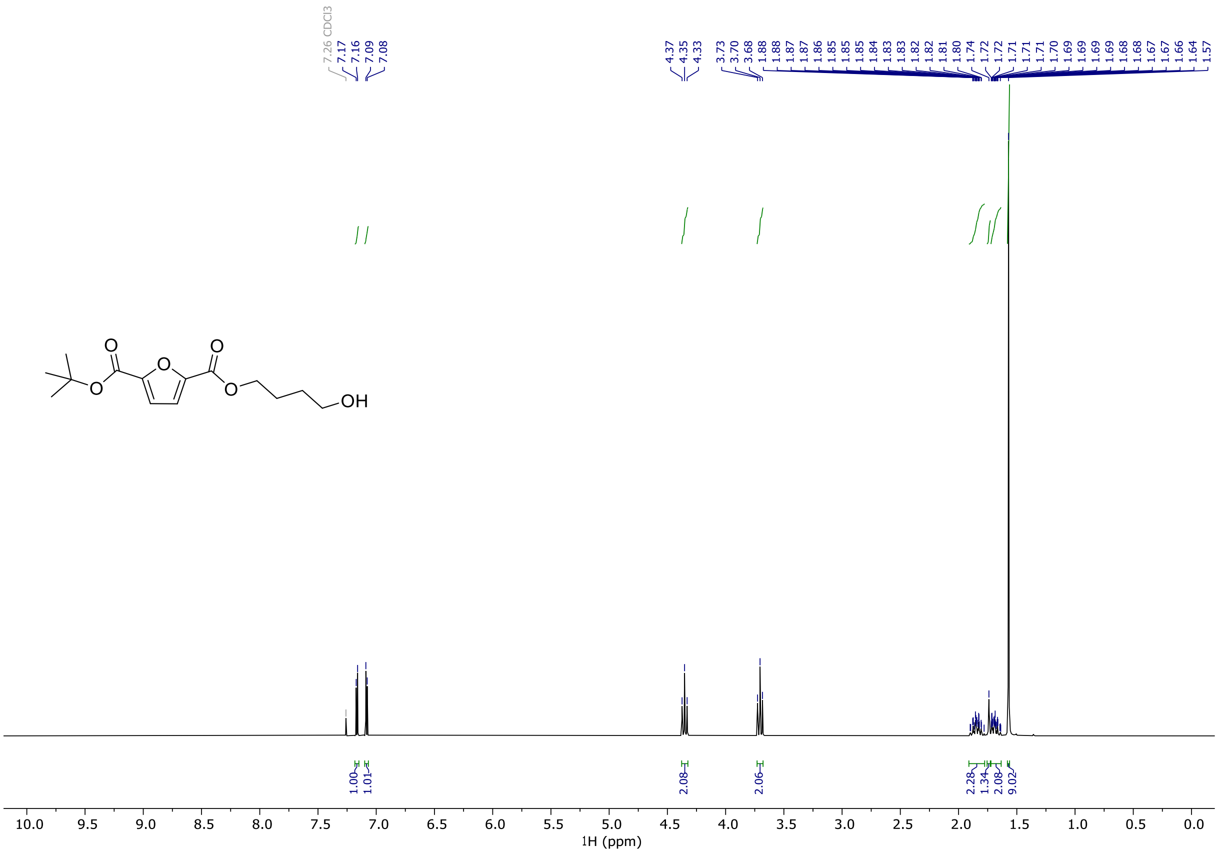

Compound **4**, ^{13}C{^{1}H} NMR spectrum (75 MHz, $CDCl_3$).

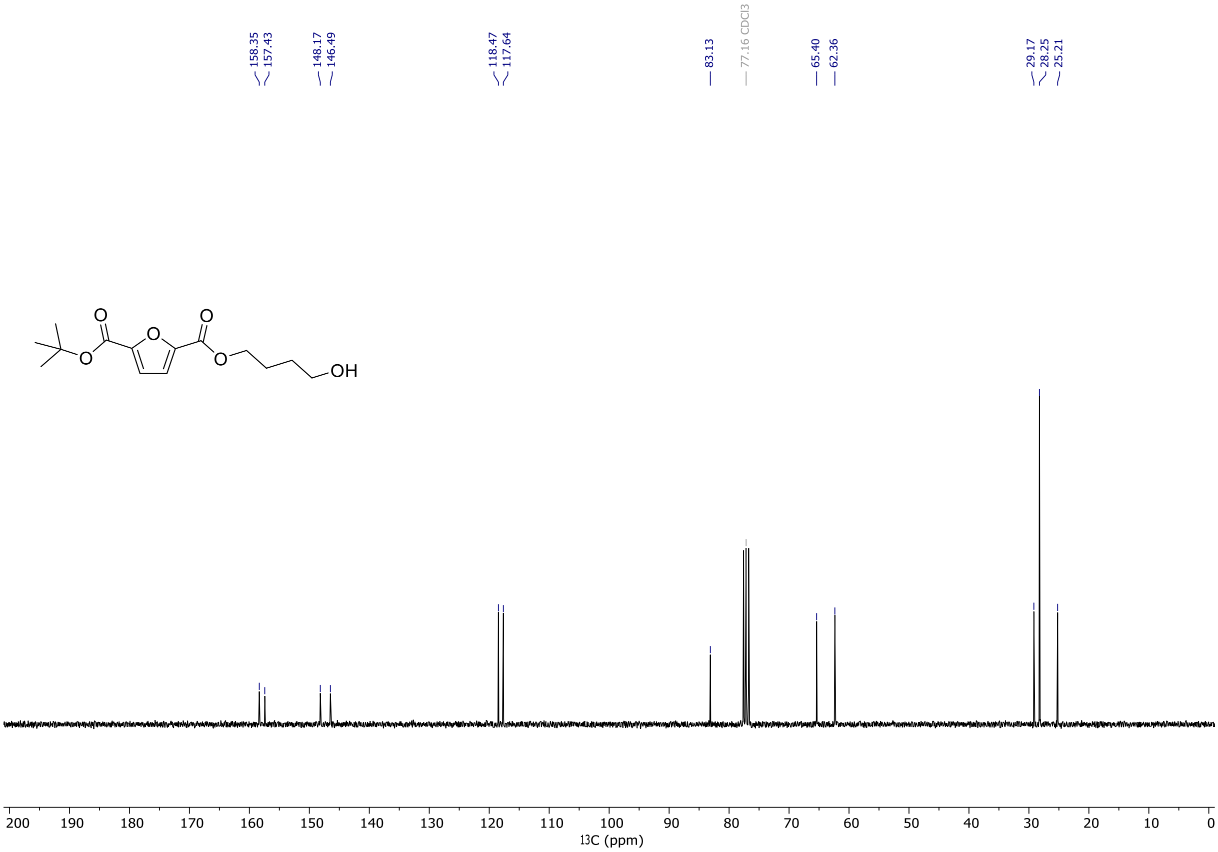

Compound **F$_1$E$_1$**, ^{1}H NMR spectrum (400 MHz, DMSO-d_6).

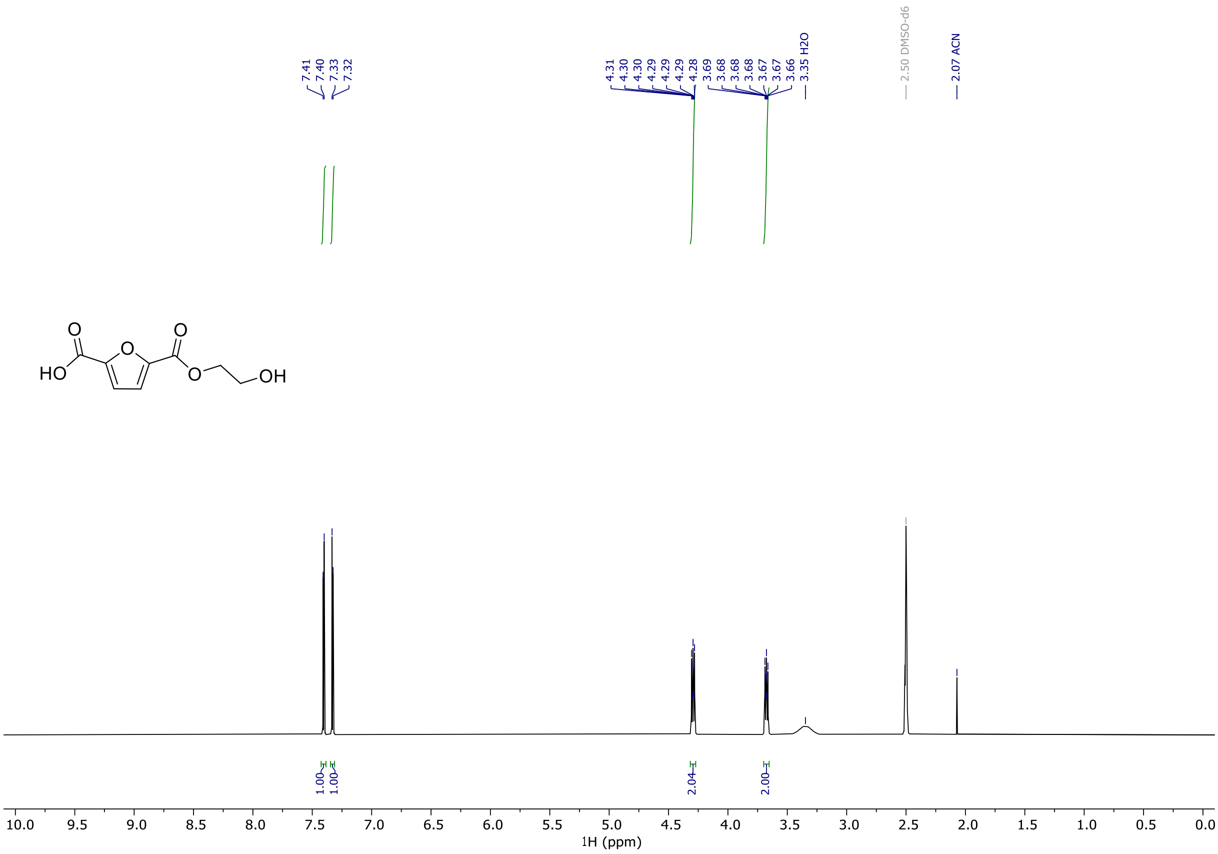

Compound **F$_1$E$_1$**, ^{13}C{^{1}H} NMR spectrum (101 MHz, DMSO-d_6).

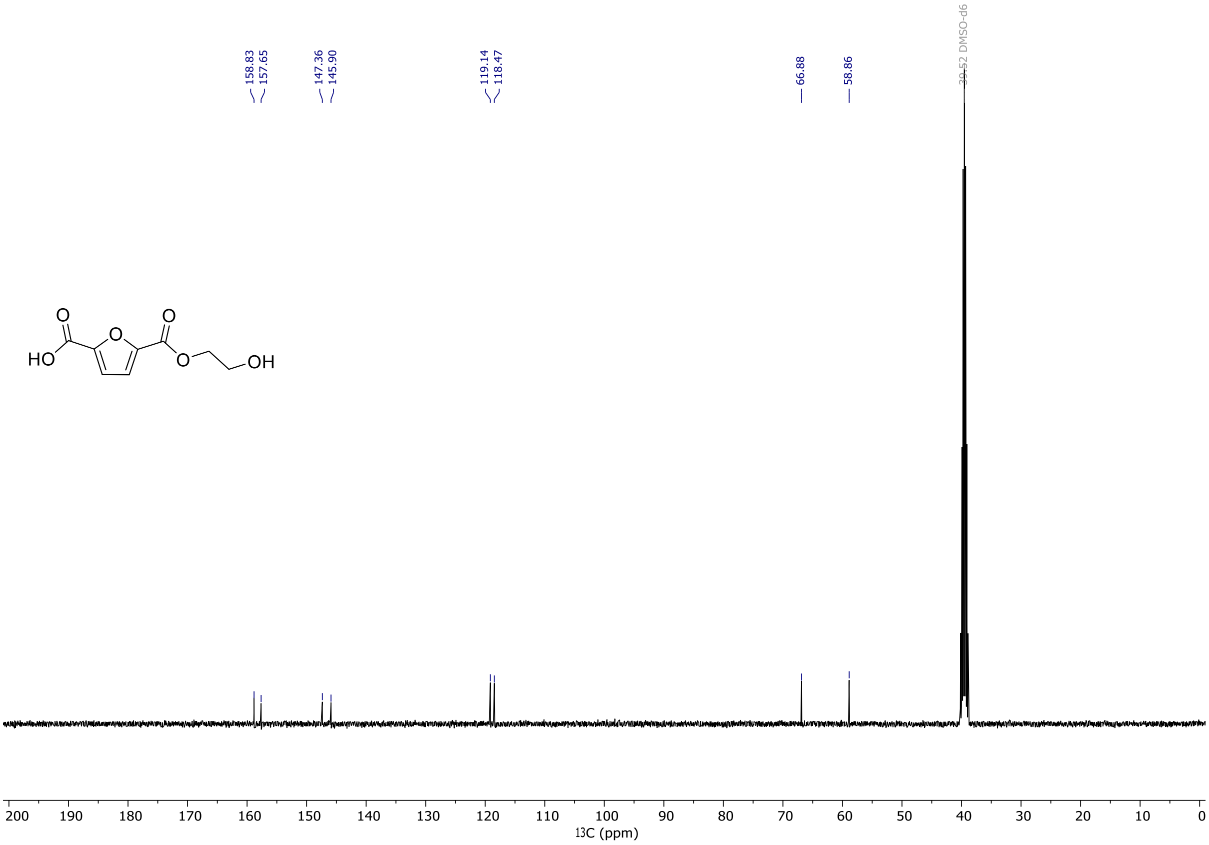

Compound $\mathbf{F_2E_1}$, ^{1}H NMR spectrum (300 MHz, DMSO-d_6).

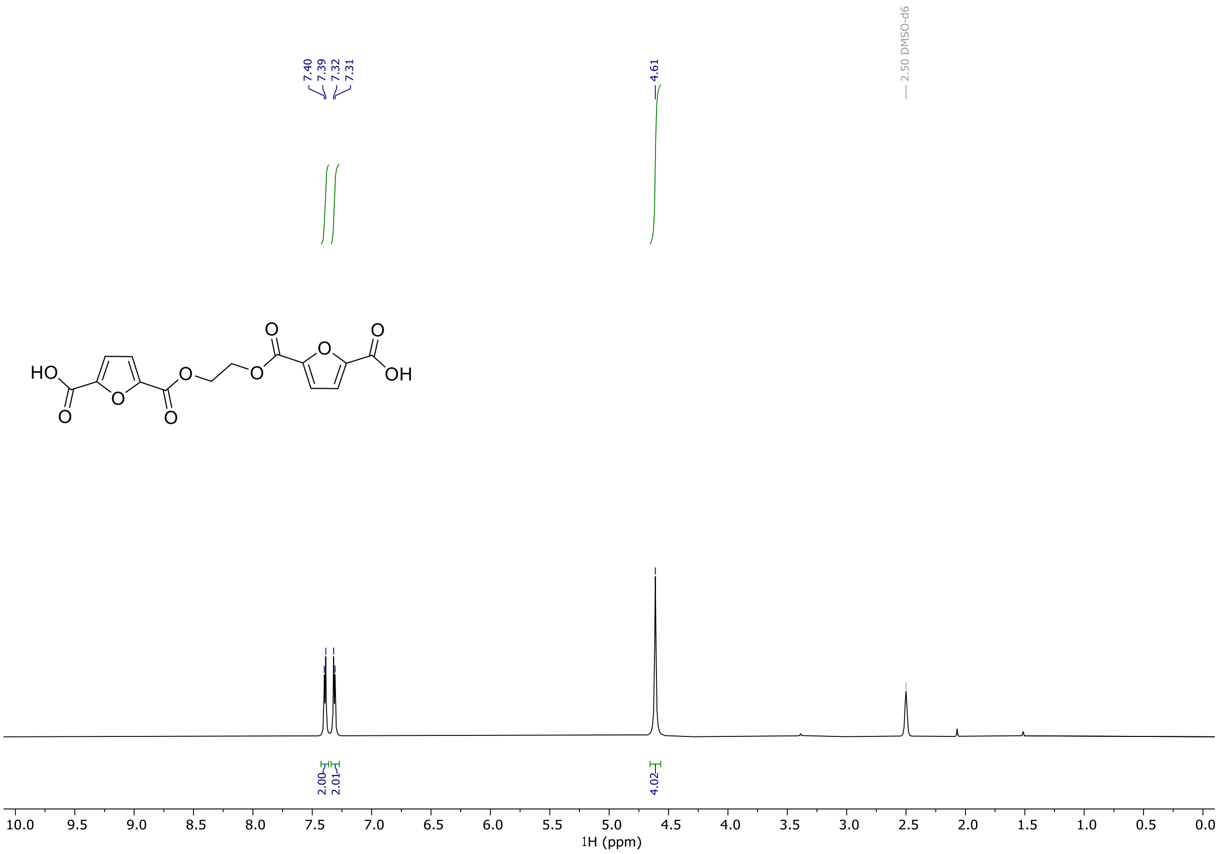

Compound $\mathbf{F_2E_1}$, ^{13}C{^{1}H} NMR spectrum (75 MHz, DMSO-d_6).

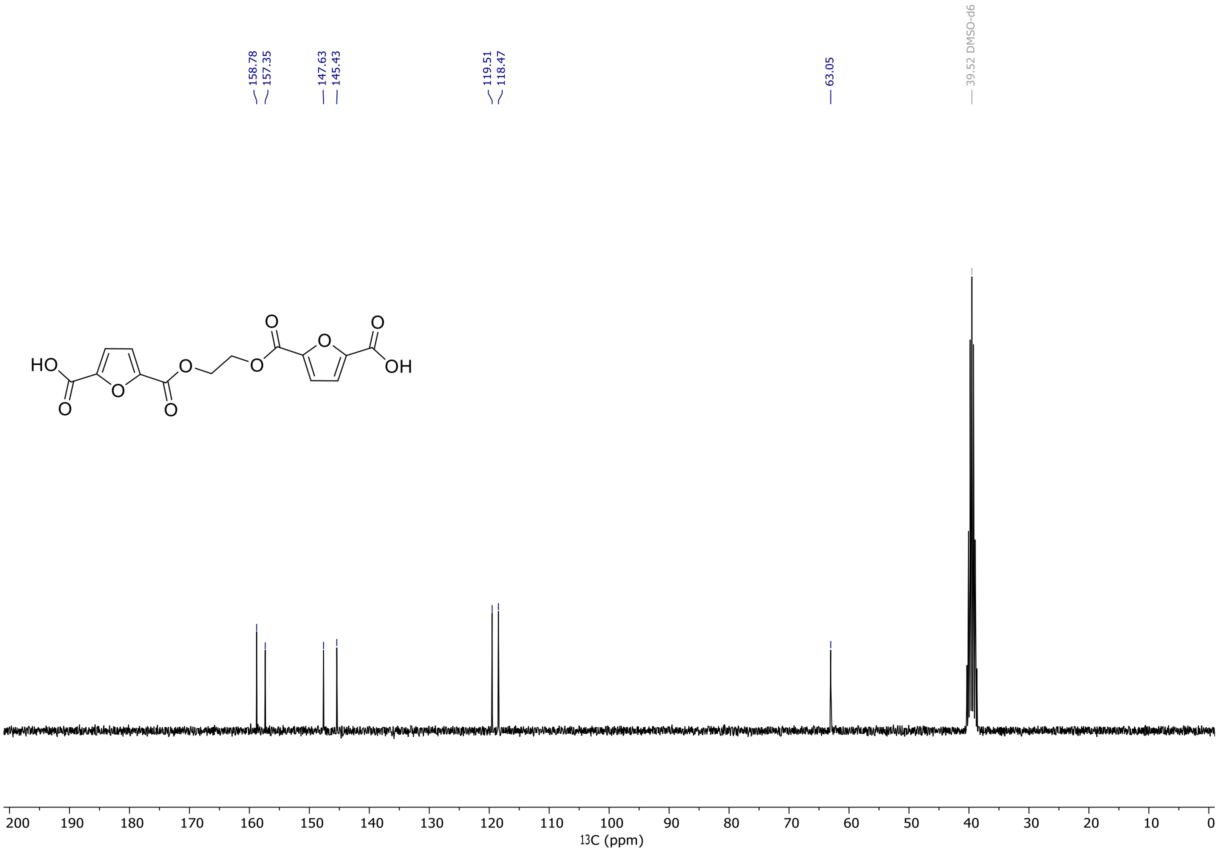

Compound $\mathbf{F_3E_2}$, ^{1}H NMR spectrum (300 MHz, DMSO-d_6).

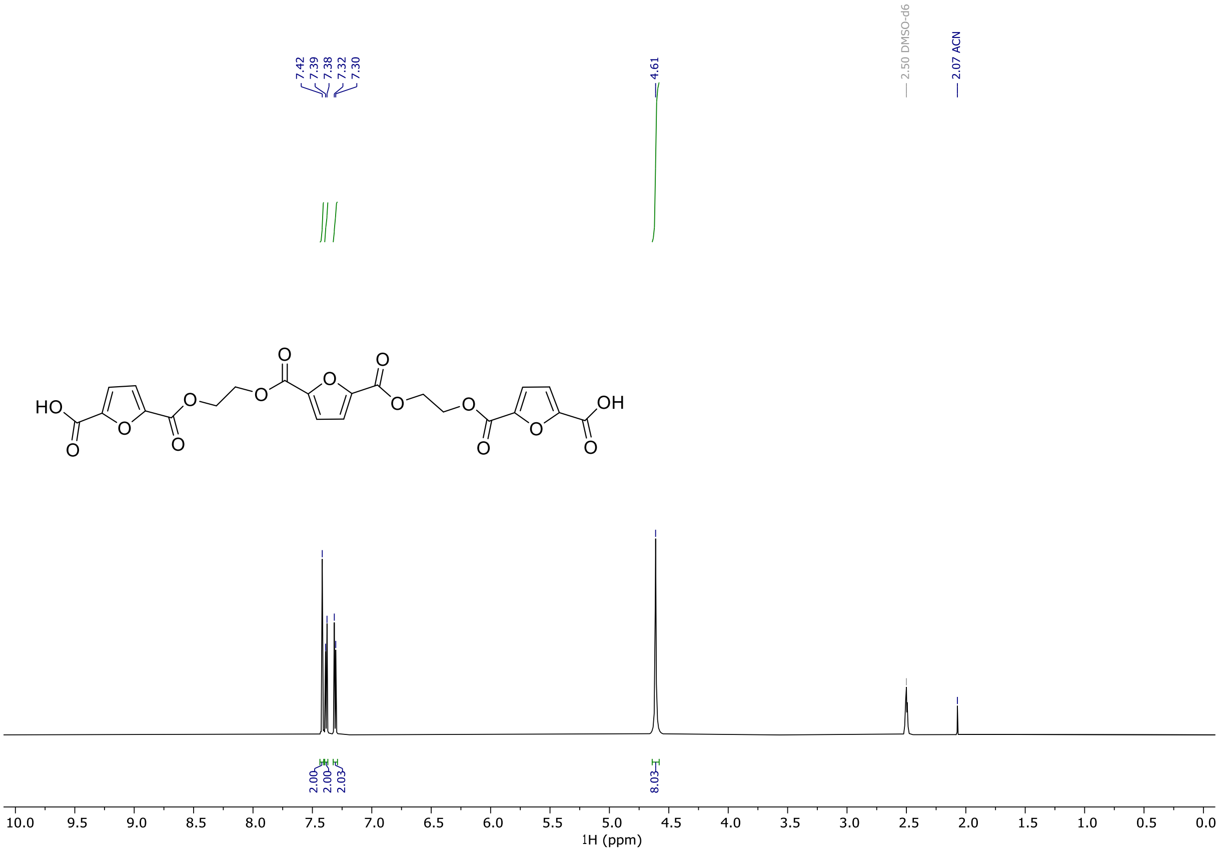

Compound $\mathbf{F_3E_2}$, $^{13}C\{^{1}H\}$ NMR spectrum (75 MHz, DMSO-d_6).

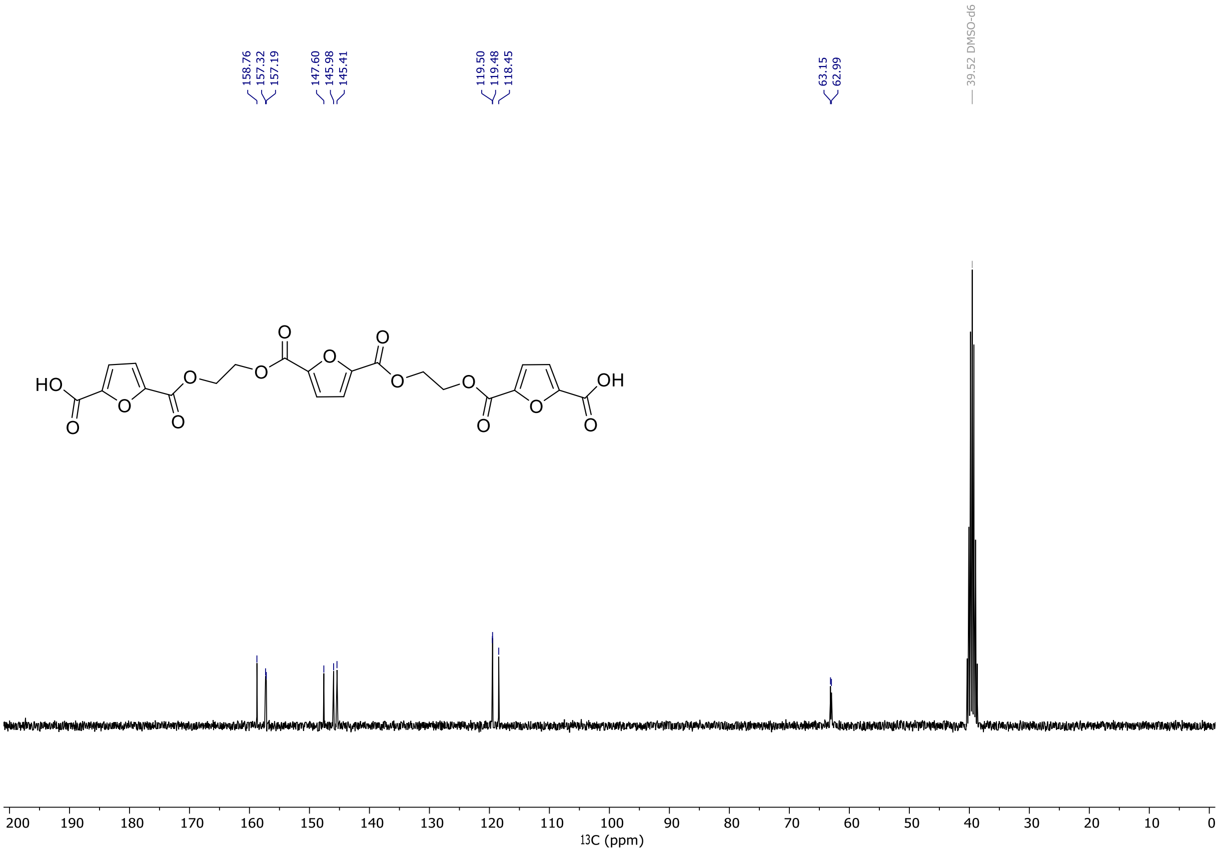

Compound **F_1B_1**, ^{1}H NMR spectrum (300 MHz, DMSO-d_6).

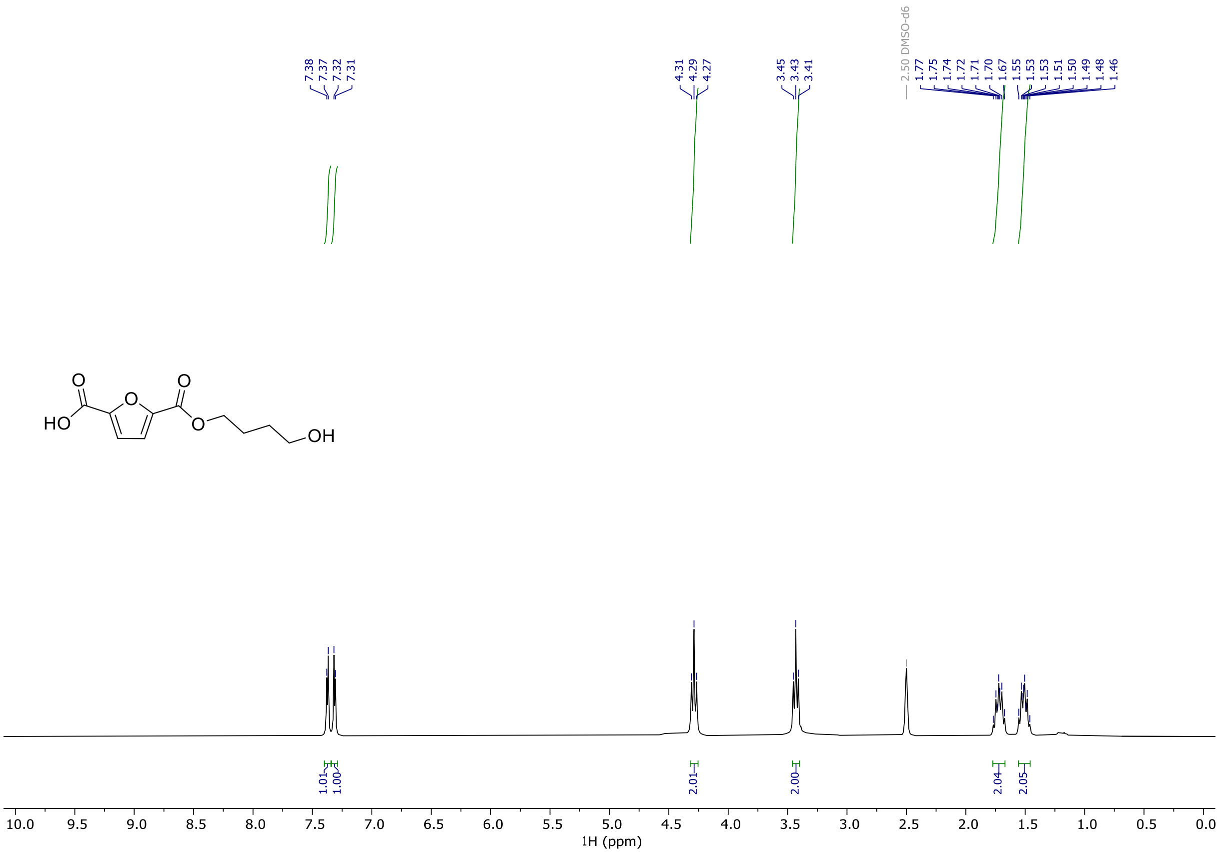

Compound **F_1B_1**, ^{13}C{^{1}H} NMR spectrum (75 MHz, DMSO-d_6).

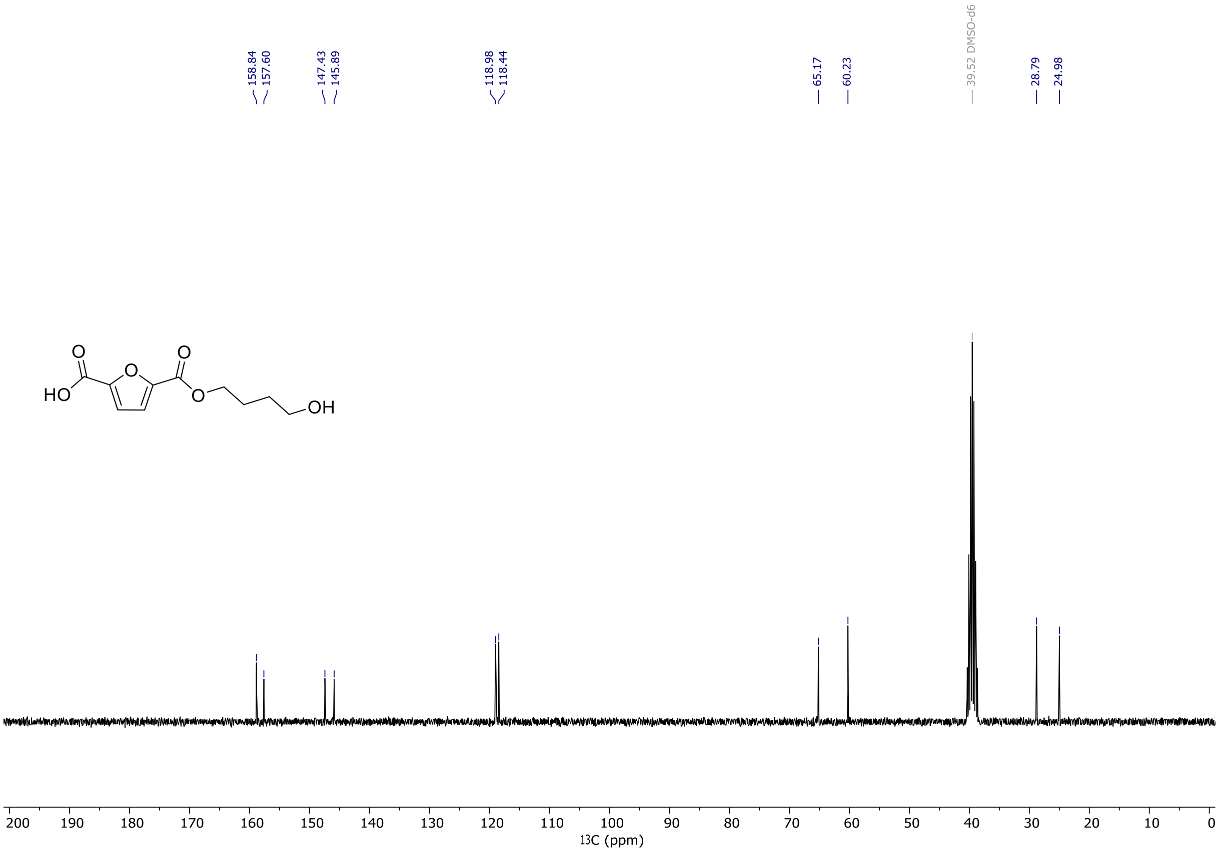

Compound $\mathbf{F_1E_2}$, ^{1}H NMR spectrum (300 MHz, DMSO-d_6).

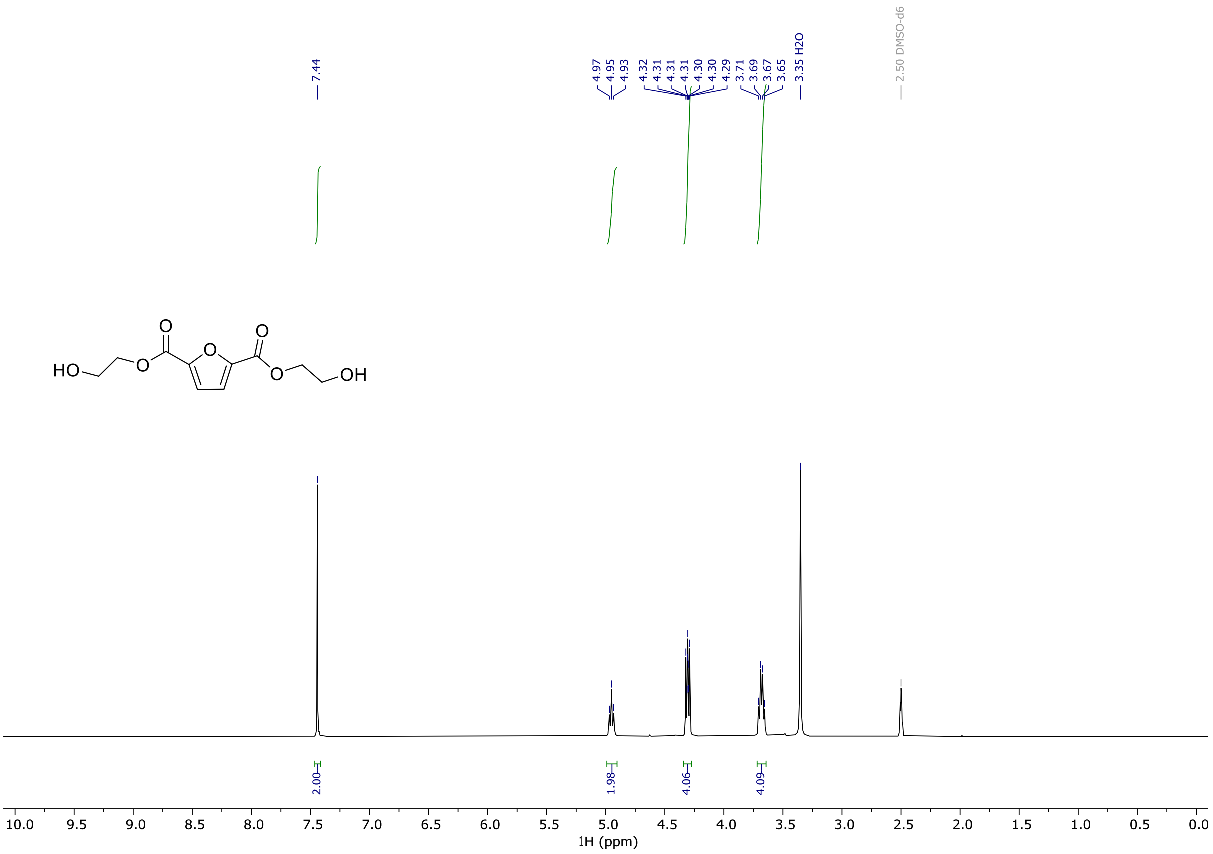

Compound $\mathbf{F_1E_2}$, ^{13}C{^{1}H} NMR spectrum (75 MHz, DMSO-d_6).

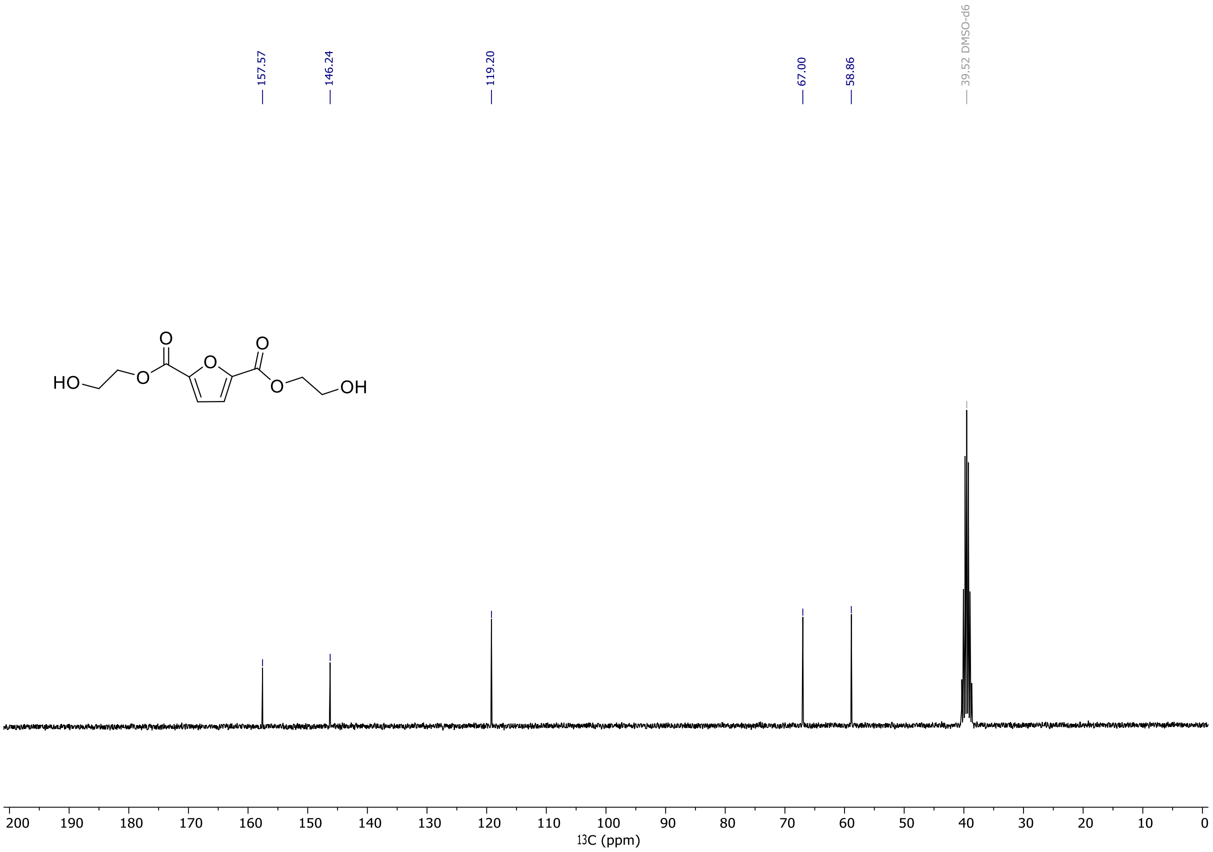

Compound **MeF₁E₁**, ^{1}H NMR spectrum (400 MHz, $CDCl_3$).

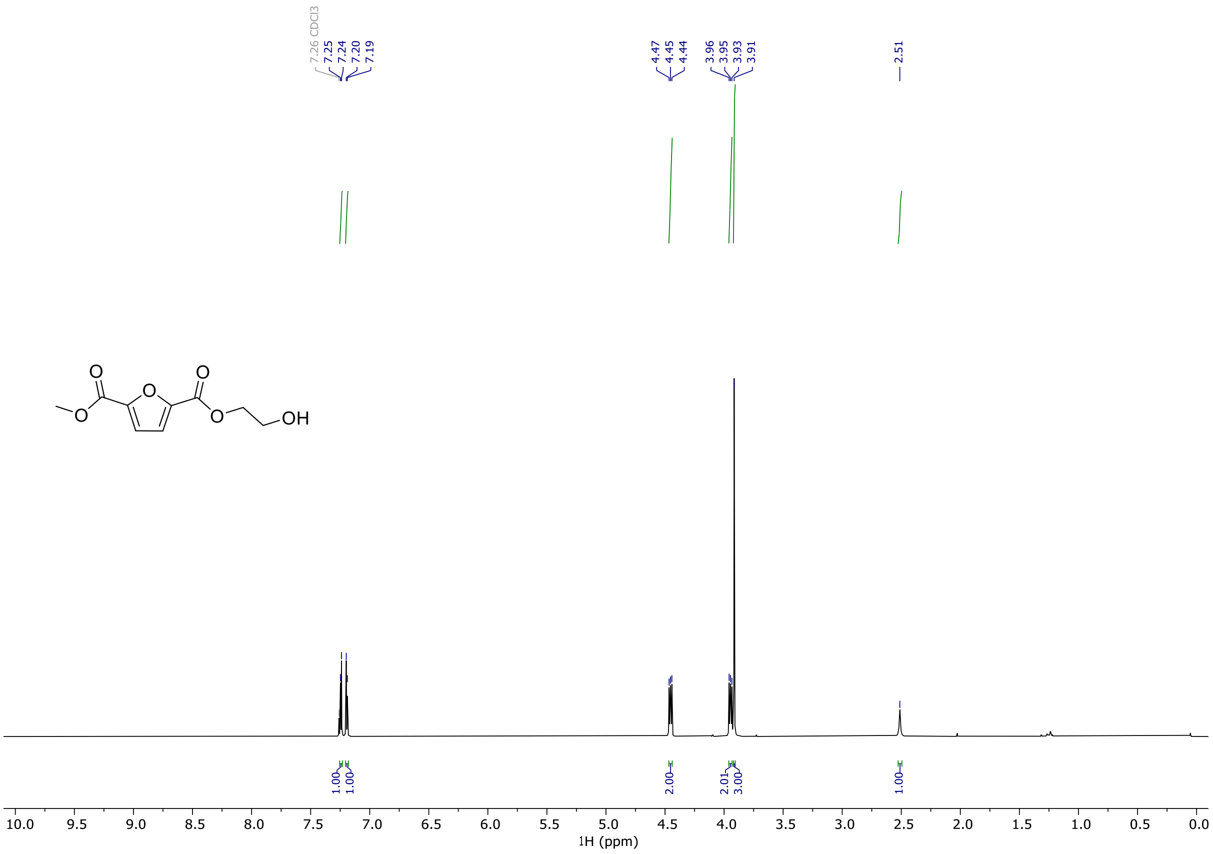

Compound **MeF₁E₁**, ^{13}C{^{1}H} NMR spectrum (400 MHz, $CDCl_3$).

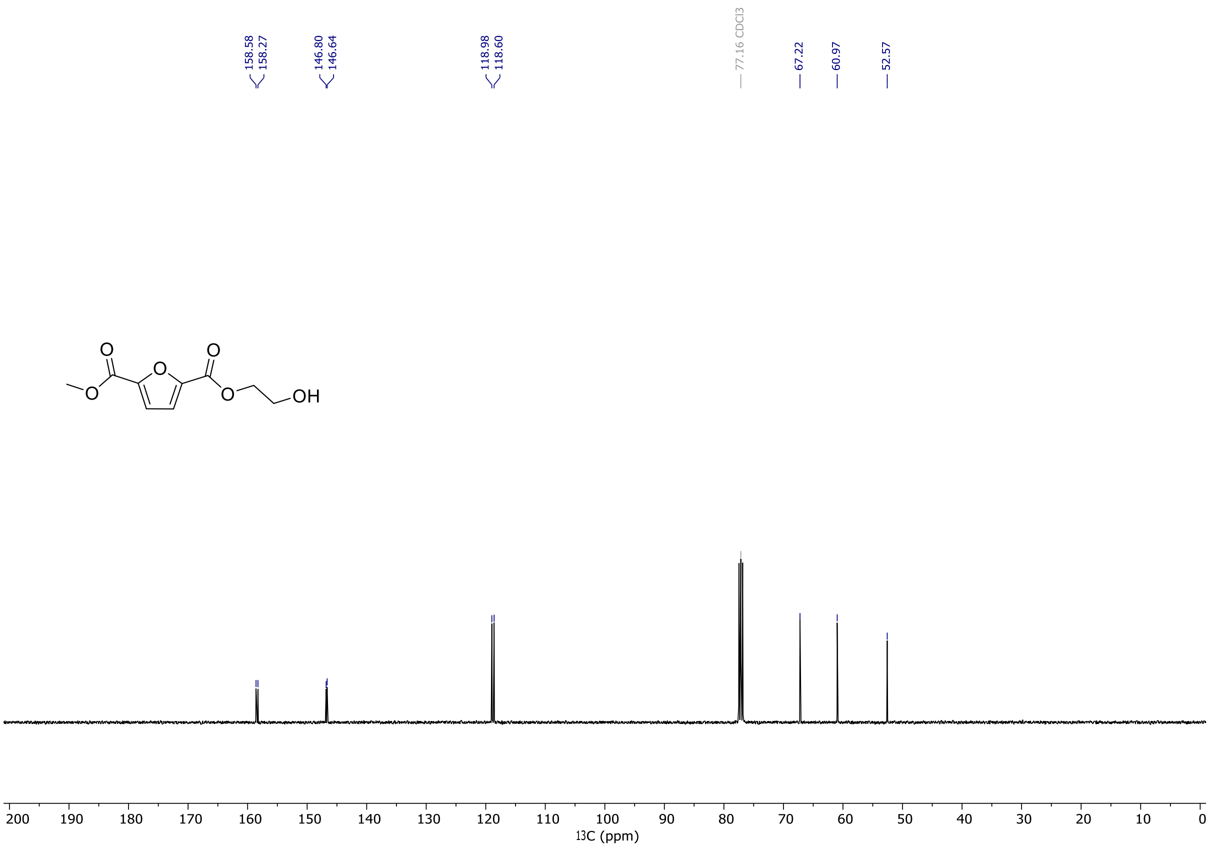

Compound **$Me_2F_2E_1$**, ^{1}H NMR spectrum (400 MHz, CD_2Cl_2).

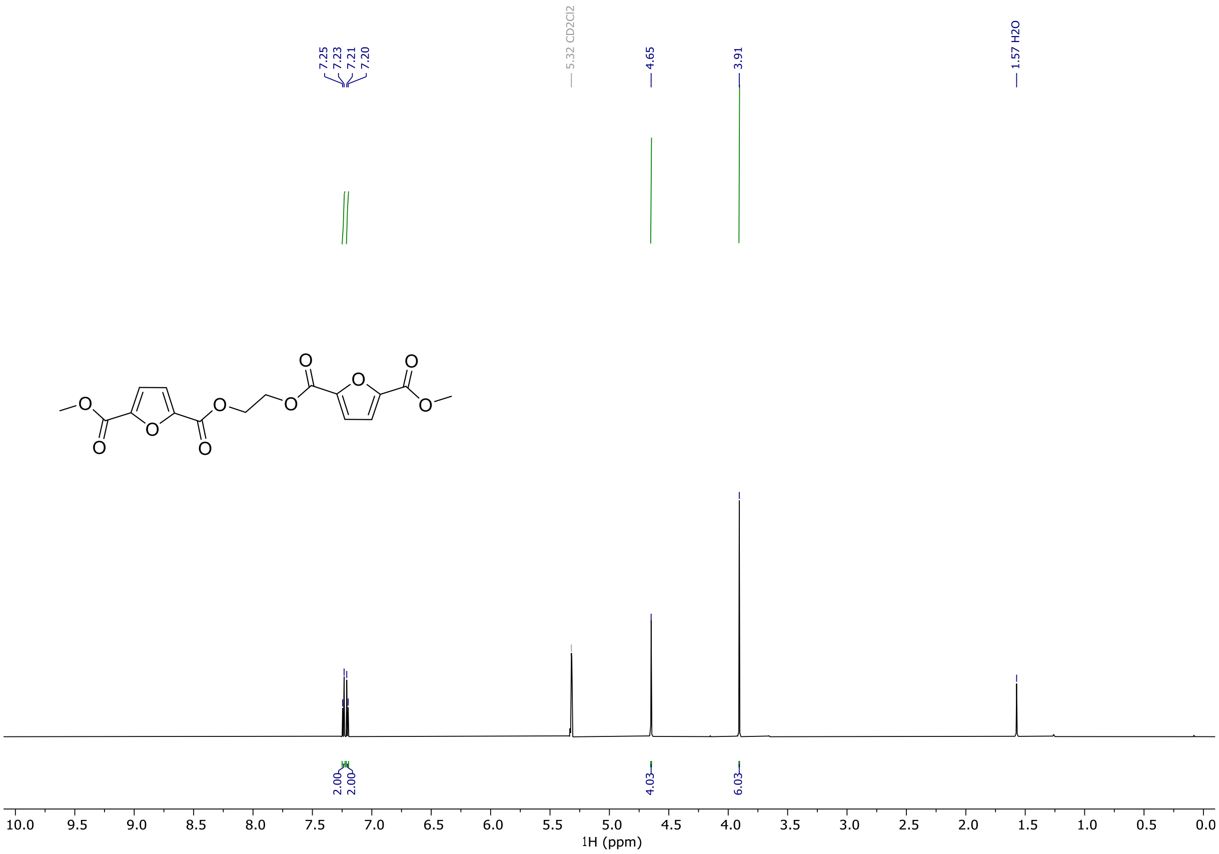

Compound **$Me_2F_2E_1$**, $^{13}C\{^1H\}$ NMR spectrum (400 MHz, CD_2Cl_2).

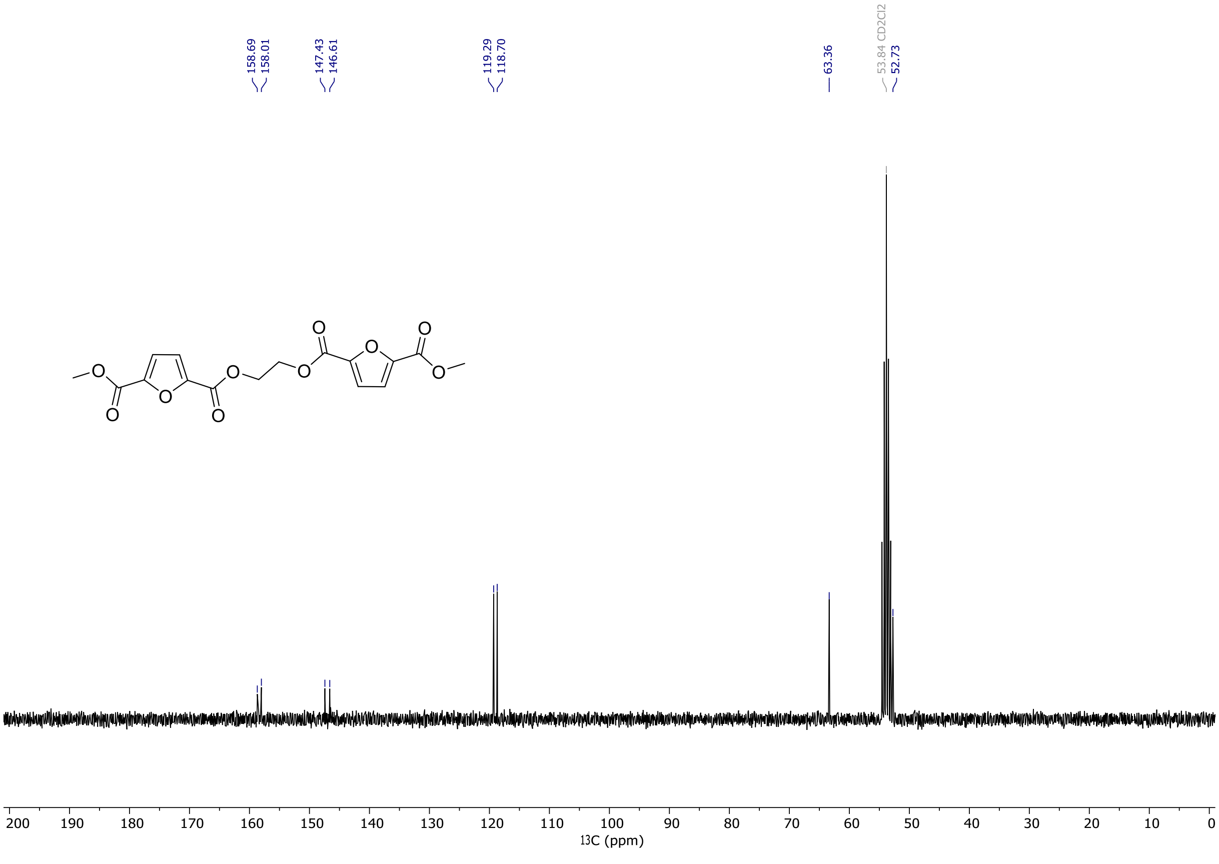

Compound **Me$_2$F$_3$E$_2$**, ^{1}H NMR spectrum (400 MHz, CD_2Cl_2)

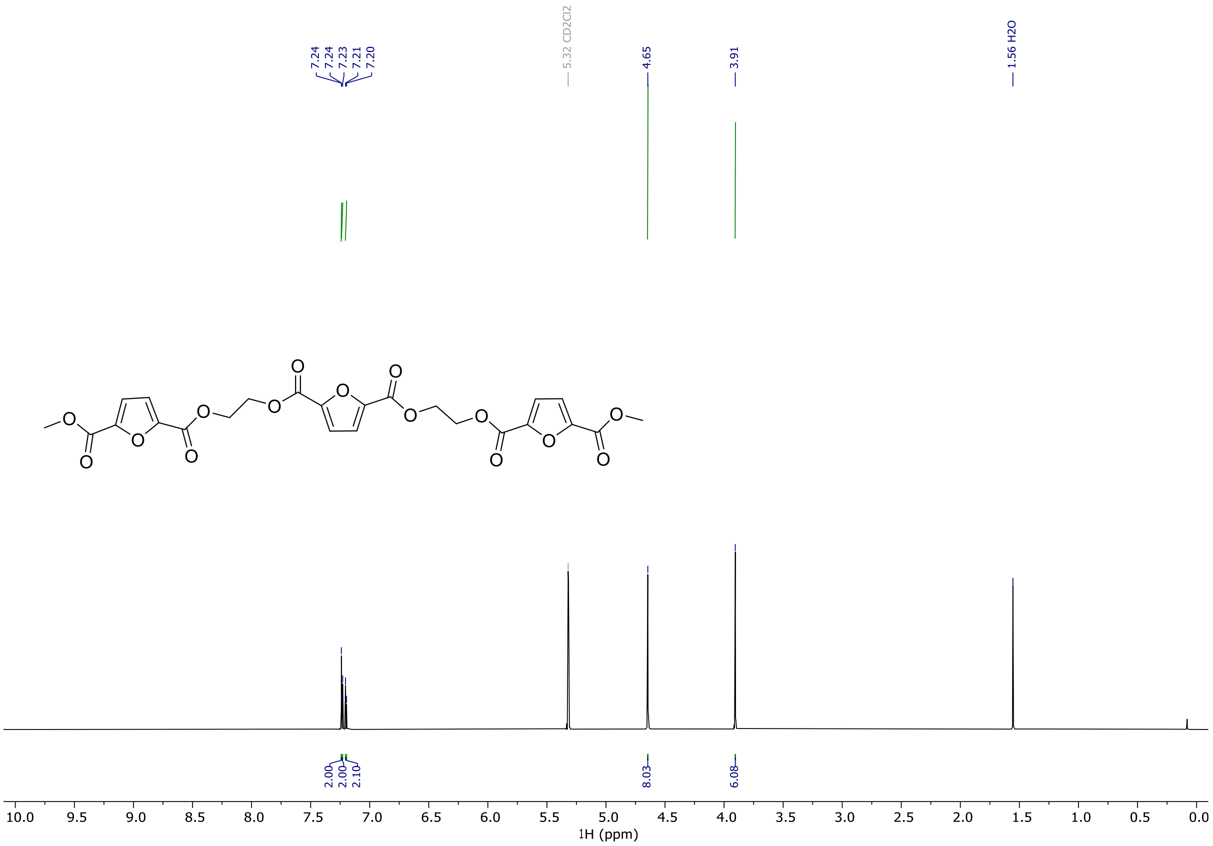

Compound **Me$_2$F$_3$E$_2$**, ^{13}C{^{1}H} NMR spectrum (400 MHz, CD_2Cl_2)

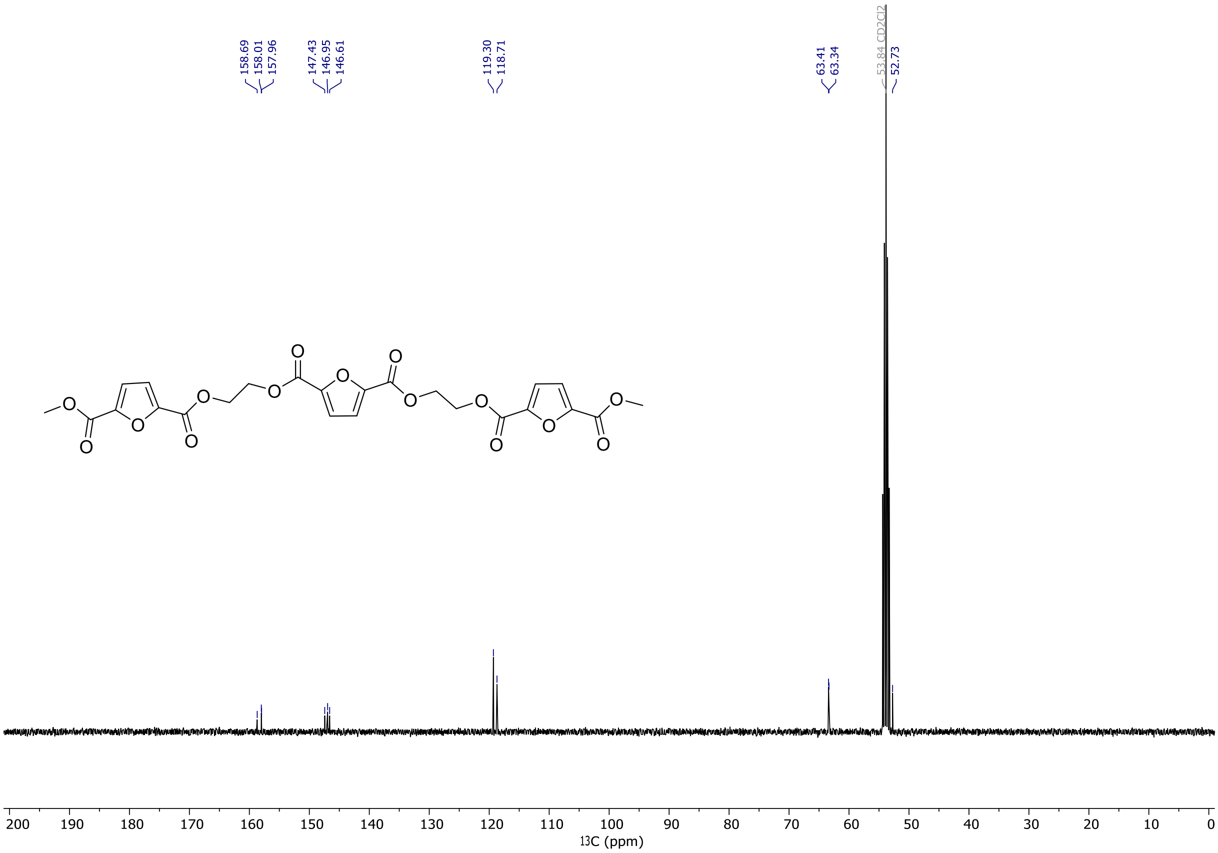

Compound **3**, ^{1}H NMR spectrum (300 MHz, $CDCl_3$).

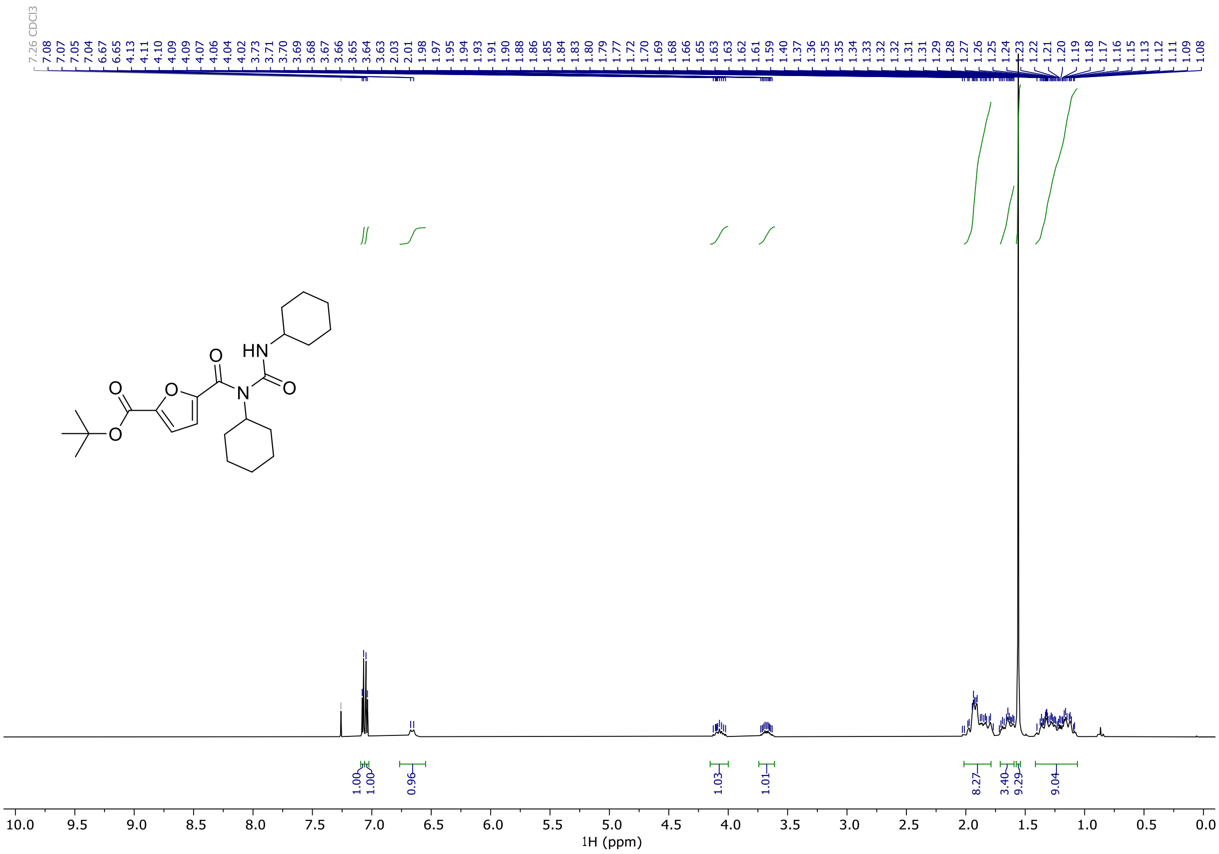

Compound **3**, ^{13}C{^{1}H} NMR spectrum (75 MHz, $CDCl_3$).

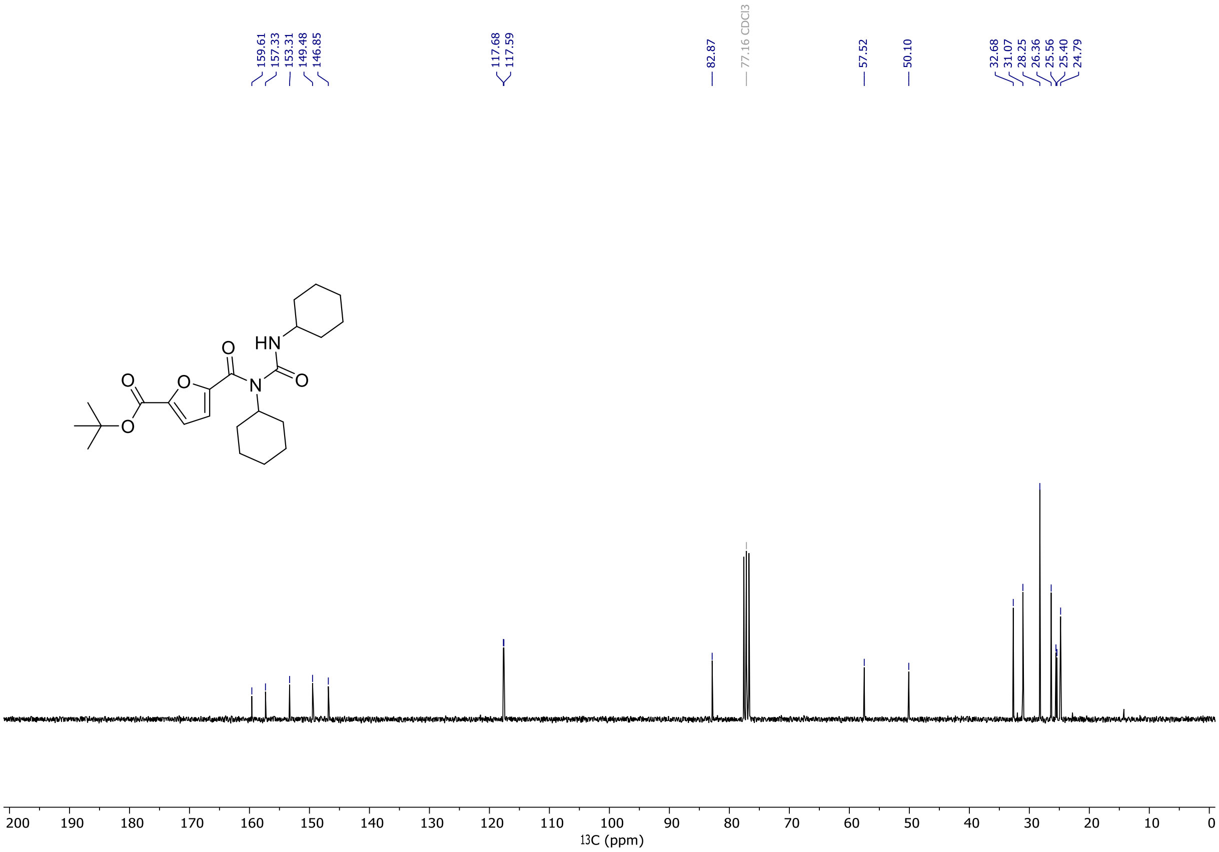

2.4 HPLC Analysis

HPLC measurements were performed on a 1260 Infinity Agilent LCMS system equipped with a degasser, binary pump, diode array detector, thermostatically controlled column compartment, autosampler and 6130 Quadruole mass spectrometer. A Zorbax Eclipse XDB-C18 (4.6 × 250 mm; 5 µm) column (Agilent Technologies) maintained at 35 °C was used to achieve a separation of the product compounds. For this purpose, a gradient must be applied using different eluent ratios of 0.1% (v/v) formic acid in water and acetonitrile (see table below for details) at a constant flow rate of 0.8 mL min^{-1}. The UV/vis detector was set to a wavelength of 260 nm to monitor the analytes, whereas the mass spectrometer was only used to confirm the signal assignment.

Table S8. HPLC analysis of PEF model compounds: Ratios between water and acetonitrile during gradient elution.

Time [min]	water [%]	CH_3CN [%]
0.0	88	12
2.0	88	12
7.0	60	40
9.0	30	70
10.0	30	70
20.0	88	12
35.0 (end)	88	12

HPLC chromatogram and ESI-(+) mass spectrum of compound **F_1E_1**.

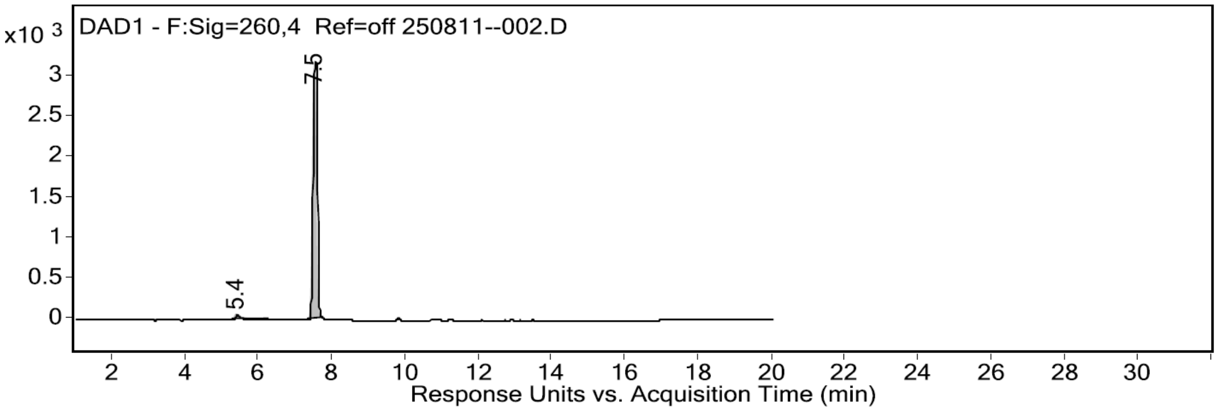

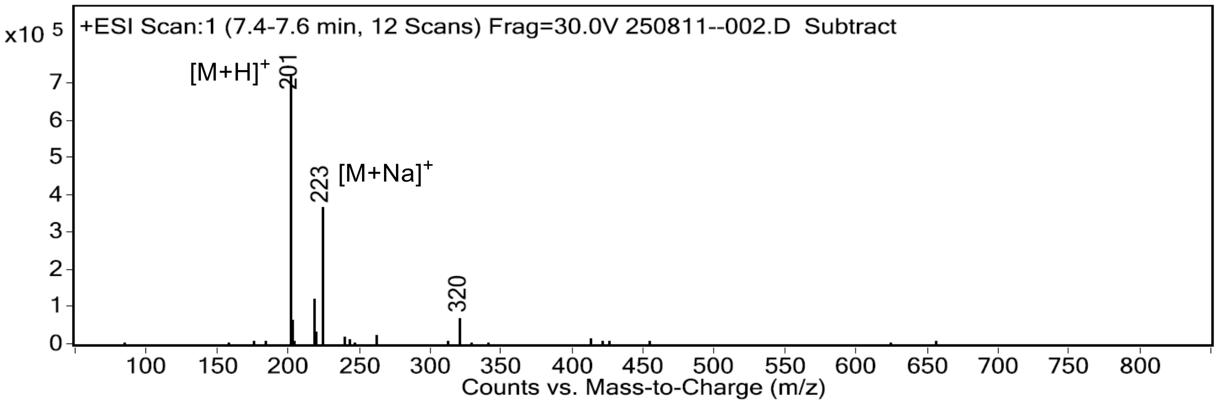

HPLC chromatogram and ESI-(+) mass spectrum of compound $\mathbf{F_2E_1}$.

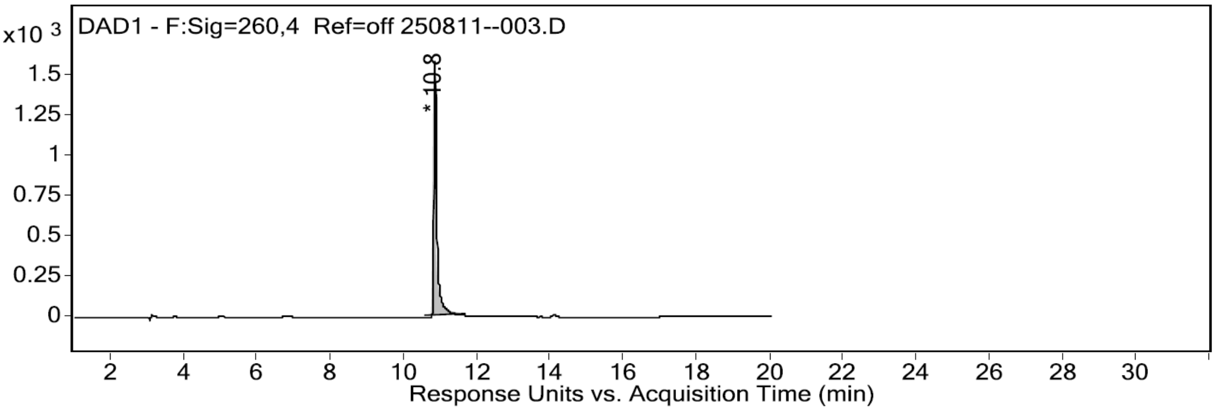

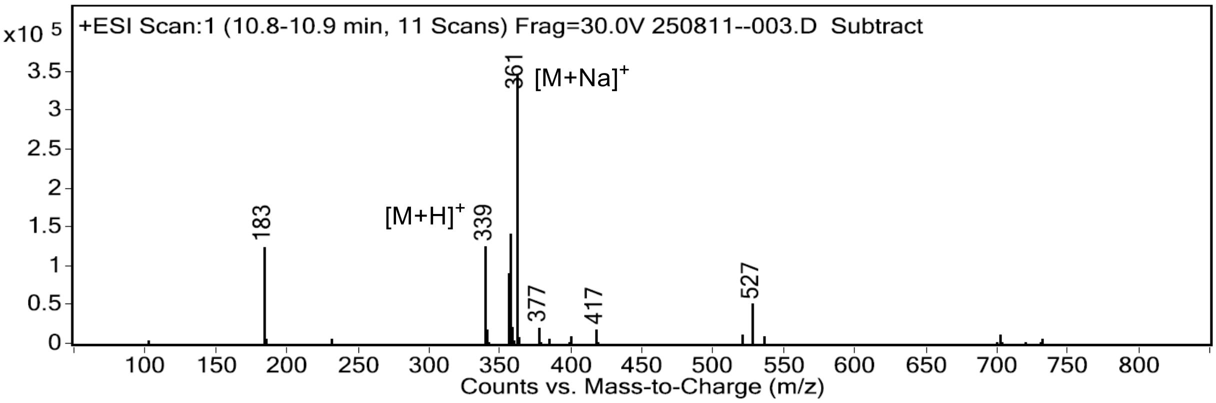

HPLC chromatogram and ESI-(+) mass spectrum of compound **F_3E_2**.

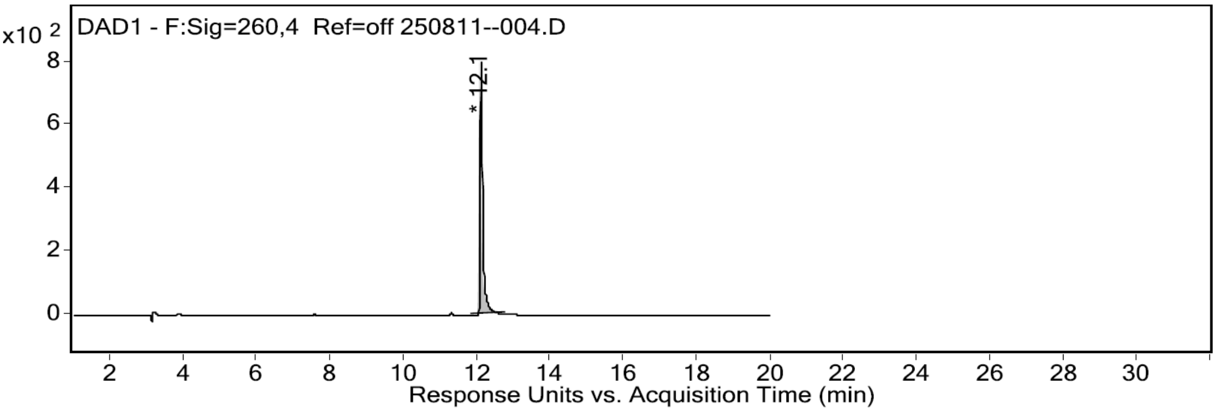

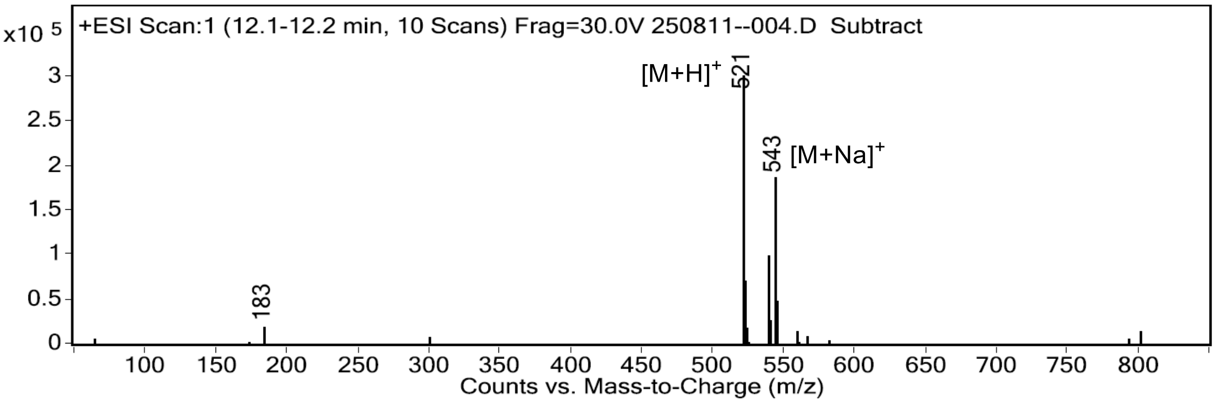

HPLC chromatogram and ESI-(+) mass spectrum of compound **F_1E_2**.

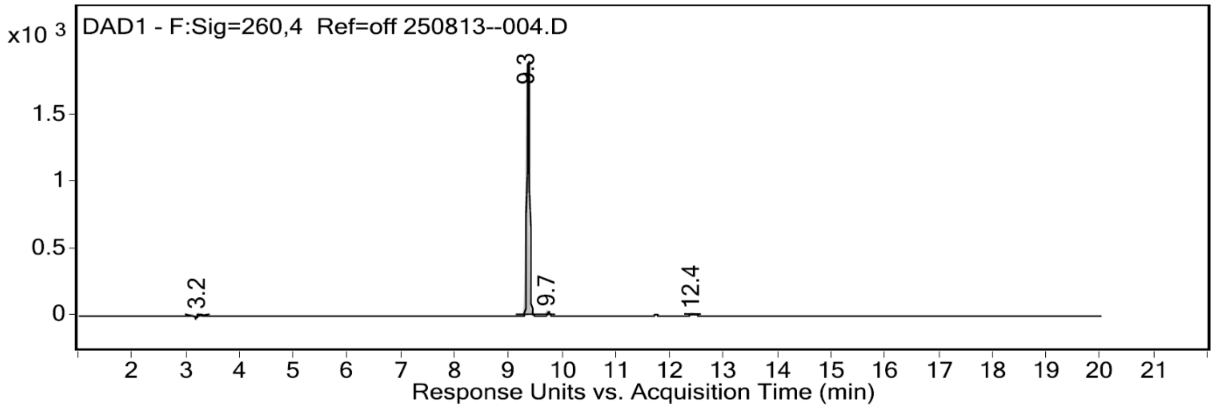

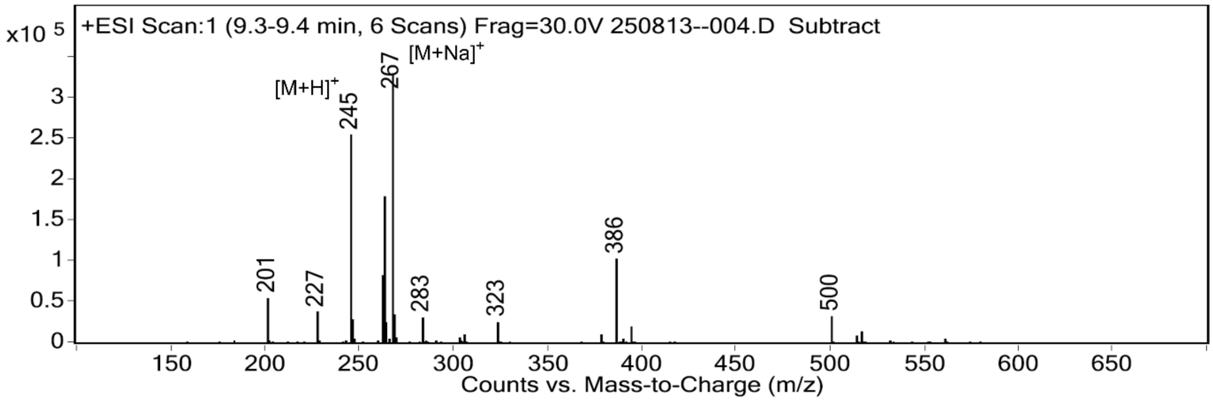

HPLC chromatogram and ESI-(+) mass spectrum of mixture of hydrolysates, *i.e.,* a mixture of compounds **FDCA** + $\mathbf{F_1E_1}$ + $\mathbf{F_1E_2}$ + $\mathbf{F_2E_1}$ + $\mathbf{F_3E_2}$.

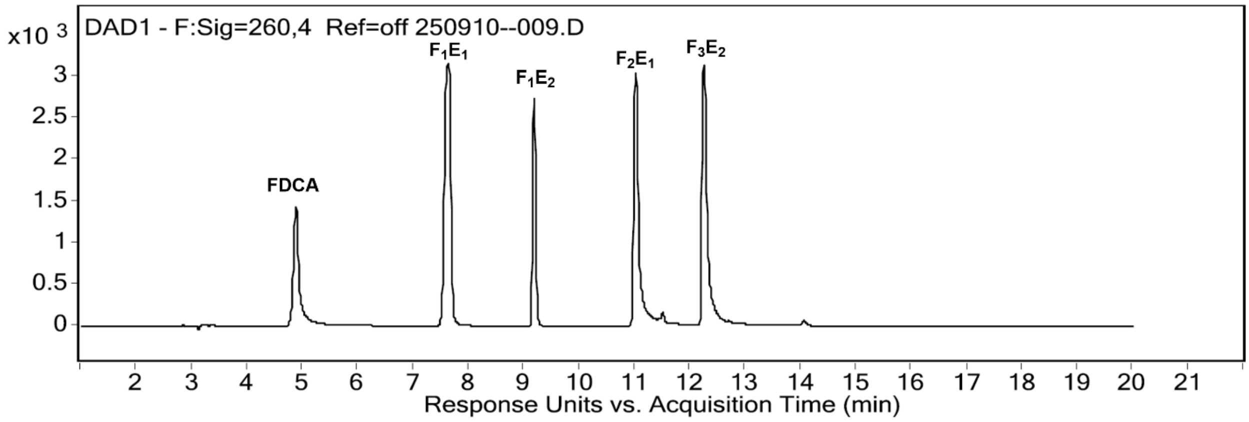

ESI-(+) mass spectrum of **FDCA** in the mixture of hydrolysates.

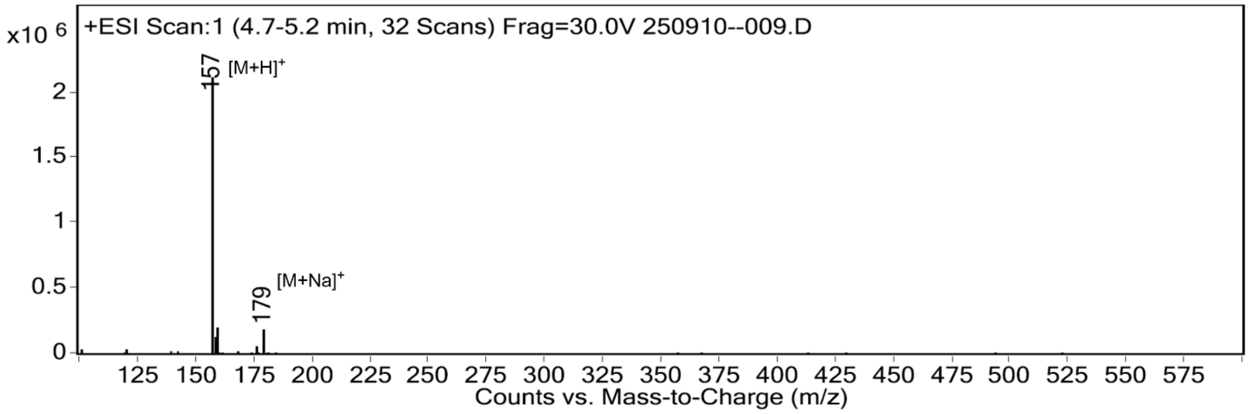

ESI-(+) mass spectrum of $\mathbf{F_1E_1}$ in the mixture of hydrolysates.

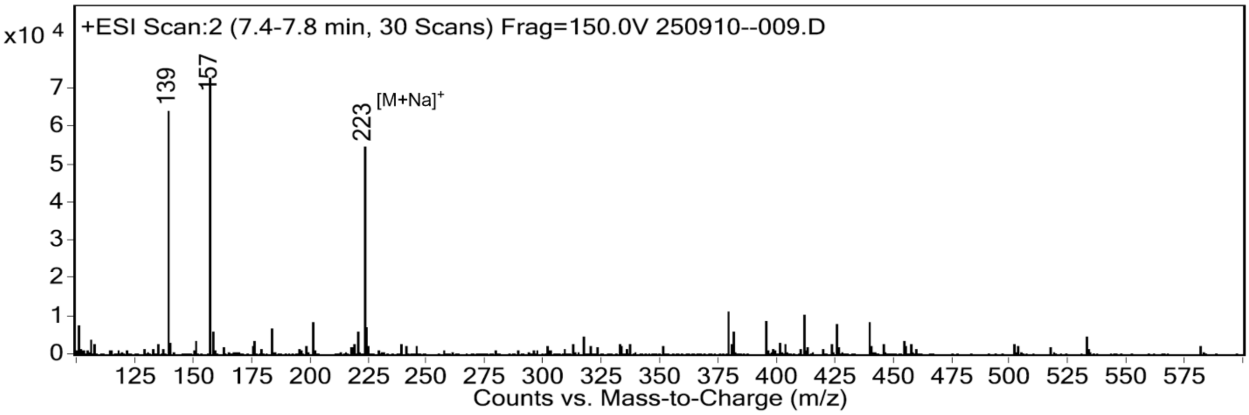

ESI-(+) mass spectrum of $\mathbf{F_1E_2}$ in the mixture of hydrolysates.

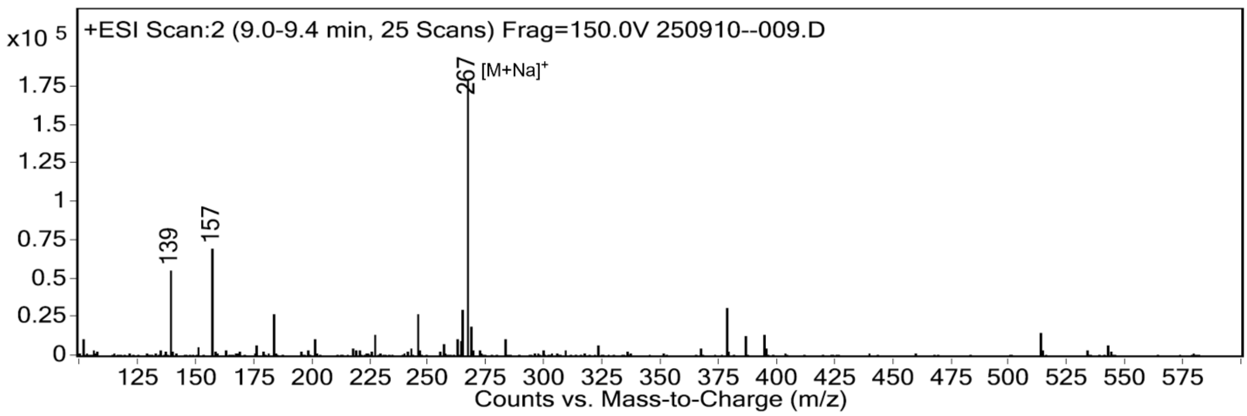

ESI-(+) mass spectrum of $\mathbf{F_2E_1}$ in mixture of hydrolysates.

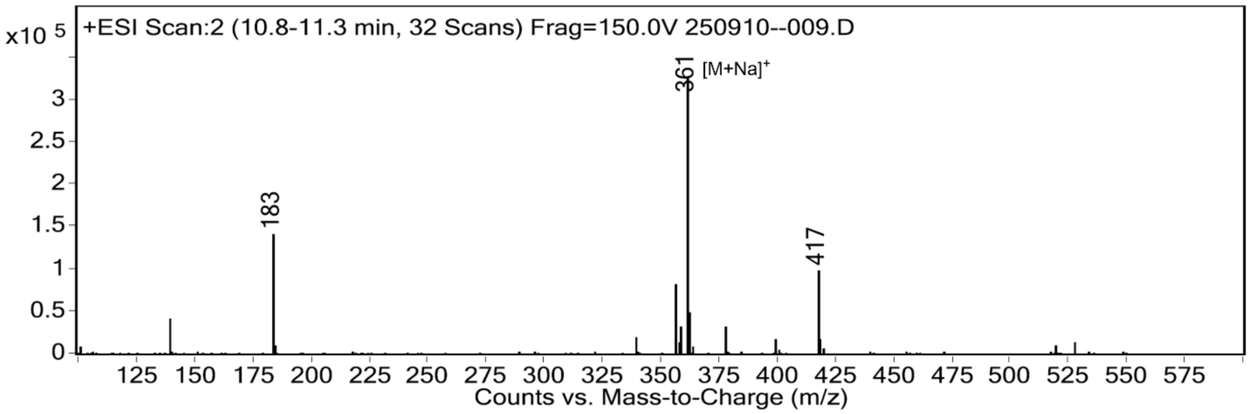

ESI-(+) mass spectrum of $\mathbf{F_3E_2}$ in the mixture of hydrolysates.

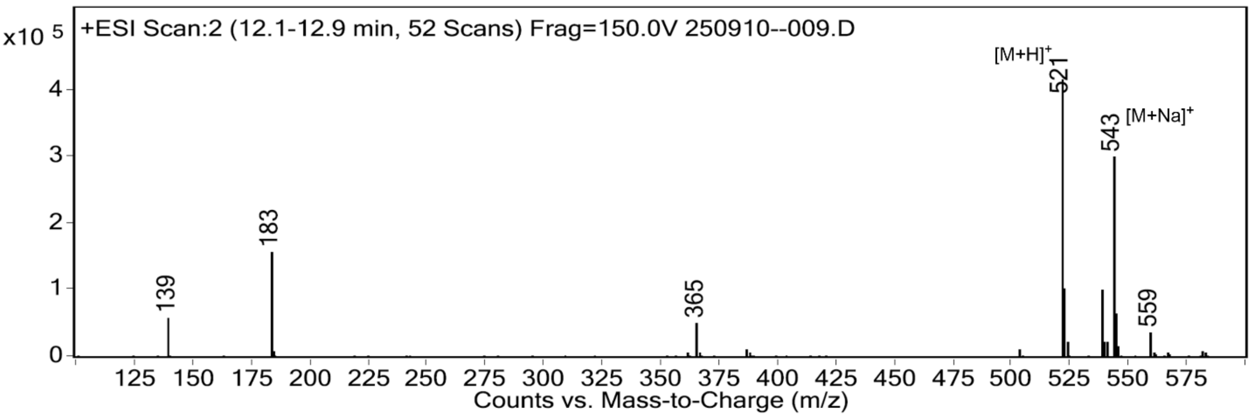

HPLC chromatogram and ESI-(+) mass spectrum of a mixture of methanolysates, *i.e.*, a mixture of compounds **Me_2F_1 + MeF_1E_1 + $Me_2F_2E_1$ + $Me_2F_3E_2$**.

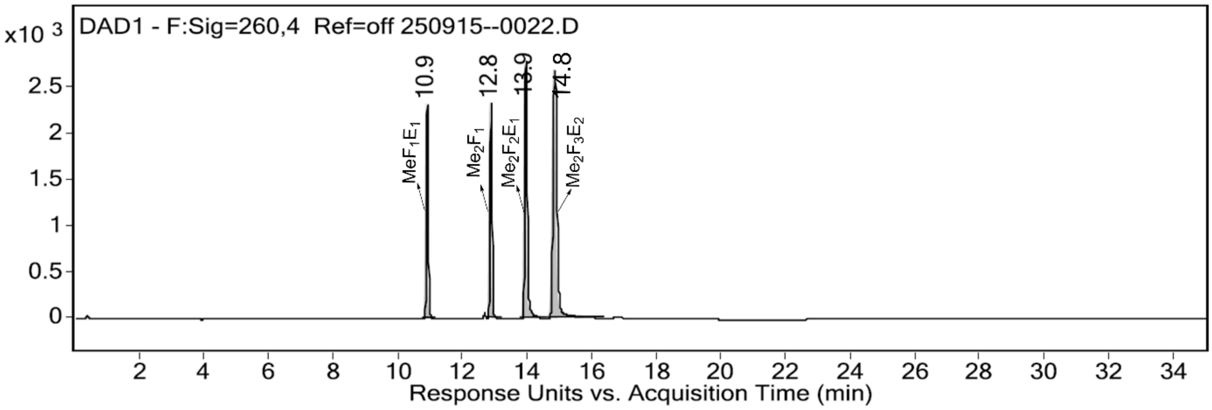

ESI-(+) mass spectrum of **MeF_1E_1** in a mixture of methanolysates.

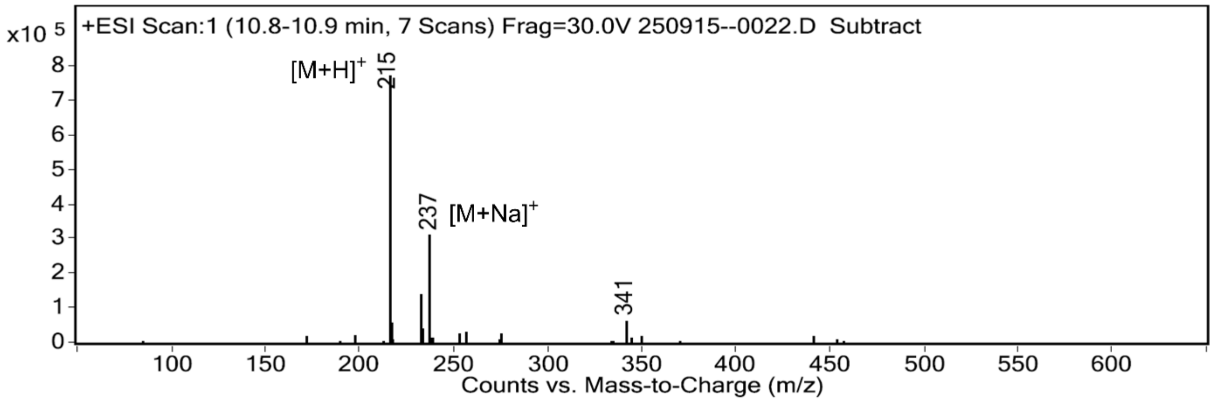

ESI-(+) mass spectrum of **Me_2F_1** in a mixture of methanolysates.

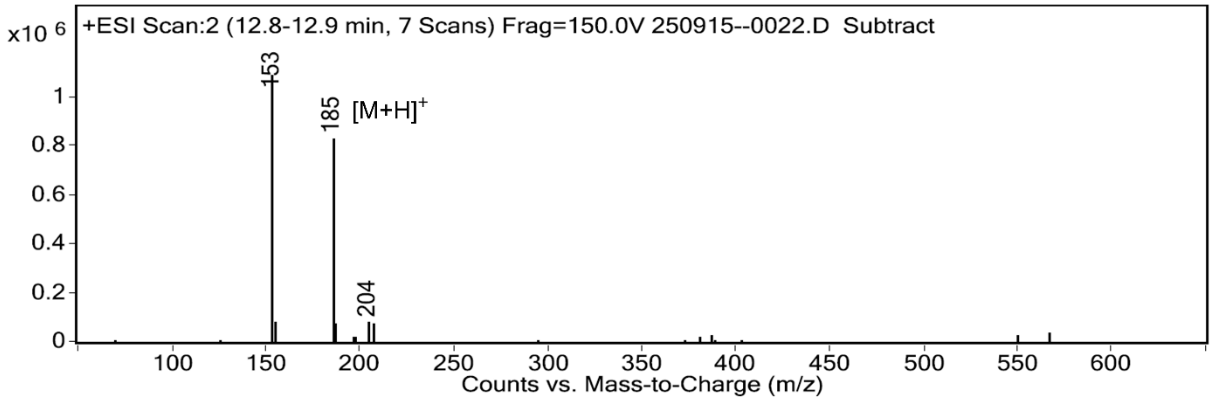

ESI-(+) mass spectrum of **$Me_2F_2E_1$** in a mixture of methanolysates.

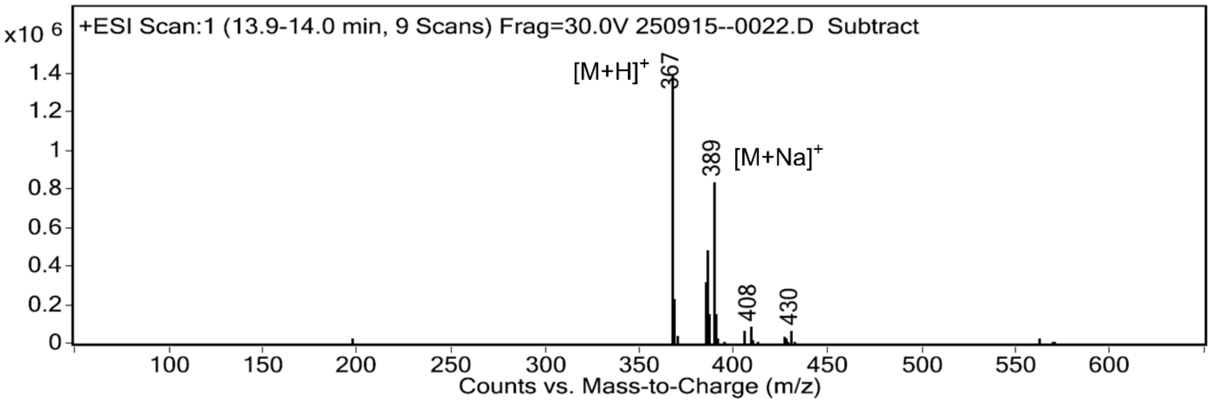

ESI-(+) mass spectrum of **$Me_2F_3E_2$** in a mixture of methanolysates.

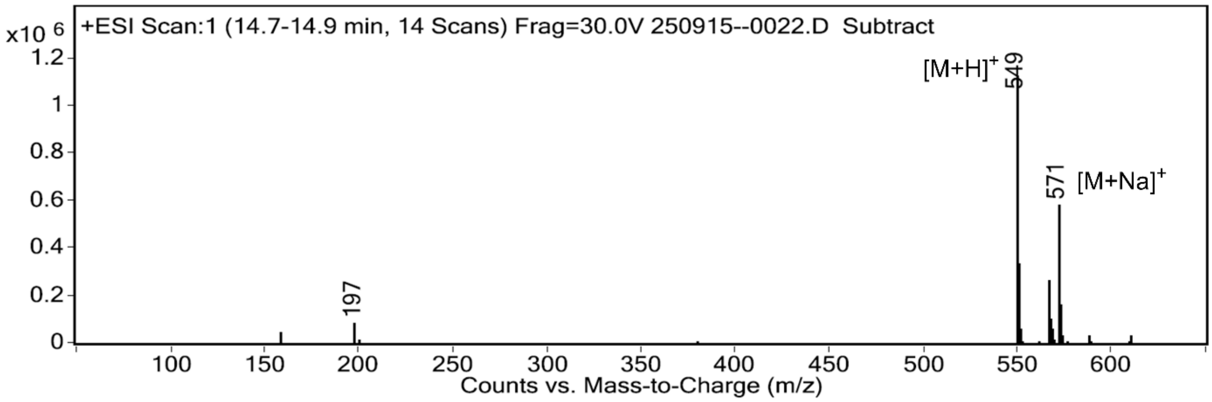

Supplement: Supplementary file 2 [file jo5c02656_si_002.pdf]
